# Precise Correction of the Dystrophin Gene in Duchenne Muscular Dystrophy Patient Induced Pluripotent Stem Cells by TALEN and CRISPR-Cas9

**DOI:** 10.1016/j.stemcr.2014.10.013

**Published:** 2014-11-26

**Authors:** Hongmei Lisa Li, Naoko Fujimoto, Noriko Sasakawa, Saya Shirai, Tokiko Ohkame, Tetsushi Sakuma, Michihiro Tanaka, Naoki Amano, Akira Watanabe, Hidetoshi Sakurai, Takashi Yamamoto, Shinya Yamanaka, Akitsu Hotta

**Affiliations:** 1 Center for iPS Cell Research and Application, Kyoto University, Kyoto 606-8507, Japan; 2 iCeMS, Kyoto University, Kyoto 606-8501, Japan; 3 PRESTO, Japan Science and Technology Agency, Kawaguchi 332-0012, Japan; 4 Department of Mathematical and Life Sciences, Graduate School of Science, Hiroshima University, Higashi-Hiroshima 739-8526, Japan; 5 Gladstone Institute of Cardiovascular Disease, San Francisco, CA 94158, USA

## Abstract

Duchenne muscular dystrophy (DMD) is a severe muscle-degenerative disease caused by a mutation in the dystrophin gene. Genetic correction of patient-derived induced pluripotent stem cells (iPSCs) by TALENs or CRISPR-Cas9 holds promise for DMD gene therapy; however, the safety of such nuclease treatment must be determined. Using a unique *k*-mer database, we systematically identified a unique target region that reduces off-target sites. To restore the dystrophin protein, we performed three correction methods (exon skipping, frameshifting, and exon knockin) in DMD-patient-derived iPSCs, and found that exon knockin was the most effective approach. We further investigated the genomic integrity by karyotyping, copy number variation array, and exome sequencing to identify clones with a minimal mutation load. Finally, we differentiated the corrected iPSCs toward skeletal muscle cells and successfully detected the expression of full-length dystrophin protein. These results provide an important framework for developing iPSC-based gene therapy for genetic disorders using programmable nucleases.

## Highlights

• A unique *k*-mer database was used to identify unique targetable regions in human genome
• A dystrophin frameshift was corrected using TALENs or CRISPR-sgRNAs in iPSCs
• Genomic integrity tests identified minimum off-target mutagenesis by the nucleases
• Dystrophin protein was detected by myogenic differentiation in the corrected iPSCs

## Graphical Abstract

**Figure:**
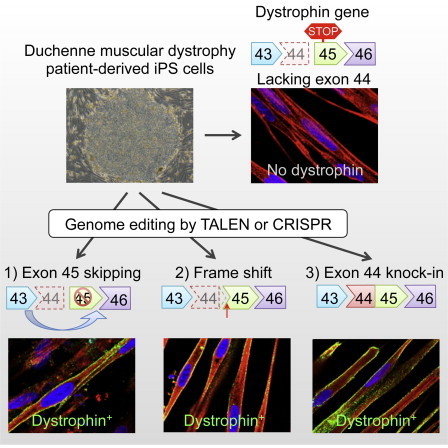

## Teaser

Using the TALEN and CRISPR-Cas9 systems, Hotta and colleagues restored the disease-causing mutation of the dystrophin gene by exon skipping, frameshift, and exon knockin approaches in Duchenne muscular dystrophy patient-derived iPSCs. Rigorous genomic integrity tests identified no severe off-target mutagenesis in the corrected iPSC clones, suggesting that both systems are promising tools for personalized gene therapy using iPSCs.

## Introduction

Duchenne muscular dystrophy (DMD) is a severe muscular degenerative disease caused by loss-of-function mutations in the dystrophin gene located on the X chromosome. The dystrophin gene consists of 79 exons, and disruption of the protein reading frame by small deletions, exon duplications, or loss of exons leads to DMD (Pichavant et al., 2011). The large size of the dystrophin gene hampers the delivery of therapeutic cDNA for gene augmentation. Therefore, the delivery of truncated microdystrophin or microutrophin by an adeno-associated viral (AAV) vector (Okada and Takeda, 2013), lentiviral vector (Pichavant et al., 2011), or *Sleeping Beauty* transposon (Filareto et al., 2013) has been investigated for DMD gene therapy. However, restoration of the full-length dystrophin protein remains challenging. An exon-skipping approach that modulates mRNA splicing patterns using antisense oligonucleotides (Aartsma-Rus et al., 2009) has shown promising results in preclinical studies, but the effects are only transient. Genomic correction using programmable nucleases is an ideal approach that can correct the mutated dystrophin gene.

The development of programmable nucleases has provided a powerful tool for modifying target genome sequences. In particular, the transcription activator-like effector nuclease (TALEN) (Hockemeyer et al., 2011) and the clustered regularly interspaced short palindromic repeat (CRISPR) and CRISPR associated 9 (Cas9) endonuclease systems (Cong et al., 2013; Mali et al., 2013) provide greater flexibility than meganucleases or zinc-finger nucleases (ZFNs) with regard to selecting the target regions of interest (Li et al., 2014). Several studies have demonstrated the effectiveness of TALENs (Hockemeyer et al., 2011; Ding et al., 2013a) and CRISPR (Ding et al., 2013b; Mali et al., 2013) in human induced pluripotent stem cells (iPSCs) for reporter knockin, gene knockout, and gene correction. In fact, corrections of disease mutation by nucleases in iPSCs have been reported for several diseases, including α1-antitrypsin deficiency (Choi et al., 2013), epidermolysis bullosa (Osborn et al., 2013), β-thalassemia (Ma et al., 2013), AIDS (Ye et al., 2014), and Niemann-Pick Type C (Maetzel et al., 2014).

Before the TALEN and CRISPR systems can reach clinical application, however, target specificity must be improved, as high off-target mutagenesis rates in human cells have been reported (Fu et al., 2013; Hsu et al., 2013; Lin et al., 2014), although some reports have shown otherwise (Smith et al., 2014; Suzuki et al., 2014; Veres et al., 2014). Since target specificity depends on the design of the target site, the properties of the DNA-binding domain, and the epigenetic status of the targeting site, the risk of off-target mutagenesis should be examined with respect to each targeting nuclease in a therapeutic setting.

Immortalized myoblasts have been used for restoration of the dystrophin protein mediated by meganucleases (Rousseau et al., 2011; Popplewell et al., 2013), ZFNs (Rousseau et al., 2011), or TALENs (Ousterout et al., 2013). However, although primary myoblasts can be derived from patients, their clonal expansion requires transformation by oncogenes such as hTERT. In contrast, iPSCs (Takahashi et al., 2007) can be isolated from patients directly and still maintain pluripotency and an unlimited self-renewal capacity. Accordingly, when conjugated with made-to-order genetic correction technologies, human iPSCs derived from a patient with a genetic disorder (Park et al., 2008; Hotta et al., 2009) might be applicable to autologous transplantation as ex vivo gene therapy (Sebastiano et al., 2011; Soldner et al., 2011; Zou et al., 2011a, 2011b).

In this study, as a proof-of-concept of such gene therapy for DMD, we performed and demonstrated genetic correction of the dystrophin gene in patient-derived iPSCs by using three different methods: (1) disruption of the splicing acceptor to skip exon 45, (2) introduction of small indels to modulate the protein reading frame, and (3) knockin of the missing exon 44 to restore the full protein coding region. We then performed comprehensive genome-wide mutation analyses to assess the risk of off-target mutagenesis in 14 iPSC clones treated according to the TALEN or CRISPR approach. Our results demonstrate that genetic correction by these approaches in patient-derived iPSCs considerably lowers the risk of off-target mutagenesis and thus holds promise for DMD gene therapy.

## Results

### Targeting a Unique Region in the Human Genome

**Figure 1:**
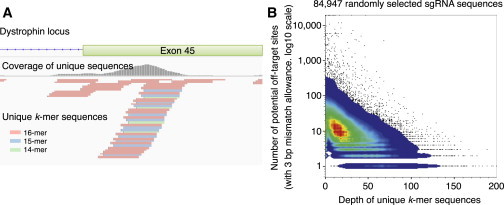
Visualization of the Unique Sequences in the Human Genome (A) Example of the mapped unique *k*-mer sequences at the exon 45 region of the dystrophin gene. The mapped *k*-mers are indicated in the bottom panel and the coverage of the *k*-mers in each base position is indicated by the gray histogram. Within this region, unique sequences of 14- to 16-mer were identified. (B) Pseudocolor dot plot for the depth of unique *k*-mers and the number of potential off-target sites. CRISPR-sgRNA targeting sequences (23 bp with “NGG” PAM, n = 84,947) were randomly selected from the human genome and the depth of the unique *k*-mers and number of potential off-target sites (with up to 3 bp mismatches allowance) for each sgRNA sequence were calculated. Note that higher depth correlated with fewer potential off-target sites. See also Table S1.

**Figure 2:**
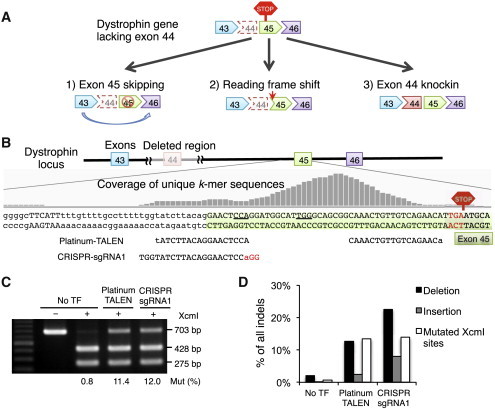
TALEN and CRISPR-sgRNA Are Equally Active for Targeting the Dystrophin Gene in iPSCs (A) The three strategies used to restore the reading frame of the dystrophin protein. (B) We designed the Platinum-TALEN pair and CRISPR-sgRNA1 within the peak of the unique *k*-mer sequences, as indicated by the gray histogram, and in front of the de novo premature stop codon, as indicated by the red hexagon. (C) The activities of Platinum-TALEN and CRISPR-sgRNA1 were analyzed by a restriction enzyme (XcmI) sensitivity assay. The XcmI digestion site (CCANNNNNNNNNTGG) was located in the spacer of the TALEN and next to the PAM sequence of the CRISPR-sgRNA1. The intensity percentage of the undigested band (703 bp) was used to calculate the mutation efficiency. (D) The frequency of deletions (black bar) and insertions (gray bar) was analyzed by deep sequencing for the region flanking the target site. The percentages of sequence reads corresponding to deletions, insertions, and mutated XcmI sites (white bar) are plotted. No TF, nontransfected control. See also Figure S2.

The risk of off-target mutagenesis by programmable nucleases is associated with the specificity of the target sequence. For example, in the 23 bp of the single-guide RNA (sgRNA) targeting region, up to 5 bp mismatches may be tolerated, which may lead to off-target mutagenesis (Fu et al., 2013; Hsu et al., 2013). To avoid this, the target sequence must be uniquely defined in the genome. Moreover, the uniqueness should be preserved when considering fragments of the sequence (i.e., 15 bp in length). Therefore, to systematically identify short unique sequences in the genome, we computationally generated all possible combinations of short *k*-mer sequences (*k* ≤ 16) and searched the human genome to determine how many identical sequences are found for each *k*-mer sequence. We then extracted the unique *k*-mer sequences only when they matched a single location (i.e., with no match to other regions; see Table S1 available online). We stacked the mapped *k*-mer sequences as a histogram to visualize their uniqueness in the sequence depth of coverage (Koehler et al., 2011; Figure 1A). We confirmed that the higher the depth of the unique *k*-mer, the lower was the number of off-target sites, with up to 3 bp mismatches allowed (Figure 1B). Therefore, the genome regions with a higher depth of unique *k*-mers were considered good candidates for targeting by programmable nucleases, and regions with no peak were not considered. The benefit of this method is that it allows one to visually identify the targetable site quickly. Based on the histogram of the unique *k*-mers, we identified the 5′ region of exon 45 in the dystrophin gene as a target site for designing TALENs and CRISPR-sgRNAs (Figures 2B and S2A).

### Generation of Integration-free DMD iPSCs

A DMD patient was diagnosed with a deletion of dystrophin exon 44 by a multiplex ligation-dependent probe amplification method. We performed a primer walking method to sequence the deleted region and identified that the deleted size was 75,484 bp (chrX: 32,215,020-32,290,503 [hg19]), including exon 44 (Figure S1A). We generated iPSC lines from fibroblasts obtained from this DMD patient using the integration-free episomal vector method (Okita et al., 2011). We chose iPSC lines that maintained a normal karyotype (Figure S1B) and expressed pluripotency markers, including TRA-1-60, SSEA5, *OCT3/4*, and *NANOG* (Figures S1C and S1D). Pluripotency was also confirmed by the in vivo teratoma formation assay (Figure S1E).

### Strategies for Dystrophin Correction

To restore the dystrophin protein reading frame in iPSCs from the DMD patient who lacked exon 44, we devised three approaches: the first was to disrupt the splicing acceptor of exon 45, as the connection of exons 43 and 46 would restore the reading frame; the second was to induce a frameshift by introducing small indels (insertions or deletions); and the third was to insert exon 44 in front of exon 45 (Figure 2A). We tested 15 pairs (five left and three right) of TALENs using the Golden-Gate assembly method (Golden-TALENs) (Sakuma et al., 2013a) and one pair of Platinum-Gate-based TALENs (Platinum-TALENs) (Sakuma et al., 2013b), which had nonrepeat-variable diresidue variations on the TAL domain to enhance the activity. We found that the E/a pair of Golden-TALENs and Platinum-TALENs showed the highest recombination activity, as assessed by the single-strand-annealing (SSA) assay, in human embryonic kidney 293T (HEK293T) cells (Figures S2B and S2C).

We also constructed five CRISPR-sgRNAs adjacent to the TALEN cleavage sites and tested the cleavage activities using the SSA assay (Figure S2C). There were no significant differences in the cleavage activities among the constructed CRISPR-sgRNAs. Therefore, we used sgRNA1 for later experiments because it was located within exon 45 and closer to the splicing acceptor than the other sequences. We then compared the activity of Platinum-TALEN and CRISPR-sgRNA1 by performing a restriction enzyme sensitivity assay, and found that both had similar activity in DMD iPSCs (Figure 2C).

When we examined the mutation patterns by deep amplicon sequencing of the target region, we found that both TALEN and CRISPR gave similar percentages of “mutated XcmI sites,” which was consistent with the XcmI-digested sensitivity assay (Figures 2C and 2D). Interestingly, CRISPR induced a slightly higher indel rate compared with TALEN (Figure 2C), which suggested that the restriction enzyme sensitivity assay is sensitive to the relative position of the nuclease-targeting site and to restriction enzyme sites that differ by only a few basepairs. We also noted that TALEN produced fewer insertions compared with CRISPR-Cas9 (Figure 2D).

### Correction of the Reading Frame Mutation Solely by Nuclease Treatment

**Figure 3:**
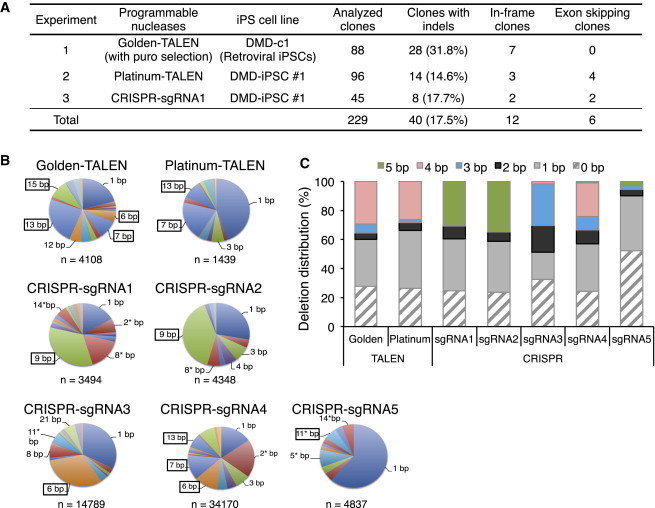
Correction of the Dystrophin Gene by Exon Skipping and Frameshifting Solely by Nuclease Treatment (A) Summary of the exon-skipping (ES) and in-frame (IF) correction approaches using the indicated nucleases without the donor template. The DMD-c1 iPSC clone was derived by a retroviral method and the DMD-iPSC #1 (CiRA00111) clone was derived by an integration-free episomal vector method. Sanger sequence analyses were performed for all picked clones to identify subclones that had indels. The number of ES and IF clones is indicated. (B) The deletion patterns induced by TALENs and CRISPR-sgRNAs were analyzed by deep sequencing of the target site. The numbers around the pie charts indicate the deletion sizes found in more than 5% of sequence reads, and the numbers with an asterisk indicate deletions that could restore the reading frame of the dystrophin gene. The boxed numbers indicate the deletion patterns flanked with local microhomologies. (C) For each of the sequence reads obtained by deep sequencing, microhomology motifs on both sides of the deletion were retrieved and the distribution of the microhomology sizes was plotted. See also Figure S3 and Table S2.

We next utilized the programmable nucleases to restore the reading frame via disruption of the splicing acceptor (exon skipping) or induction of a (3*n* + 1) bp frameshift (where *n* is a nonnegative integer) without a donor template (frameshifting). Platinum-TALEN or CRISPR/Cas9-sgRNA1 was introduced into DMD iPSCs and the subclones were screened by Sanger sequencing of the target genomic DNA (Figures S3A and S3B). As summarized in Figure 3A, a total of 229 iPSC clones were analyzed, and 40 clones showed indels at the target site. Among the 40 clones with indels, six were identified as exon-skipping (ES) clones and 12 were identified as frameshift-induced in-frame (IF) clones. We chose two clones (ES^H19^ and ES^H29^) with an 18 bp deletion spanning the splicing acceptor site of exon 45, and three clones (IF^H13^, IF^H30^, and IF^D28^) with a 1 bp insertion as IF clones with minimal alterations to the amino acid sequence (Figure S3B). We confirmed the pluripotency of the genetically corrected clones by examining their expression of *OCT3/4*, *NANOG*, TRA-1-60, and SSEA5 (Figures S1D and S3C). Functional pluripotency was also confirmed by a teratoma formation assay (Figure S3D).

### Deletion Patterns Induced by TALENs and CRISPRs

In our indel pattern analysis, we observed that several clones harbored identical indel patterns mediated by flanking microhomology motifs. To further assess the indel patterns, we isolated the genomic DNA 2–3 days after nuclease transfection (to avoid the effects of cellular expansion) and analyzed them by deep sequencing of amplicons. We observed several variations of indel patterns, but some sizes of deletions were enriched in both TALEN- and CRISPR-treated samples (Figure 3B). Computational analysis of the nuclease target site (In silico Genome Editing and Analysis Tools, https://apps.cira.kyoto-u.ac.jp/igeats/) led us to observe that microhomology motifs existed on both sides of the deletion site (Table S2), consistent with a recent report (Bae et al., 2014a). In our case, we found that the 3–5 bp microhomology-mediated deletions led to more than 30% of the deletion events analyzed (Figure 3C). This observation suggests that a cleavage site flanked by microhomology sequences can generate preferential deletion patterns, potentially via microhomology-mediated repair.

### Correction of the Full-Length Dystrophin Protein with a Donor Template

**Figure 4:**
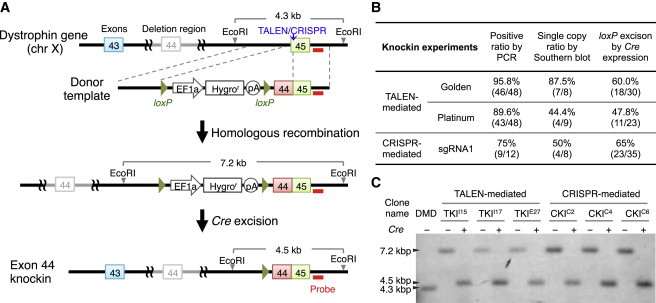
Correction of the Full-Length Dystrophin Protein by Exon 44 Knockin (A) Schematic overview depicting the exon 44 knockin strategy. Top line: structure of the dystrophin gene with the exon 44 deletion. The vertical blue arrow indicates the TALEN/CRISPR cut site and the red bar indicates the intron 45 probe used for Southern blot analysis. Note that we introduced silent mutations at the nuclease-targeting site within exon 45 of the donor template. (B) Summary of the exon 44 knockin experiments. The picked clones were first screened by genomic PCR for targeted knockin of the donor template and then by Southern blotting with EcoRI digestion and the intron 45 probe to confirm no additional integration. Successfully targeted clones were further treated with *Cre* to remove the drug selection cassette flanked by the *loxP* elements. (C) Southern blot of the knockin clones (clone names: TKI^I15^, TKI^I17^, and TKI^E27^ for TALEN-mediated knockin clones; CKI^C2^, CKI^C4^, and CKI^C6^ for CRISPR-mediated knockin clones) showing successful targeting at the designated site. Subsequent *Cre* treatment excised the *loxP*-flanked drug selection cassette. The probe in intron 45 was used to detect EcoRI-digested genomic DNA fragments. See also Figures S3B and S3C.

The deletion of exon 44 is the third or fourth most common deletion in DMD patients (Aartsma-Rus et al., 2009; Tuffery-Giraud et al., 2009); however, the breakpoints of each deletion vary among patients. In order to restore the full amino acid sequence of the dystrophin protein for DMD patients who lack exon 44, we attempted to knock in the deleted exon 44 in front of exon 45. We constructed a donor template vector to conjugate exon 45 with exon 44 to share the same splicing acceptor site together with a hygromycin-selection cassette flanked by two *loxP* sites (Figure 4A). We utilized Golden-TALEN (E/a), Platinum-TALEN, and CRISPR-sgRNA1 for the knockin experiments.

The targeting donor was coelectroporated with TALENs or Cas9/sgRNA expression vectors. After hygromycin selection and limiting dilution, we isolated several clones and screened for knockin clones by PCR analysis. Regardless of which nuclease we used, up to 90% of the analyzed clones (46/48 for Golden-TALEN (E/a), 43/48 for Platinum-TALEN, and 9/12 for CRISPR-sgRNA1) showed targeting of the donor template at the target locus. We further confirmed single-copy knockin clones by Southern blot analysis and found that approximately 44%–87% of the clones had a single copy at the targeted site (Figure 4B). We chose two clones (TKI^I15^ and TKI^I17^) mediated by Golden-TALEN (E/a), one clone (TKI^E27^) mediated by Platinum-TALEN, and three clones (CKI^C2^, CKI^C4^ and CKI^C6^) mediated by CRISPR-sgRNA1 for the subsequent experiments.

To remove the selection cassette from the knockin clones, we transiently treated the obtained clones with the *Cre* expression vector to excise the hygromycin-selection cassette. Successful excision was confirmed by genomic PCR and Southern blotting (Figure 4C). We also sequenced the chimeric exon 44-45 in the knockin clones by Sanger sequencing, and detected no extra mutations except for the five silent mutations (underscored in Figure S3B) that were designed to prevent the sequence from being recut by our programmable nucleases. In addition, we checked the expression of the pluripotency markers *OCT3/4*, *NANOG*, TRA-1-60, and SSEA5 in the knockin clones (Figures S1D and S3C).

### Analyses of Off-Target Mutagenesis Induced by TALENs or CRISPRs

The risk of undesired off-target mutagenesis is one of the most important concerns for the application of genome-editing technologies, and especially for gene therapy. We designed our experiments so that all genetically corrected clones originated from a single DMD-iPSC clone, which allowed us to distinguish preexisting genetic variations or culture artifacts in iPSCs (Laurent et al., 2011; Sugiura et al., 2014) from nuclease-mediated mutagenesis. The off-target effects were classified into two categories: target-sequence-dependent and -independent mutations. To assess the former, we searched for potential off-target sites with several mismatches in the human genome using the Bowtie program. For the Platinum-TALEN and Golden-TALEN E/a pair target sequences, we found thousands of potential off-target sites containing up to three mismatches for one side of TALEN. However, no pairs were located within 100 bp to form the FokI dimer. For the CRISPR-sgRNA1, we found five predicted off-target sites containing up to three mismatches (Figure S4A).

To investigate whether these potential off-target sites were actually mutated, we amplified the predicted off-target regions and analyzed them using the T7 endonuclease I (T7EI) assay. In contrast to the on-target cleavage, four predicted off-target sites showed no detectable mutagenesis, whereas one locus had a homozygous insertion of the AluYb9 element with a poly A tail (39-mer) (Figure S4B). The poly A stretch generated a heteroduplex when the PCR products were reannealed during the T7EI assay, resulting in digestion by T7EI even in the nontransfected control. We confirmed that the insertion of AluYb9 occurred in the original DMD fibroblasts and DMD iPSCs, but not in Caucasian-derived control iPSCs (Takahashi et al., 2007; Figure S4C).

To investigate the presence of minor mutations that were not detectable by the T7EI assay, we performed deep sequencing of the amplicons at the off-target sites. No significant indel reads were observed above the background level of the nontransfected control (Figure S4D).

**Figure 5:**
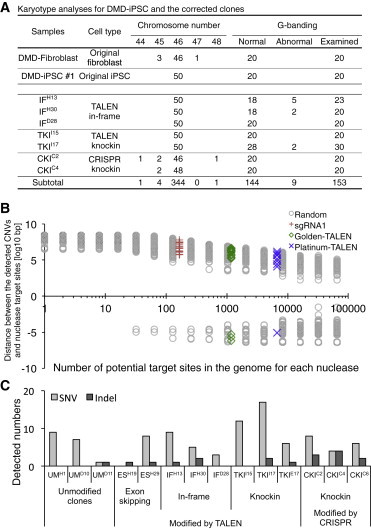
Multiple Whole-Genome Analyses Revealed No Severe Off-Target Mutagenesis (A) Summary of the karyotyping analyses of the corrected clones. Conventional Giemsa staining was used for chromosome counting (50 metaphases were counted), and high-resolution G-banding was applied to detect chromosomal rearrangements (at least 20 metaphases were analyzed). (B) SNP array analysis identified de novo CNVs in the corrected clones. To determine associations between detected CNVs and potential off-target sites, the distances between the edges of the detected CNVs and potential nuclease target sites are plotted for each nuclease (red cross for sgRNA1, green diamond for Golden-TALEN, and blue × mark for Platinum-TALEN). Since the likelihood of the distance distribution depends on how many genomic sites are selected, we also selected random genomic positions and calculated the distance for each CNV (gray circles). A zero value in the y axis indicates the edge of the CNVs and a negative value indicates the inside of the CNVs. If any given nuclease off-target site is associated with the detected CNVs, the target site should approximate zero distance. (C) The number of single nucleotide variants (SNVs) and small indels detected by exome sequencing. Only de novo SNVs and indels that did not exist in the original DMD fibroblasts or DMD iPSCs are plotted. Unmodified control clones underwent the same electroporation and subcloning process as the other corrected clones and were sequenced at similar passage numbers. See also Figures S4–S6 and Tables S3–S5.

Next, we examined whether nuclease treatment affected the chromosomal structure at the single-cell level. We first checked the karyotype of the genetically modified clones by performing a chromosome count with conventional Giemsa staining and high-resolution G-banding. Several suspected clones identified by G-banding were further analyzed by multicolor fluorescence in situ hybridization or multicolor banding. The majority of the modified cells (144/153, 94.1%) maintained a normal karyotype (Figures 5A and S5), but a few (9/153, 5.9%) showed a suspected or abnormal karyotype in three clones (out of seven modified clones analyzed; Figures S5D and S5E). Interestingly, it was previously reported that an amplification of chr20q11.21 was associated with a growth advantage for human embryonic stem cells (Amps et al., 2011). With the TKI^I17^ clone, we observed an inverted insertion at chr20q11.2 (ins(20)p11.2q13.3q12) in two of the 30 metaphases we analyzed. This insertion may have been acquired during the subcloning process.

It was previously reported that TALENs or CRISPR-Cas9 may induce up to 1 Mbp of deletions if two separated sites are targeted simultaneously (Canver et al., 2014). To elucidate whether the off-target cleavage of TALENs or CRISPR-Cas9 induced such large deletions or duplications, we investigated DNA copy number variations (CNVs) in the corrected clones by TALEN or CRISPR treatment with a high-density SNP array. We detected several CNVs compared with the reference data (human population mean), including the 75 kbp deletion of dystrophin exon 44, validating our analysis (Figure S6).

We focused on the de novo CNVs that were not present in the original DMD iPSCs, and found no major differences in the number of CNVs among the three corrected groups (Figure S6B). We employed three control clones that were subjected to similar electroporation and limiting dilution processes as the dystrophin-corrected clones, but were unmodified at the target site. To determine whether there was any correlation between the potential off-target sites and the observed CNVs, we measured the distance between the edges of the CNVs and the potential nuclease cleavage sites. The distributions for each nuclease were comparable to the corresponding numbers of randomly selected genomic loci (Figure 5B).

Furthermore, we sequenced the whole protein-coding regions of the original DMD-iPSC clone and their subclones by exome sequencing (Table S3). To assess the single nucleotide mutations associated with the treatment of programmable nucleases, we used the parental DMD iPSC as a reference and extracted the de novo mutations observed only in the offspring clones. From this analysis, we identified a few dozen point mutations (both synonymous and nonsynonymous) and several indels from the exome sequencing data (Figure 5C; Tables S4 and S5). Importantly, we successfully detected the 1 bp insertion and 18 bp deletion at the dystrophin exon 45 in the IF and ES clones, respectively (Table S5, labeled in blue). We observed slightly higher indel events (p < 0.05, one-way ANOVA) in the CRISPR knockin clones (average 3.0) compared with the unmodified controls (average 0.33). However, most of the detected indels were in the triple-repeat or GC-rich regions and were not associated with potential off-target sites, which is consistent with recent reports (Smith et al., 2014; Veres et al., 2014). In addition, there were no significant differences in the number of SNVs among the three correction approaches and the control group (p > 0.05, one-way ANOVA), which is also in agreement with recent publications (Smith et al., 2014; Veres et al., 2014). Taken together, these results show that no severe off-target mutagenesis was associated with potential nuclease-targeting sites in the dystrophin-corrected iPSC clones.

### The Dystrophin Protein Was Restored in Differentiated Skeletal Muscle Cells

**Figure 6:**
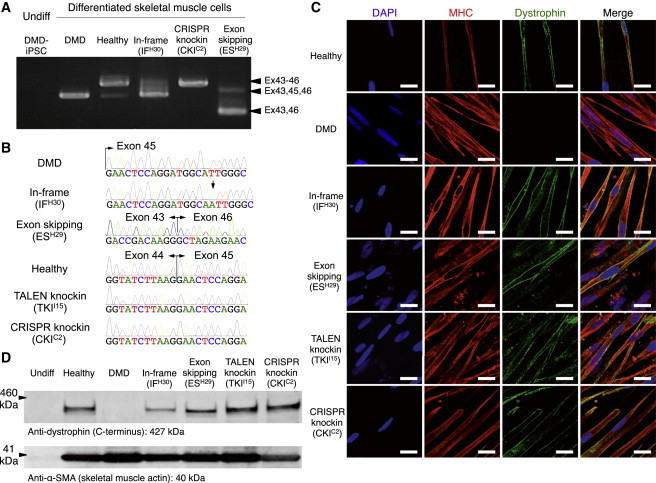
Restoration of the Dystrophin Protein in Differentiated Myogenic Cells (A) RT-PCR analysis for dystrophin cDNA from iPSCs and cells differentiated from the corrected clones toward skeletal muscle lineage. The original DMD patient and an IF clone (IF^H30^) with 1 bp insertion corresponded to the 452 and 453 bp PCR bands, respectively. The healthy control and knockin clones (TALEN-mediated TKI^I15^ and CRISPR-mediated CKI^C2^) corresponded to the 600 bp bands; and the ES clone (ES^H29^) corresponded to the 276 bp band. (B) Sanger sequence analysis of dystrophin cDNAs from differentiated skeletal muscle cells. The IF clone (IF^H30^) exhibited a 1 bp insertion (A, black arrow), the ES clone (ES^H29^) exhibited the conjugation of exons 43 and 46 due to the skipping of exon 45, and knockin clones (TKI^I15^ and CKI^C2^) exhibited the complete restoration of exon 44 in front of exon 45 as the healthy control. (C) Immunofluorescence staining of skeletal muscle cells differentiated from the corrected clones. A z axis section of the confocal microscopy image shows submembrane localization of the dystrophin protein in the healthy control and all corrected clones, but not in the uncorrected original DMD iPSCs. The cells were stained by DAPI, a marker of skeletal differentiation (myosin heavy chain [MHC]), in red and an antibody that detects the rod domain of dystrophin (DYS1) in green. Scale bar, 50 μm. (D) Western blot analysis to estimate the molecular weight of the dystrophin protein in the corrected clones. Expected molecular weight: 420 kDa for the reading-frame-corrected clone, 414 kDa for the exon-skipping clone, and 427 kDa for the exon 44 knockin clones and healthy control. An anti-dystrophin C terminus (amino acids 3661–3677) antibody was used to detect dystrophin protein, and an anti-α-SMA antibody was used as the sample loading control.

To confirm that genetic correction by TALEN and CRISPR resulted in restoration of the dystrophin gene products, we differentiated the original and corrected iPSC clones into skeletal muscle cells using our recently published method (Tanaka et al., 2013). Differentiated skeletal muscle cells were collected on day 9 of differentiation to isolate mRNA. Amplification using PCR primers spanning exons 43 and 46 revealed that the exon 44 knockin clone (CKI^C2^) had restored the full-length dystrophin mRNA to the same length as that of a healthy individual control (“Healthy”; Figure 6A). Sanger sequence analysis of the cDNA also showed the corresponding correction, where the 1 bp insertion of “A” in the IF clone (IF^H30^), the 18 bp deletion that induced the skipping of exon 45 (ES^H29^), and the knockin clone (TKI^I15^ and CKI^C2^) all led to successful expression of the inserted exon 44 together with exon 45 (Figure 6B).

Finally, to detect the restored dystrophin protein, we performed immunofluorescence staining with an anti-dystrophin antibody (Dys1), which recognizes the rod domain (amino acids 1,181–1,388) of the dystrophin protein (Figure 6C). The restored dystrophin protein was localized at the submembrane region, as expected, in all corrected clones examined. Furthermore, we performed a western blot analysis with an anti-dystrophin antibody, which recognizes amino acids 3,661–3,677, and detected bands at the following predicted sizes: 420 kDa for the reading-frame corrected clone, 414 kDa for the ES clone, and 427 kDa for the knockin clone (Figure 6D). As expected, we did not detect a band in the original DMD clone. Together, our data indicate that genetically corrected iPSCs can express the dystrophin protein once they differentiate into myogenic cells.

## Discussion

Here, we have demonstrated that three distinct methods can correct the dystrophin gene: exon skipping, frameshifting, and exon knockin. All three approaches restored dystrophin protein expression in differentiated skeletal muscle cells. However, only the exon knockin approach restored the full-length dystrophin protein. We took advantage of the ability to expand iPSCs limitlessly and achieved a high percentage of knockin events by incorporating a drug selection system (up to 84% in the present study). Based on its precision and efficacy, we conclude that the knockin approach is preferable for correcting the dystrophin gene in iPSCs.

Regarding the nuclease specificity, both TALENs and CRISPR-sgRNA can bind to DNA despite a few base mismatches (Hsu et al., 2013). Therefore, it is critical to target a unique region in the genome with a minimal number of off-targets, as otherwise multiple targets may be cleaved. Several web-based programs can be used to search for off-target sites with a given target sequence region (e.g., CRISPR Design Tool [Hsu et al., 2013], Cas-OFFinder [Bae et al., 2014b], and E-CRISP [Heigwer et al., 2014]). However, these programs provide the predicted number of off-target sites only within a small region (typically ∼500 bp) at any given time. Our unique *k*-mer approach allows the visualization of targetable regions in the entire genome, so users can select the targetable region(s) before checking the number of off-targets with other programs.

The risk of off-target mutagenesis is one of the most important obstacles to the therapeutic use of programmable nucleases. We performed a T7EI assay and amplicon deep sequencing to detect rare mutations at the target site, but did not detect an increased mutation rate from our results. To further assess the risk of target-sequence-independent off-target mutations, we employed combinations of rigorous genome-wide mutation analyses, such as the G-band for karyotyping, SNP array for detecting CNVs, and exome sequencing for searching SNVs and small indels. Since none of these methods alone sufficiently covers the large spectrum of mutations (from the single-nucleotide level to the chromosome level), it is important to combine several methods before applying gene therapy.

To achieve a therapeutic effect with genetically corrected iPSCs for an autologous ex vivo gene therapy approach, we must still overcome several hurdles, such as the successful transplantation of iPSC-derived myogenic cells. Since MYOD1-induced muscle cells from iPSCs have the ability to fuse (Goudenege et al., 2012; Tanaka et al., 2013), a corrected copy of the dystrophin gene may be able to contribute to an entire myofiber. Moreover, for long-term repopulation, the differentiation of iPSCs toward muscle progenitor cells (i.e., satellite cells) could be ideal for restoring damaged muscle in DMD patients (Darabi et al., 2012). In addition, an immunogenic response to the newly corrected gene product is possible (Mendell et al., 2010), although the response may be hindered by transient immunosuppression.

In summary, we have demonstrated the restoration of the dystrophin protein in patient-derived iPSCs by three different approaches. TALEN and CRISPR were equally effective and had minimal effects on off-target mutagenesis when they were targeted to a unique sequence region. Our efficient and precise correction method using TALEN and CRISPR technologies should provide a framework for future ex vivo gene therapy using patient-specific human iPSCs.

## Experimental Procedures

### Generation of Integration-Free DMD iPSCs

DMD fibroblasts were derived from a DMD patient lacking exon 44 of the dystrophin gene after the subject provided written informed consent. The use of patient-derived samples and the genomic analysis were approved by the Ethics Committee of Kyoto University (no. 824 and no. G259, respectively). DMD fibroblasts were cultured in Dulbecco’s modified Eagle’s medium supplemented with 5% fetal bovine serum. To generate integration-free DMD iPSCs, we transfected 6 × 10^5^ DMD fibroblasts with three episomal vectors (pCXLE-hOCT3/4-shp53-F, pCXLE-hSK, and pCXLE-hUL) by Neon electroporation (1650 V, 10 ms, 3 pulses) as described previously (Okita et al., 2011). The iPSC colonies that emerged were picked up and plated onto 24-well plates with feeders on day 31 and then expanded.

To screen for iPSC clones that were negative for residual episomal vectors, iPSC pellets were lysed with 500 μl of lysis solution (200 μg ml^−1^ proteinase K) at 55°C for 3–16 hr. Genomic DNA was purified by phenol-chloroform extraction and ethanol precipitation, and then used for quantitative PCR analyses using the primers listed in Table S6. An episomal plasmid was used to determine the standard curve, and DMD-iPSC clones with fewer than 0.01 copies were deemed integration-free iPSC clones. The original DMD-iPSC #1 (clone ID: CiRA00111) and the corrected clones, including the IF clone IF^D28^ (clone ID: CiRA00111-IF-D28), ES clone ES^H19^ (clone ID: CiRA00111-ES-H19), TALEN-mediated knockin clone TKI^I15^ (clone ID: CiRA00111-TKI-I15), and CRISPR-mediated knockin clone CKI^C2^ (clone ID: CiRA00111-CKI-C2), will be available from the RIKEN BRC Cell Bank (cell no. HPS0383-HPS0387).

### Unique *k*-mer Sequence Database

To identify unique sequence regions and avoid repeated sequences in the human genome, we generated all possible combinations of small *k*-mer sequences (≤16 bp) by a custom Perl script. We then mapped the *k*-mer sequences onto the human genome (hg19) using Bowtie (Langmead et al., 2009), with no mismatch allowed. Only uniquely mapped *k*-mer sequences were pooled as the data set. To visually show the stack of unique *k*-mer sequences, the mapping data were converted to the BEDGRAPH format by genomeCoverageBed and then converted to TDF format by igvtools or to the bigWig format by bedGraphToBigWig. The unique k-mer sequence (Unik) database will be available on our website (https://apps.cira.kyoto-u.ac.jp/igeats).

### Transfection of TALEN and CRISPR into Human iPSCs

Target iPSCs were pretreated with a ROCK inhibitor (Y-27632; Sigma) at 10 μM for at least 1 hr before electroporation. The cells were washed with PBS and treated with CTK solution for 1–3 min at 37°C to remove feeders and then were washed with PBS twice. Next, the iPSCs were further dissociated into single cells by a 0.25% Trypsin solution for 5–8 min at 37°C and were neutralized with culture medium containing 10% fetal bovine serum. We electroporated 10 μg of nuclease-expressing plasmids (TALENs: 5 μg left and 5 μg right; CRISPR: 5 μg Cas9 and 5 μg sgRNA) and 5 μg donor plasmid (if applicable) into 1 × 10^6^ cells using a NEPA 21 electroporator (poring pulse: pulse voltage, 125 V; pulse width, 5 ms; pulse number, 2; Negagene). Cells were plated onto one well of a six-well plate with feeders in the presence of 10 μM Y-27632 for 1–2 days.

### Analysis of Indel Patterns by Deep Sequencing

The dystrophin gene target region was PCR amplified with barcoding primers (DMD-MiSeq-Rd1-fwd1 and DMD-MiSeq-Rd2-rev1) and then adaptor primers (Multiplex P5 fwd and Multiplex P7 rev) using a high-fidelity PCR enzyme. The resultant PCR products were gel purified and quantified by a Qubit 2.0 Fluorometer (Life Technologies) and the KAPA Library Quantification Kit for Illumina (KAPA Biosystems). Each DNA sample was adjusted to 2 nM and denatured by 0.2 N NaOH solution for 5 min. The samples were further diluted to 12 pM, mixed with 4 pM of PhiX spike-in DNA, and run on MiSeq using the MiSeq Reagent Kit v2 for 2 × 150 bp sequencing. The generated FASTQ sequence files were filtered by the fastq_quality_filter program from the FASTX-Toolkit to remove low-quality sequencing reads. After removal of the PhiX sequences, the remaining sequencing reads were split based on the barcode indices by the fastx_barcode_splitter program. The resultant reads were mapped to the target sequences by BWA, and the mutation patterns were extracted from the CIGAR code and MD tag.

### Frameshift Screening without a Template Donor

Genomic DNAs from the transfected iPSCs were analyzed by the T7EI assay and restriction enzyme (XcmI) sensitivity assay to monitor the efficiency of the nuclease-mediated mutagenesis. Then the cells were dissociated into single cells and diluted to 200–500 cells per 10 cm dish with feeders. The subclonal colonies that emerged were picked on days 11–13 after reseeding. From the genomic PCR sequencing, the indels at dystrophin exon 45 with the (3*n* + 2) bp deletion or (3*n* + 1) bp insertion (where *n* is a nonnegative integer) were further expanded for later experiments.

### TALEN- or CRISPR-Mediated Exon 44 Knockin

For the knockin experiment, 5 μg of donor vector was cotransfected with TALEN expression vectors (5 μg for left TALEN and 5 μg for right TALEN) or Cas9 and sgRNA expression vectors (5 μg for Cas9 and 5 μg for sgRNA) using NEPA 21 as described above. Hygromycin B (25 μg ml^−1^; Invitrogen) selection was applied after iPSC colonies were recovered (4–5 days after transfection). The resulting hygromycin-resistant colonies were dissociated into single cells and plated at 200–500 cells per 10 cm dish with feeders. Each subclone was screened by genomic PCR (with P1-P2 primer pairs, amplifying a fragment from upstream of the 3′ arm to the EF1α-promoter, and P3-P4 primer pairs, amplifying a fragment from exon 44 to downstream of the 5′ arm). Homologous recombinants were further verified by Southern blot analysis using EcoRI and probe intron 45. After establishing the single-copy knockin clones, we electroporated the cells with 10 μg of the *Cre* expression vector pCXW-Cre-puro using NEPA 21. Clone isolation was carried out as described above, and excision of the hygromycin-selection cassette was confirmed by PCR screening with primers P1 and P4 and Southern blot analysis with EcoRI digestion and probe intron 45.

### Skeletal Muscle Differentiation by Dox-Inducible MYOD1

The induction of skeletal muscle differentiation from iPSCs was described previously (Tanaka et al., 2013). Briefly, a Dox-inducible MYOD1-expressing *piggyBac* vector, PB-TetO-MyoD, was coelectroporated with the *piggyBac* transposase vector PBaseII (Matsui et al., 2014) using NEPA 21 (125 V, 5 ms). G418 (Calbiochem) selection (100 μg ml^−1^) was applied to select stable PB-TetO-MyoD clones. Among the several G418-resistant clones, we screened for clones with a high mCherry induction rate upon addition of 1 μg ml^−1^ doxycycline (Funakoshi). Successful differentiation was confirmed by a spindle-shape-like morphology and immunocytochemical staining with myosin heavy chain (MHC) and α-skeletal muscle actin (α-SMA) antibodies on day 9 postdifferentiation.

## Author Contributions

H.L.L., S.Y., and A.H. conceived and designed the project. H.L.L., N.F., N.S., S.S., T.O., N.A, A.W., and A.H. performed the experiments. T.S. and T.Y. provided the TALEN construction platforms. M.T. constructed the website. H.S. provided the skeletal muscle differentiation protocol. H.L.L. and A.H. interpreted the data and wrote the manuscript.

## Accession Numbers

The plasmid DNAs used in this study are available from Addgene (https://www.addgene.org/) under accession numbers 60599–60605. DMD-patient-derived iPSCs and genetically corrected subclones are available from the RIKEN BRC Cell Bank (http://www.brc.riken.jp/lab/cell/english/) under accession numbers HPS0383–HPS0387.

## Supplemental Information

Document S1. Supplemental Experimental Procedures, Figures S1–S6, and Tables S1–S6

Document S2. Article plus Supplemental Information
