# Supplementary material for: Precise Correction of the Dystrophin Gene in Duchenne Muscular Dystrophy Patient Induced Pluripotent Stem Cells by TALEN and CRISPR-Cas9
Source: Stem Cell Reports. 2014 Nov 26;4(1):143–54. doi: 10.1016/j.stemcr.2014.10.013 (PMC4297888; doi:10.1016/j.stemcr.2014.10.013)
Supplement: Document S1. Supplemental Experimental Procedures, Figures S1–S6, and Tables S1–S6 [file mmc1.pdf]

Stem Cell Reports, Volume *4*

Supplemental Information

Precise Correction of the Dystrophin Gene in Duchenne Muscular Dystrophy Patient Induced Pluripotent Stem Cells by TALEN and CRISPR-Cas9

Hongmei Lisa Li, Naoko Fujimoto, Noriko Sasakawa, Saya Shirai, Tokiko Ohkame, Tetsushi Sakuma, Michihiro Tanaka, Naoki Amano, Akira Watanabe, Hidetoshi Sakurai, Takashi Yamamoto, Shinya Yamanaka, and Akitsu Hotta

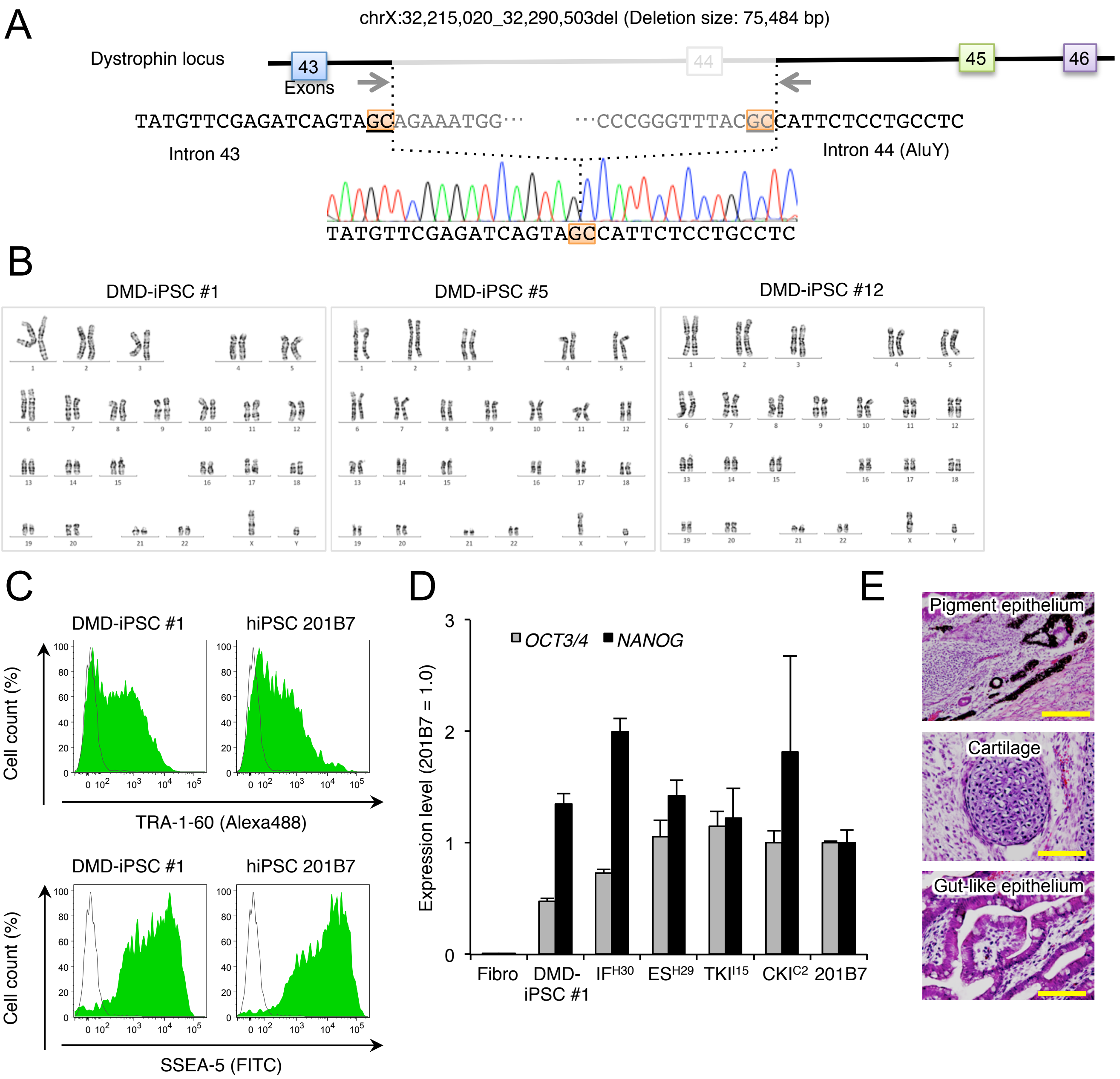

Figure S1. Establishment of DMD-iPSCs, Related to Figures 2-6.

(A) Breakpoint analysis of the copy number loss of exon 44 in the dystrophin gene in the genome of a DMD patient who lacked exon 44. Multiple PCRs and Sanger sequences identified the loss of a 75 kbp region including exon 44. The orange box in the lower panel indicates the micro-homology sequence "GC" found at the breakpoint. (B) iPS cell lines generated by episomal vectors maintained normal karyotypes. (C) Flow cytometry analysis showed that DMD-iPSC clone #1 was positive for pluripotency markers TRA-1-60 and SSEA-5, equivalent to the control hiPSC 201B7 clone. (D) qRT-PCR analysis for *OCT3/4* and *NANOG* in the DMD-iPSC #1 clone and the derivative clones. The data were normalized to those of the control hiPSC line, 201B7. The values represent mean ± SD among technical replicates (n = 3). (E) A histological examination of a teratoma from DMD-iPSC #1 showed the formation of tissue structures that correspond to the three germ layers: pigmented epithelium (ectoderm), cartilage (mesoderm) and gut-like epithelium (endoderm). Scale bar, 100 μm.

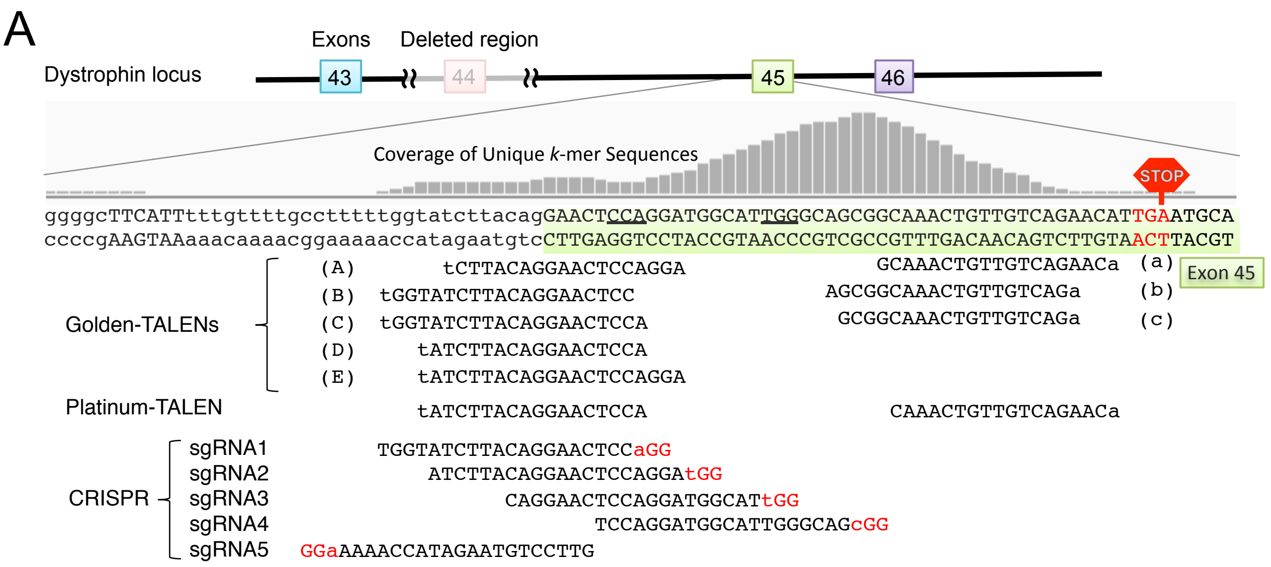

B

SSA activity: Low – High

L \ R	–	a	b	c	HPRT1
–	0.00	1.01	0.89	0.98	1.02
A	1.25	8.06	8.06	5.62	–
B	1.18	6.19	8.86	8.86	–
C	1.11	2.53	4.18	3.73	–
D	1.28	2.64	6.79	5.29	–
E	1.27	14.70	2.98	14.07	–
HPRT1	0.91	–	–	–	15.09

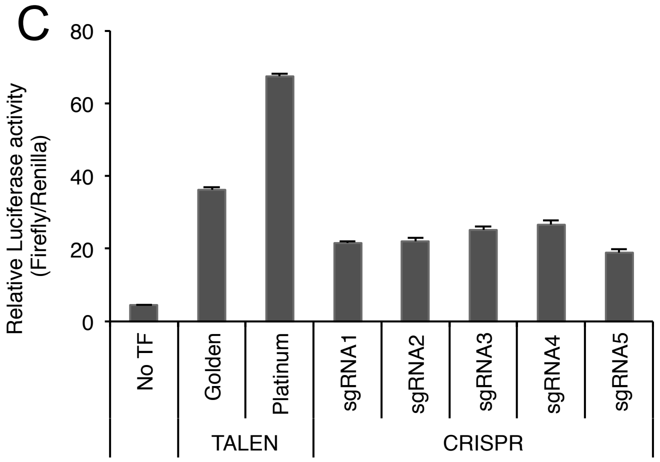

Figure S2. Screening for the most active TALENs and CRIPSR-sgRNAs, Related to Figure 2.

(A) The target sites of the nucleases used in this study, including TALEN pairs and CRISPR-sgRNAs, at exon 45 of the dystrophin gene. Golden-TALENs were constructed with the Golden-Gate assembly method, and Platinum-TALEN was constructed with the Platinum-Gate assembly method with some non-RVD variation. CRISPR-sgRNA1-4 target the sense strand, whereas sgRNA5 targets the antisense strand. (B) The activities of Golden-TALENs were analyzed by a SSA (single-strand annealing) assay in HEK293T cells (n = 3). Left TALENs (A-E) and right TALENs (a-c) were co-transfected with a luciferase-expressing vector, where the luciferase gene was separated from the exon 45 target site. Among the pairs of combinations tested, the E/a and E/c pairs showed highest SSA activity, a result similar to that of the positive control TALEN pair targeting the HPRT1 gene. (C) The SSA activities of CRISPR-sgRNAs in HEK293T cells. Golden-TALEN (E/a pair) and the Platinum-TALEN pair were used as positive controls. Each of the five CRISPR-sgRNAs was transfected with human-codon optimized wild-type Cas9. The values represent mean ± SD among technical replicates (n = 3).

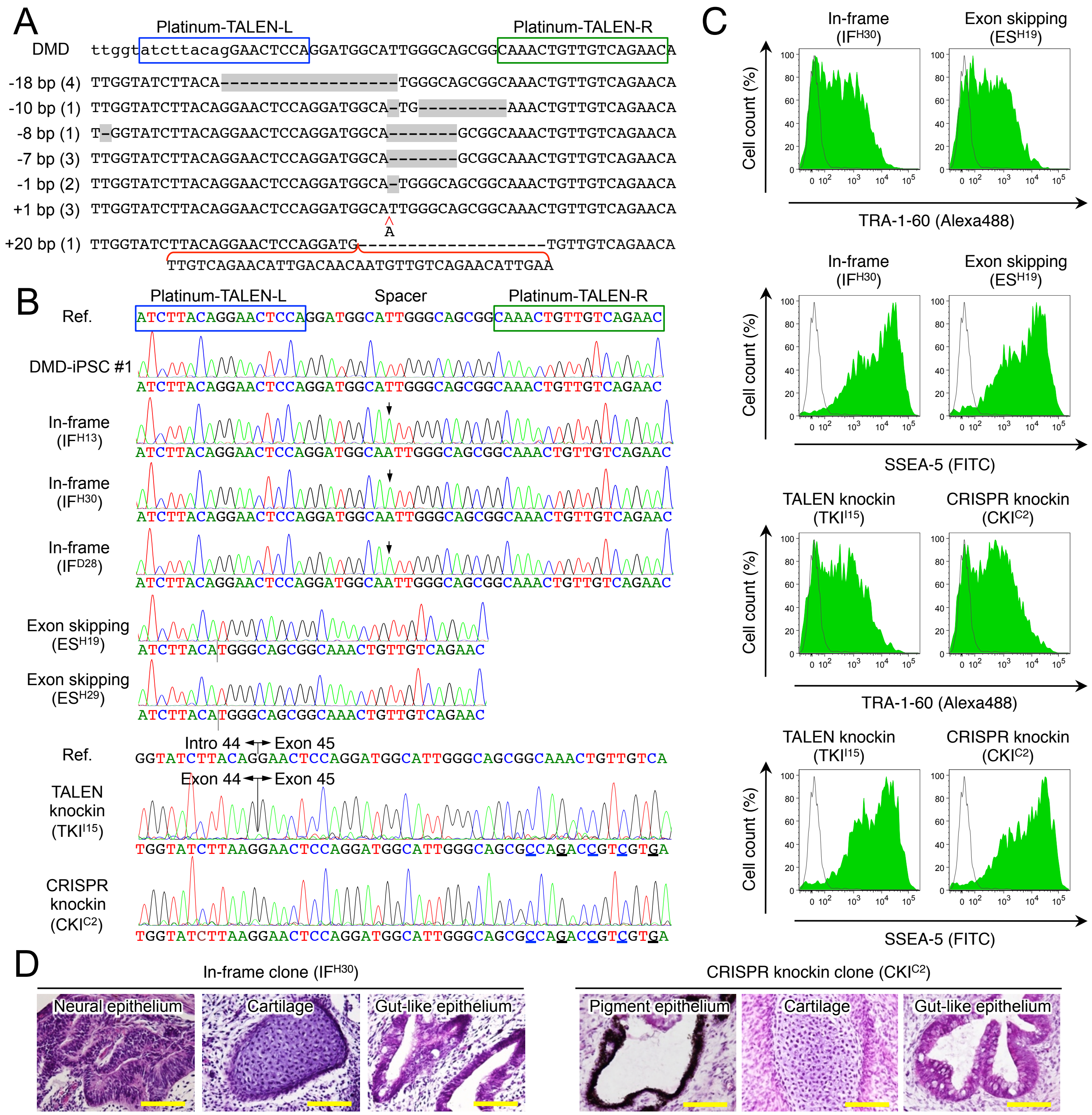

Figure S3. Confirmation of the corrected DMD-iPS cell clones, Related to Figures 3, 4.

(A) Indel patterns induced by Platinum-TALEN. The sizes of the indels are shown on the left, and the number of clones for each indel are indicated in parentheses. Clones with a 1 bp insertion are in-frame clones, and those with the 18 bp deletion are exon 45 skipping clones. Other indels did not restore the reading frame. (B) Sanger sequence electrograms confirmed corresponding clones with the 1 bp insertion (IF^{H13}, IF^{H30} and IF^{D28}), exon skipping clones with the 18 bp deletion (ES^{H19} and ES^{H29}) and knockin clones with the genomic exon 44-45 junction (TKI^{I15} and CKI^{C2}). The original DMD-iPSC sequence is shown at the top and middle for reference. Black arrow indicates the 1 bp insertion. Underlined sequence in knockin clones indicates 5 silent mutations to prevent the re-cutting. (C) The expression of pluripotency markers, TRA-1-60 and SSEA-5, in the in-frame, exon skipping and exon 44 knockin clones as assessed by flow cytometry. (D) Teratoma assay of the corrected DMD-iPS clones (IF^{H30} and CKI^{C2}) showed the formation of tissue structures that correspond to the three germ layers. Scale bar, 100 µm.

A

		Position	Sequence
On-target	Platinum-TALEN-Left	chrX:31986626-31986642	tATCTTACAGGAACTCCA
	sgRNA1	chrX:31986622-31986644	TGGTATCTTACAGGAACTCCAGG
Off-target	OT1	chr14:52643730-52643776	TGGTATCTTAaAGaAACTCCAGG
	OT2	chr9:33384854-33384900	TGGgtTCTTACAGGAgCTCCAGG
	OT3	chr12:91808790-91808836	TGGTcTCTTACAGGAACTCaAGa
	OT4	chr4:100047228-100047274	aGGTATCTTAgAGGAACTCCAtG
	OT5	chr2:66263917-66263963	TGGgATCTaACAGtAACTCCAGG

C

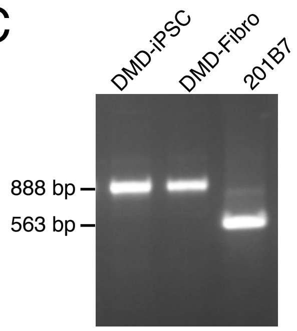

B

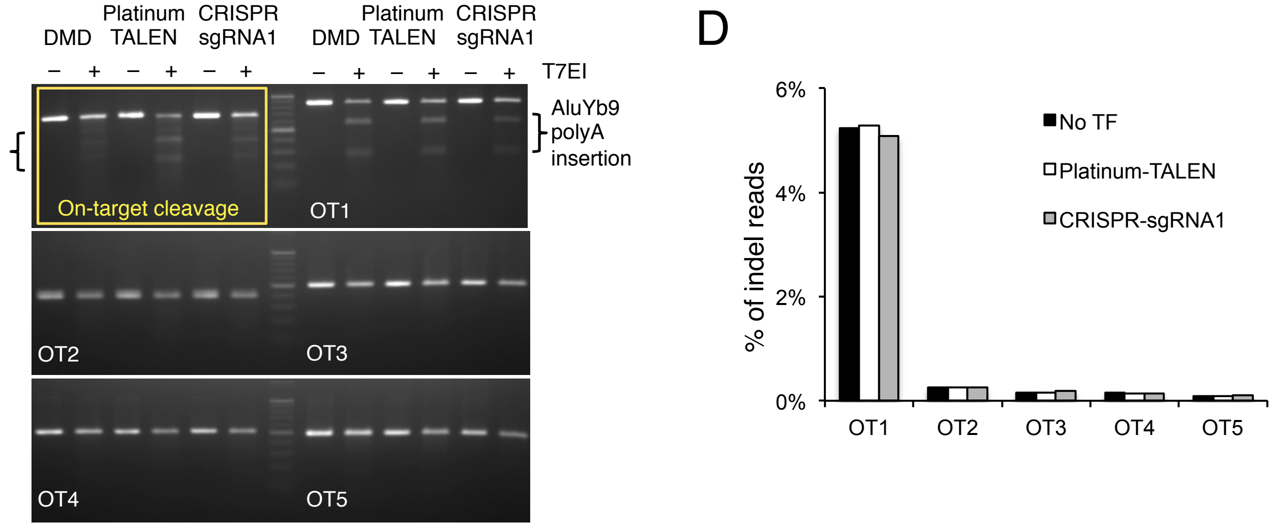

Figure S4. No severe mutagenesis observed at the predicted off-target sites, Related to Figure 5.

(A) The predicted off-target (OT) sites allowed up to three mismatches for CRISPR-sgRNA1. Red characters indicate mismatches compared to on-target sgRNA1. (B) The predicted off-target loci were amplified by PCR and analyzed by a T7EI assay. The yellow box shows successful detection of the on-target mutagenesis by our T7EI assay. OT2-5 sites showed no cleavage at the off-target sites except for OT1. Because the PCR amplicon size for OT1 was larger than expected and because we observed cleaved bands even in the control (No TF), we sequenced the OT1 PCR fragment and found the homozygous insertion of AluYb9 in the middle of the OT1 site. (C) Genomic PCR analysis confirmed the insertion of the AluYb9 at OT1 in original fibroblasts derived from the DMD patient, but not in a control human iPSC clone (201B7). (D) Indel mutations at the off-target sites were assessed by amplicon deep sequencing. The OT1 site suffered from a high indel ratio due to the poly A region of the AluYb9 element. No TF: no transfection DMD-iPSCs.

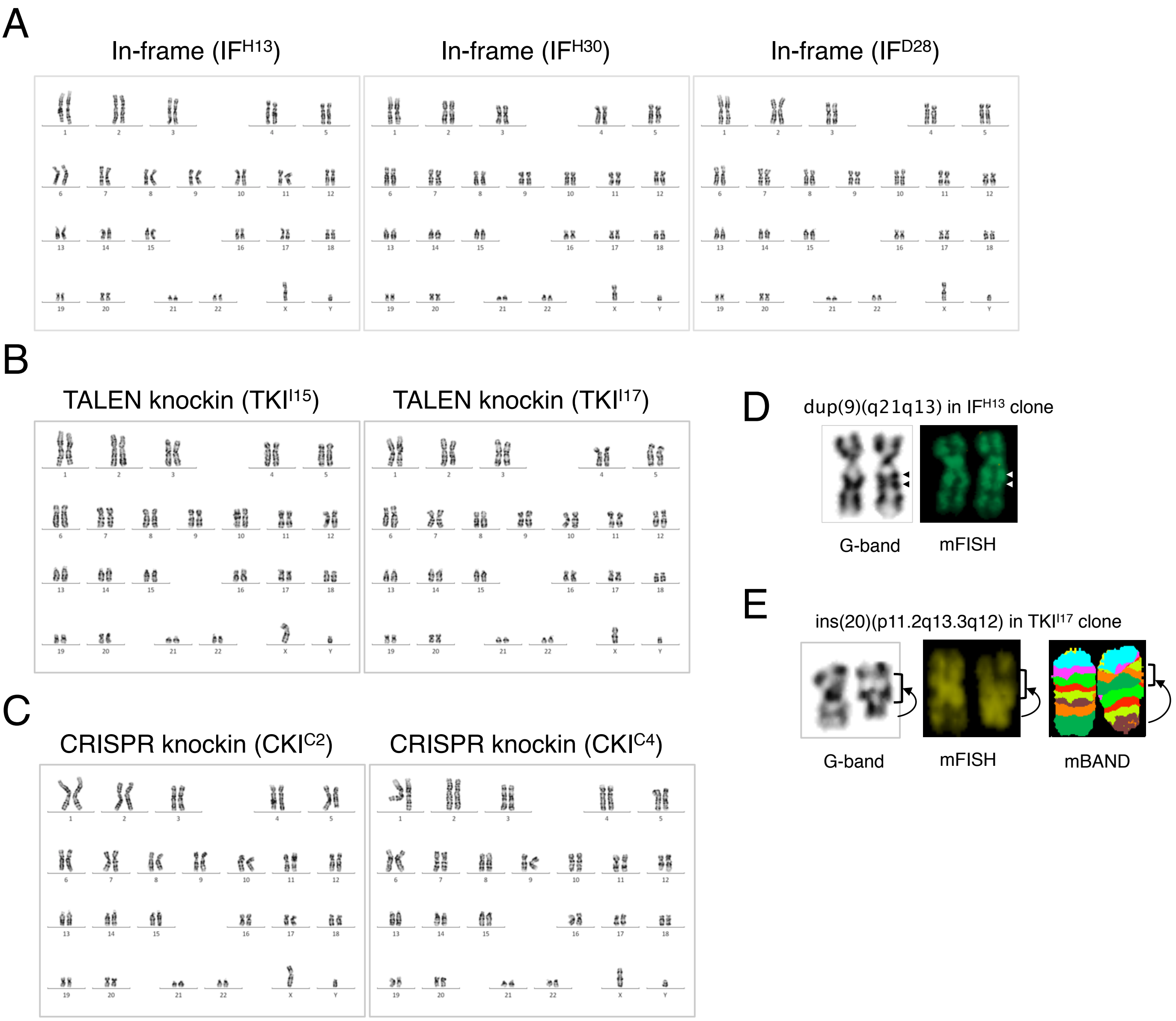

Figure S5. Karyotyping analysis of the corrected clones, Related to Figure 5A.

(A) (B) and (C) High-resolution G-banding analysis revealed that most cells showed a normal karyotype with corrected clones, in-frame clones (IF^{H13}, IF^{H30} and IF^{D28}) (A), TALEN-mediated knockin clones (TKI^{I15} and TKI^{I17}) (B) and CRISPR-mediated knockin clones (CKI^{C2} and CKI^{C4}) (C). (D) G-banding and mFISH images showed suspected duplication (two arrowheads) at chromosome 9 in the IF^{H13} clone (5 metaphases per 23 examined). (E) mFISH and mBAND showed an inverted insertion of chromosome 20 (ins(20)(p11.2q13.3q12)) in TALEN-mediated knockin clone TKI^{I17}.

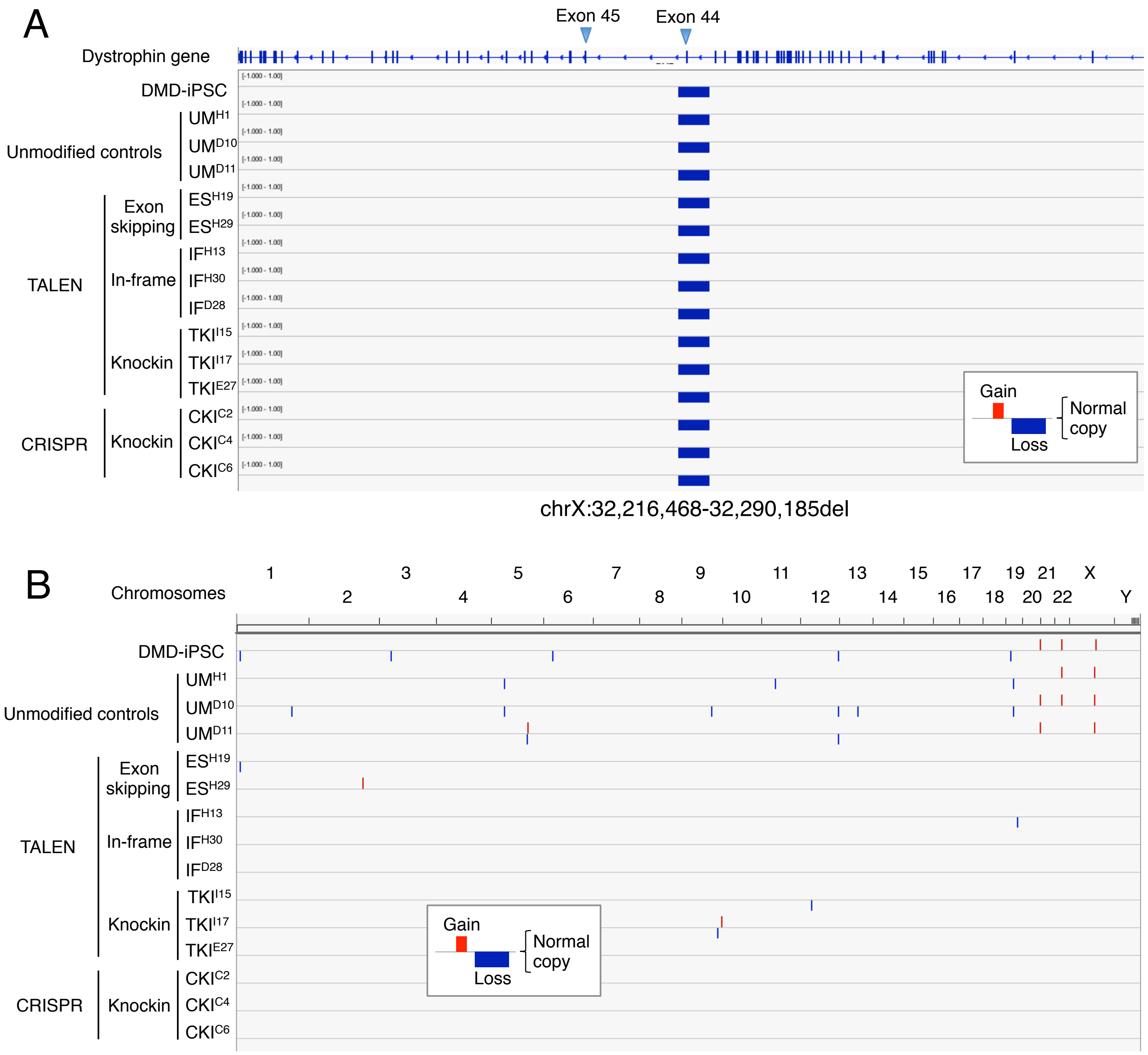

Figure S6. Detection of CNVs in the dystrophin gene and in the corrected clones, Related to Figure 5B.

(A) The copy numbers detected by the SNP array and PennCNV are plotted. The grey horizontal axis indicates normal copies (i.e. two copies for autosomal chromosomes and one copy for X and Y chromosomes). The blue bar under the grey axis indicates the copy number loss at the exon 44 region in the dystrophin gene. We successfully identified the deletion of chrX:32,216,468-32,290,185 in all DMD samples, which is in close agreement with break point analysis (chrX: 32,215,020-32,290,503del), as shown in Supplementary Fig. 1a. (B) De novo CNVs found in genetically modified clones. Red bars above the grey axis indicate copy number gain, and blue bars below the grey axis indicate copy number loss.

Table S1. Summary of unique *k*-mers to identify unique regions in the genome, Related to Figure 1.

Length	Total No. of *k*-mers		No. of unique *k*-mers	Coverage of the human genome
16-mer	4^{16}	4,294,967,296	980,117,801	62.60%
15-mer	4^{15}	1,073,741,824	263,051,237	27.60%
14-mer	4^{14}	268,435,456	55,041,124	7.29%
13-mer	4^{13}	67,108,864	5,842,203	1.13%
12-mer	4^{12}	16,777,216	324,003	0.08%
11-mer	4^{11}	4,194,304	3,097	9.61 x 10^{-4} %
10-mer	4^{10}	1,048,576	1	2.82 x 10^{-7} %

Table S2. Examples of the microhomology-mediated deletion patterns, Related to Figure 3.

Nucleases	Target sequences	Microhomology motif	Deletion size (bp)
Golden-TALEN	TTTTGCCTTTTTGGTATCTTACAGGAACTCCAGGATGGCATTGGGCAGCGGCAAACTGTTGTCAGAAC	GGCA	Δ 6
	TTTTGCCTTTTTGGTATCTTACAGGAACTCCAGGATGGCATTGGGCAGCGGCAAACTGTTGTCAGAAC	GGCA	Δ 7
	TTTTGCCTTTTTGGTATCTTACAGGAACTCCAGGATGGCATTGGGCAGCGGCAAACTGTTGTCAGAAC	GGCA	Δ 13
	TTTTGCCTTTTTGGTATCTTACAGGAACTCCAGGATGGCATTGGGCAGCGGCAAACTGTTGTCAGAAC	TGG	Δ 6
	TTTTGCCTTTTTGGTATCTTACAGGAACTCCAGGATGGCATTGGGCAGCGGCAAACTGTTGTCAGAAC	CAG	Δ 15
	TTTTGCCTTTTTGGTATCTTACAGGAACTCCAGGATGGCATTGGGCAGCGGCAAACTGTTGTCAGAAC	TTG	Δ 18
Platinum-TALEN	TTTTGCCTTTTTGGTATCTTACAGGAACTCCAGGATGGCATTGGGCAGCGGCAAACTGTTGTCAGAAC	GGCA	Δ 6
	TTTTGCCTTTTTGGTATCTTACAGGAACTCCAGGATGGCATTGGGCAGCGGCAAACTGTTGTCAGAAC	GGCA	Δ 7
	TTTTGCCTTTTTGGTATCTTACAGGAACTCCAGGATGGCATTGGGCAGCGGCAAACTGTTGTCAGAAC	GGCA	Δ 13
	TTTTGCCTTTTTGGTATCTTACAGGAACTCCAGGATGGCATTGGGCAGCGGCAAACTGTTGTCAGAAC	TGG	Δ 6
	TTTTGCCTTTTTGGTATCTTACAGGAACTCCAGGATGGCATTGGGCAGCGGCAAACTGTTGTCAGAAC	CAG	Δ 15
	TTTTGCCTTTTTGGTATCTTACAGGAACTCCAGGATGGCATTGGGCAGCGGCAAACTGTTGTCAGAAC	TTG	Δ 18
CRISPR-sgRNA1	TTTTGCCTTTTTGGTATCTTACAGGAACTCCAGGATGGCATTGGGCAGCGGCAAACTGTTGTCAGAAC	CAGGA	Δ 9
CRISPR-sgRNA2	TTTTGCCTTTTTGGTATCTTACAGGAACTCCAGGATGGCATTGGGCAGCGGCAAACTGTTGTCAGAAC	CAGGA	Δ 9
CRISPR-sgRNA3	TTTTGCCTTTTTGGTATCTTACAGGAACTCCAGGATGGCATTGGGCAGCGGCAAACTGTTGTCAGAAC	GGCA	Δ 6
	TTTTGCCTTTTTGGTATCTTACAGGAACTCCAGGATGGCATTGGGCAGCGGCAAACTGTTGTCAGAAC	TGG	Δ 6
	TTTTGCCTTTTTGGTATCTTACAGGAACTCCAGGATGGCATTGGGCAGCGGCAAACTGTTGTCAGAAC	CAG	Δ 15
CRISPR-sgRNA4	TTTTGCCTTTTTGGTATCTTACAGGAACTCCAGGATGGCATTGGGCAGCGGCAAACTGTTGTCAGAAC	GGCA	Δ 6
	TTTTGCCTTTTTGGTATCTTACAGGAACTCCAGGATGGCATTGGGCAGCGGCAAACTGTTGTCAGAAC	GGCA	Δ 7
	TTTTGCCTTTTTGGTATCTTACAGGAACTCCAGGATGGCATTGGGCAGCGGCAAACTGTTGTCAGAAC	GGCA	Δ 13
CRISPR-sgRNA5	TTTTGCCTTTTTGGTATCTTACAGGAACTCCAGGATGGCATTGGGCAGCGGCAAACTGTTGTCAGAAC	TTTTG	Δ 8
	TTTTGCCTTTTTGGTATCTTACAGGAACTCCAGGATGGCATTGGGCAGCGGCAAACTGTTGTCAGAAC	CTT	Δ 11

Red characters: microhomology sequences with more than 3 bp that flank the cleavage site of TALENs or CRISPRs.
Underline: the recognition site for TALEN pairs or CRISPR-sgRNAs
v: the cleavage sites of CRISPR-Cas9.

Table S3. Summary of capture statistics for the exome sequencing, Related to Figure 5C.

Category		Sample ID	Total Reads	Remove identical reads*	Unique %	Mapped reads†	Mapping %
Original fibroblast		DMD_fibroblast	129,536,708	88,652,535	68%	86,671,306	97.8%
Original iPSC clone		DMD-iPSC	130,243,436	106,064,367	81%	104,115,285	98.2%
Unmodified controls		UM^{D10}	134,171,856	90,013,937	67%	87,747,006	97.5%
		UM^{D11}	138,880,388	114,021,013	82%	112,671,565	98.8%
		UM^{H1}	126,398,760	109,834,280	87%	107,919,254	98.3%
TALEN	Exon skipping	ES^{H19}	136,945,384	104,830,953	77%	102,317,225	97.6%
		ES^{H29}	122,965,492	107,018,775	87%	104,802,925	97.9%
	In-frame	IF^{H13}	119,170,318	94,297,902	79%	92,796,375	98.4%
		IF^{H30}	138,612,424	109,896,988	79%	108,131,267	98.4%
		IF^{D28}	131,120,892	108,044,479	82%	106,164,805	98.3%
	Knockin	TKI^{I15}	95,030,774	80,900,233	85%	79,835,405	98.7%
		TKI^{I17}	97,704,786	82,501,149	84%	81,278,499	98.5%
		TKI^{E27}	92,361,384	78,223,262	85%	77,118,236	98.6%
CRISPR	Knockin	CKI^{C2}	119,453,700	107,111,909	90%	105,433,227	98.4%
		CKI^{C4}	130,291,792	113,680,521	87%	112,100,032	98.6%
		CKI^{C6}	125,818,120	111,141,505	88%	109,767,667	98.8%

* Duplicated identical reads were deemed as PCR artifact during the sample preparation of the sequencing.
† Mapped to human genome (hg19)

Table S4. SNVs detected by the exome sequencing, Related to Figure 5C.

UM[H1]	UM[D10]	UM[D11]	ES[H19]	ES[H29]	IF[H13]	IF[H30]	IF[D28]	TKI[I15]	TKI[I17]	TKI[E27]	CKI[C2]	CKI[C4]	CKI[C6]	chr	Position	Gene	ExonicFunc	ref	alt
									SNV					chr1	10725468	*CASZ1*	synonymous SNV	C	T
SNV									SNV					chr1	26671594	*AIM1L*	nonsynonymous SNV	T	C
SNV									SNV					chr1	26671595	*AIM1L*	synonymous SNV	G	A
SNV							SNV							chr1	47685584	*TAL1*	synonymous SNV	T	C
		SNV												chr1	47685593	*TAL1*	synonymous SNV	A	C
								SNV						chr1	152084216	*TCHH*	nonsynonymous SNV	C	G
					SNV									chr1	152084437	*TCHH*	nonsynonymous SNV	A	T
					SNV									chr1	152084438	*TCHH*	nonsynonymous SNV	G	C
						SNV			SNV					chr1	152681689	*LCE4A*	synonymous SNV	G	C
											SNV			chr1	228335240	*GUK1*	nonsynonymous SNV	G	A
					SNV	SNV								chr2	31457663	*EHD3*	nonsynonymous SNV	C	T
								SNV						chr2	204306002	*RAPH1*	synonymous SNV	A	G
								SNV						chr2	204306008	*RAPH1*	synonymous SNV	A	G
								SNV	SNV					chr3	45801382	*SLC6A20*	synonymous SNV	C	T
	SNV													chr3	75786748	*ZNF717*	nonsynonymous SNV	C	T
	SNV													chr3	167217960	*WDR49*	synonymous SNV	C	T
	SNV													chr3	167217964	*WDR49*	nonsynonymous SNV	A	G
				SNV										chr3	195506245	*MUC4*	nonsynonymous SNV	C	A
				SNV										chr3	195506645	*MUC4*	nonsynonymous SNV	G	A
											SNV			chr3	195515290	*MUC4*	nonsynonymous SNV	C	G
SNV														chr3	197500320	*FYTTD1*	nonsynonymous SNV	C	A
											SNV			chr4	88537180	*DSPP*	synonymous SNV	T	C
										SNV				chr5	140531592	*PCDHB6*	nonsynonymous SNV	C	T
									SNV					chr5	141974916	*FGF1*	nonsynonymous SNV	G	T
									SNV					chr6	16327915	*ATXN1*	nonsynonymous SNV	A	C
											SNV	SNV		chr6	25850031	*SLC17A3*	NA	A	G
SNV				SNV	SNV				SNV				SNV	chr6	57393112	*PRIM2*	NA	T	C
	SNV													chr6	57398264	*PRIM2*	synonymous SNV	A	G
													SNV	chr7	5413826	*TNRC18*	nonsynonymous SNV	T	G
										SNV				chr7	56087399	*PSPH*	nonsynonymous SNV	G	A
	SNV													chr7	89937168	*C7orf63*	synonymous SNV	G	A
										SNV				chr7	100639153	*MUC12*	nonsynonymous SNV	C	T
									SNV					chr8	101718965	*PABPC1*	nonsynonymous SNV	G	A
									SNV					chr8	101718968	*PABPC1*	nonsynonymous SNV	C	T
											SNV			chr8	101719004	*PABPC1*	nonsynonymous SNV	G	A
					SNV									chr8	133051259	*OC90*	nonsynonymous SNV	C	A
							SNV							chr8	144995598	*PLEC*	synonymous SNV	G	A
								SNV						chr9	35906583	*HRCT1*	nonsynonymous SNV	T	A
									SNV					chr9	124914613	*NDUFA8*	synonymous SNV	C	T
									SNV					chr9	125143792	*PTGS1*	synonymous SNV	C	A
					SNV	SNV								chr9	129455586	*LMX1B*	nonsynonymous SNV	C	T
								SNV		SNV				chr11	1090928	*MUC2*	nonsynonymous SNV	A	C
											SNV	SNV	SNV	chr11	62652670	*SLC3A2*	synonymous SNV	C	T
SNV														chr12	4737745	*AKAP3*	nonsynonymous SNV	C	A
					SNV	SNV								chr12	120117794	*PRKAB1*	synonymous SNV	G	A
SNV														chr12	122255756	*SETD1B*	nonsynonymous SNV	G	C
SNV														chr12	130921739	*RIMBP2*	nonsynonymous SNV	G	A
SNV														chr13	20797129	*GJB6*	nonsynonymous SNV	G	A
								SNV						chr14	70924501	*ADAM21*	nonsynonymous SNV	C	G
									SNV					chr14	104145852	*KLC1*	synonymous SNV	G	A
								SNV	SNV					chr15	43700161	*TP53BP1*	nonsynonymous SNV	T	C
				SNV										chr15	65678318	*IGDCC4*	nonsynonymous SNV	T	G
				SNV				SNV						chr16	1307050	*TPSD1*	synonymous SNV	C	T
				SNV				SNV						chr16	1307060	*TPSD1*	nonsynonymous SNV	A	G
										SNV				chr16	3026805	*PKMYT1*	nonsynonymous SNV	G	A
					SNV									chr16	66788886	*CCDC79*	nonsynonymous SNV	G	T
								SNV	SNV					chr17	3638150	*ITGAE*	nonsynonymous SNV	C	A
					SNV	SNV								chr17	9503461	*WDR16*	synonymous SNV	G	T
								SNV						chr17	21318698	*KCNJ12,KCNJ18*	nonsynonymous SNV	C	T
											SNV	SNV	SNV	chr17	66036857	*KPNA2*	nonsynonymous SNV	C	A
							SNV							chr19	1430261	*DAZAP1*	synonymous SNV	G	C
				SNV										chr19	5789565	*DUS3L*	nonsynonymous SNV	T	C
										SNV				chr19	7935879	*FLJ22184*	synonymous SNV	G	A
													SNV	chr19	35843086	*FFAR1*	nonsynonymous SNV	G	A
												SNV		chr19	40392347	*FCGBP*	nonsynonymous SNV	C	G
											SNV		SNV	chr19	40392360	*FCGBP*	nonsynonymous SNV	G	A
				SNV										chr19	27251	*KIR2DL2*	nonsynonymous SNV	C	T
	SNV													chrX	114425196	*RBMXL3*	nonsynonymous SNV	G	A
	SNV													chrX	114425210	*RBMXL3*	synonymous SNV	G	A
									SNV					chrX	118603830	*SLC25A5*	synonymous SNV	A	G
									SNV					chrX	118603844	*SLC25A5*	nonsynonymous SNV	T	G
9	7	1	0	8	9	5	3	12	17	6	8	4	6						

Total No. of detected SNVs per each clone.

Table S5. Indels detected by the exome sequencing, Related to Figure 5C.

UM^{H1}	UM^{D10}	UM^{D11}	ES^{H19}	ES^{H29}	IF^{H13}	IF^{H30}	IF^{D28}	TKI^{I15}	TKI^{I17}	TKI^{E27}	CKI^{C2}	CKI^{C4}	CKI^{C6}	chr	start	ref	alt	Gene	Comment
				Indel					Indel			Indel		chr12	7045891	–	CAGCAGCAG	*ATN1*	Triple repeat
						Indel								chr12	7045891	–	CAGCAGCAGCAG	*ATN1*	Triple repeat
													Indel	chr19	36002421	–	CTGCTGCCA	*DMKN*	Triple repeat
												Indel	Indel	chr19	36002421	–	CTGCTGCTG	*DMKN*	Triple repeat
											Indel	Indel		chr20	60891763	C	–	*LAMA5*	
		Indel	Indel		Indel	Indel			Indel	Indel	Indel			chr22	29885581	24 bp	–	*NEFH*	
											Indel	Indel		chr22	41573513	AG	–	*EP300*	
					Indel	Indel	Indel							chrX	31986615	–	T	*DMD*	On-target
			Indel	Indel										chrX	31986615	18 bp	–	*DMD*	On-target
0	0	1	1	1	1	2	0	0	2	1	3	4	2						

Total No. of detected Indels per clone.

```
24 bp: AGGCCAAGTCCCCAGAGAAGGAAG
18 bp: ATGCCATCCTGGAGTTCC
```

Table S6. List of primers used, Related to the Experimental Procedures.

Targets	Primers	Sequence
qRT-PCR for pluripotency markers	hACTB-Fwd	CCAACCGCGAGAAGATGA
	hACTB-Rev	CCAGAGGCGTACAGGGATAG
	hOCT3/4-Fw	GACAGGGGGAGGGGAGGAGCTAGG
	hOCT3/4-Rv	CTTCCCTCCAACCAGTTGCCCCAAAC
	hNANOG-Fw	TGAACCTCAGCTACAAACAG
	hNANOG-Rv	TGGTGGTAGGAAGAGTAAAG
Construction of sgRNAs	DMD-sgRNA1-fwd	GAGACCACTTGGATCCGggtatcttacaggaactccGTTTTAGAGCTAGAAATAGCA
	DMD-sgRNA2-fwd	GAGACCACTTGGATCCGtcttacaggaactccaggaGTTTTAGAGCTAGAAATAGCA
	DMD-sgRNA3-fwd	GAGACCACTTGGATCCGaggaactccaggatggcatGTTTTAGAGCTAGAAATAGCA
	DMD-sgRNA4-fwd	GAGACCACTTGGATCCGCCAGGATGGCATTGGGCAGGTTTTAGAGCTAGAAATAGCA
	DMD-sgRNA5-fwd	GAGACCACTTGGATCCGTTCCTGTAAGATACCAAAAGTTTTAGAGCTAGAAATAGCA
	sgRNA-Universal-rev	GCCCGGGTTTGAATTCAAAAAAAGCACCGACTCGGTGCCACTTTTTCAAGTTGATAA CGGACTAGCCTTATTTTAACTTGCTATTTCTAGCTCTAA
Construction of Donor template	DMD-Donor-F1_FW	ACCGCGGTGGCGGCCGCTTGCTATTGTGTCAAGG
	DMD-Donor- F1_RV_2	GCTCGCCGGCAAAACAAAAATGAAGCC
	DMD-Donor-F2_FW_2	GTTTTGCCGGCGAGCTCAGACGATAA
	DMD-Donor-F2_RV_2	GTAAAACATCGCGCAACGCAATTAGT
	DMD-Donor-F3_FW_2	TGCGCGATGTTTTACATAATCCATCTATTTT
	DMD-Donor-F3_RV	GGAGTTCCTTAAGATACCATTTGTATTTAG
	DMD-Donor-F4_FW_2	GCCAGACCGTCGTGAGAACATTGAATGCAACTGG
	DMD-Donor-F4_RV	CGGTACCCGGGGATCCAAGAGCTTGGCAAAAGAAC
Sanger sequencing for dystrophin exon 45	DMD-screening-fwd	CACCTCTCGTATCCACGATCACTAAG
	DMD-screening-rev	TAGTGCCTTTCACCCTGCTTAT
Deep sequencing for dystrophin exon 45	DMD-MiSeq-Rd1-fwd-X	CTCTTTCCCTACACGACGCTCTTCCGATCTXXXXAATAAAAAGACATGGGGCTTCA
	DMD-MiSeq-Rd2-rev-X	CTGGAGTTCAGACGTGTGCTCTTCCGATCTXXXXCTGGCATCTGTTTTTGAGGA
	Multiplex P5 fwd	AATGATACGGCGACCACCGAGATCTACACTCTTTCCCTACACGACGCTC
	Multiplex P7 rev	CAAGCAGAAGACGGCATACGAGATGTGACTGGAGTTCAGACGTGTGCTC
qPCR for residual episomal vectors	hFbx15-2F	GCCAGGAGGTCTTCGCTGTA
	hFbx15-2R	AATGCACGGCTAGGGTCAAA
	EBNA-183F	ATCAGGGCCAAGACATAGAGATG
	EBNA-243R	GCCAATGCAACTTGGACGTT
Screening knockin clones	P1	AGCTTTACCTCAGTCTCAGAAAACA
	P2	ACCCGTTGCGAAAAAGAACGTT
	P3	AGAATTGGGAACATGCTAAATACAA
	P4	TTGGATTAGATTGAGCCTAGTTCAG
	DMD-In45-F	CCCATGATTGCTTAAAGGTGA
	DMD-In45-R	AGAGCTTGGCAAAAGAACGA
Off-target analysis	gRNA-DMD1-OT1Fwd	TATATGGAAGCGTGGTCTGTGATAATT
	gRNA-DMD1-OT1Rev	TCCCTTTTATCAGCTAGGGCTCTTA
	gRNA-DMD1-OT2Fwd	GTCCAAGAACCGGCCAGAACTCAG
	gRNA-DMD1-OT2Rev	CCATCCATTCCCCAATAAAGCAAGGC
	gRNA-DMD1-OT3Fwd	AAAATGTTGAGAATTGCCTATTTGA
	gRNA-DMD1-OT3Rev	GATTCTTGCCAATTCTCTTTACACA
	gRNA-DMD1-OT4Fwd	CCTCCTCTCTTTTGTTGTATCTTCTCT
	gRNA-DMD1-OT4Rev	TTTCCATTTCACAAACCATTAACTC
	gRNA-DMD1-OT5Fwd	AGGGCTGATTATATTAGGAGAGTGG
	gRNA-DMD1-OT5Rev	AAAAATTTCCCATCTAGGAAGTGAG
cDNA analysis for dystrophin expression	exon 43	ACAAAGCTCAGGTCGGATTG
	exon 46	AGTTGCTGCTCTTTTCCAGGT

Supplemental Experimental Procedures

DNA construction

The expression plasmids of Golden-TALENs shown in Figure S2A were constructed using the Golden-Gate cloning method as described previously (Sakuma et al., 2013a). Platinum-TALEN expression plasmids were constructed using the Platinum-Gate system with non-RVD (repeat variable di-residue) variations (Sakuma et al., 2013b).

The Cas9 cDNA sequence (SphcCas9) from *Streptococcus pyogenes* was codon-optimized for human codon usage bias, and possible *cis*-acting motifs (i.e. splicing sites, poly A sites and destabilizing motifs) were removed by DNA synthesis (GenScript). The synthesized SphcCas9 cDNA was cloned into pHL-EF1α-GW-iP-A (Addgene 60599) by the Gateway LR reaction to construct the pHL-EF1α-SphcCas9-iP vector, which contained an EF1α promoter for high expression in human iPS cells. To construct custom sgRNA expression vectors, two oligos containing the sgRNA target site and a universal reverse primer (Table S6) were PCR amplified and cloned into the BamHI-EcoRI site of the pHL-H1-ccdB-mEF1α-RiH vector (Addgene 60601), which used the H1 promoter to drive sgRNA. The resultant sgRNA expression vectors pHL-H1-sgRNA[DMD1~3]-mEF1α-RiH are available from Addgene (ID 60602-60604).

The target region that contained all TALEN pairs and sgRNAs was cloned into the SSA vector, in which the target region was flanked by the truncated luciferase gene with a homologous region (Sakuma et al., 2013a). A donor template vector for exon 44 knockin of the dystrophin gene (pENTR-DMD-Donor, Addgene 60605) was constructed by conjugating the following four fragments: two homology arms (1 kbp for 3' and 0.9 kbp for 5' arm), an EF1α-hygromycin cassette, and exon 44 fragments, all of which were combined using the In-Fusion HD Cloning Kit (Clontech).

The oligos and primers used are listed in Table S6. For electroporation experiments, plasmid DNAs were purified by a NucleoBond Xtra Maxi Endotoxin-free plasmid DNA purification Kit (TaKaRa) and were prepared as high concentration stock solution of at least 4 $\mu g\ \mu l^{-1}$.

Cell culture

HEK293T (human embryonic kidney 293T) cells were cultured in Dulbecco's modified Eagle's medium (DMEM) supplemented with 5% FBS (fetal bovine serum), 1× non-essential amino acids (NEAA), 100 units ml^{-1} penicillin and 100 μg ml^{-1} streptomycin. Human iPS cells were cultured on mitomycin-C-treated SNL feeder layers in Knockout SR Media {DMEM/F12 media supplemented with 20% Knockout SR, L-glutamine, NEAA, 2-mercaptoethanol, penicillin and streptomycin (Life Technologies), and 8 ng ml^{-1} human basic FGF (Wako)). SNLs were maintained in DMEM containing 6% FBS, 100 units ml^{-1} penicillin, 100 μg ml^{-1} streptomycin and 2 mM GlutaMax. SNL-PH feeder cells (resistant to neomycin, puromycin, and hygromycin) were used for drug selection experiments.

Flow Cytometry

iPS cells were first treated with CTK solution to remove feeder cells, and then single cells were isolated by treatment with 0.25% trypsin-EDTA. Cells were stained with the following antibodies in FACS buffer (5% FBS in PBS) for 20 min at room temperature. The antibodies used were Alexa Fluor 488-conjugated TRA-1-60 (1:20, BD Pharmingen, Cat No. 560174) and FITC-conjugated SSEA-5 (1:20, BioLegend, Cat No. 355207). Unstained cells were used as a negative control.

Quantitative RT-PCR

mRNA was extracted from cells using TRIzol (Invitrogen) reagent and purified by chloroform extraction and isopropanol precipitation. After DNase treatment, the purified RNA was eluted in RNase-Free water and reverse-transcribed using ReverTra Ace (TOYOBO) with random primers and oligo-dT. To quantify the expression of *OCT3/4* and *NANOG*, the level of synthesized cDNAs was measured in isolated samples using SYBA Green (Invitrogen) with the appropriate primers shown in Table S6.

Teratoma formation assay

All mouse experiments were carried out according to protocols approved by the Animal Research Committee of Kyoto University (No. KEI47). For teratoma formation,

sub-confluent human iPS cells were harvested and re-suspended in a 1:2 mixture of culture medium and Matrigel (BD) with 10 μM Y-27632. Around 2 x 10^6 human iPS cells were injected into the tibialis anterior muscles of NOD/SCID/DMD-null mice (NOD/SCID mice crossed with DMD-null mice (Tanaka et al., 2013)). The resulting tumors were harvested from mice 8-9 weeks after transplantation. Samples were fixed in 4% formaldehyde and embedded in paraffin. The sections were stained with hematoxylin and eosin.

Southern blot analysis

Genomic DNA was isolated by phenol-chloroform extraction and ethanol precipitation. Genomic DNAs (15 μg each) were digested with EcoRI for 16 hours, applied on 0.8% agarose gels, and then transferred onto nylon membranes with 20x SSC. The DNA probe (intron 45, 519 bp) was DIG-labeled with the DIG Easy Hyb Kit (Roche) using the pENTR-DMD-Donor as a template with DMD-In45-F and DMD-In45-R primers. DNA hybridization and signal detection were carried out using DIG Easy Hyb and the DIG Chemiluminescent Detection System (ImageQuant LAS 4000 system (GE)).

T7 endonuclease I assay

The target region of TALEN and CRISPR was amplified by a high-fidelity PCR reaction from genomic DNA and purified by Wizard SV Gel and the PCR Clean up System (Promega). The primer sequences for PCR are shown in Table S6. Purified PCR products (400 ng) were denatured (95 ºC for 5 min) and re-annealed (gradually cooled from 95 ºC to 85 ºC at −2 ºC sec^{-1} and 85 ºC to 25 ºC at −0.1 ºC sec^{-1}) in NEBuffer 2 (NEB) using a thermocycler. The re-annealed PCR product was digested by 10 units of T7 endonuclease I (T7EI, NEB) for 15 min at 37 °C. The reaction was stopped by the addition of 0.25 M EDTA solution, and the sample was placed on ice. The PCR products were analyzed on 2% agarose gel to semi-quantify the intensity of the digested and undigested bands by ImageJ software.

Restriction enzyme sensitivity assay

Purified PCR products (generated the same way as in the T7EI assay) were digested by restriction enzyme XcmI (NEB) with NEBuffer 2. The amounts of digested and

undigested PCR products were analyzed by 2% agarose gel electrophoresis. The rate of the area of nuclease-specific cleavage peaks in the sum of the area (expressed as a fraction cleaved) was used to estimate the gene editing levels using the following equation (Reyon et al., 2012).

$$\% \text{ mutation} = 100 \times (1-(1-\text{fraction cleaved})^{1/2})$$

Karyotype analysis

Exponentially growing iPS cell samples (at passage 7 for the original DMD-iPSC clone and at passage 15-23 for the corrected clones) were arrested in metaphase by adding a final concentration of 0.04 μg ml^{-1} KaryoMAX Colcemid Solution (Life technologies) into the culture medium for 3 h at 37 °C. Then, the cells were dissociated with 0.25% trypsin-EDTA solution and pelleted by centrifugation. The pelleted cells were subjected to hypotonic treatment with 0.075 M KCl solution for 30 min at 37 °C. After fixing the cells with fresh methanol/acetic acid (3:1) mixture, metaphase spreads were prepared on pre-cleaned glass slides by dropping a few drops of fixed cells from 1 cm height. Samples were then air dried. Conventional Giemsa staining was used for chromosomal counts and classifications, and the GTG (G-bands after trypsin and Giemsa) technique was used for G-banding. Slides were observed by AxioImagerZ2 microscopy (CarlZeiss) equipped with a CoolCube1m CCD camera (MetaSystems) and Ikaros software (MetaSystems). A total of 50 metaphases were counted for the chromosome counting, and 20 metaphases were observed for the G-band analysis per sample. The cells were described as abnormal if at least two nuclei were found to have the same chromosome aberration. Any samples suspected to be abnormal were further analyzed by multicolor fluorescent *in situ* hybridization (mFISH) analysis using the 24XCyte mFISH Probe Kit (MetaSystems) or by multicolor chromosome banding (mBAND) analysis using an appropriate XCyte 20 mBAND Probe Kit (MetaSystems) for chr20. Fluorescent images were acquired with Isis software (MetaSystems).

SNP array for the DNA copy number analysis

Genomic DNA was isolated from iPS cell samples at passage 20-27 after feeder depletion by Matrigel culture and purified by the DNeasy Blood and Tissue Kit (QIAGEN). SNP genotyping was performed on Illumina HumanOmniExpress 12v1

following the manufacturer's protocol. PennCNV (Wang et al., 2007) software was used to detect CNVs using the following criteria: confidence score > 10, number of SNPs > 10 and minimum length of structural variant > 1 kb. Original CNVs (those called in the original DMD-fibroblasts and uncorrected DMD-iPSCs) and common CNVs (those detected in more than 7 subclones out of the 15 clones analyzed) were discarded. The fragmented CNVs were merged by the clean_cnv.pl script using PennCNV software. We manually checked the values of Log R ratio and B allele frequency at the detected CNVs by IGV software. BEDTools was used to calculate the distance from the potential nuclease target sites to the edge of the detected CNVs and to select random positions in the genome.

Exome sequence analysis

The same genomic DNA samples prepared above were used for exome capture with SeqCap EZ v3 (Roche) following the manufacturer's instructions with minor modification. Briefly, 1 μg of genomic DNA was randomly fragmented by the Covaris E210 system. Adaptor-ligated genomic DNA libraries were prepared with the TruSeq DNA Sample Preparation Kit (Illumina) according to the manufacturer's instructions. The libraries were amplified by the ligation-mediated polymerase chain reaction (LM-PCR) and purified with AMPure XP beads (Agencourt). Human COT-1 DNA and Illumina adaptor-specific blocking oligonucleotides were used during hybridization to block repetitive genomic sequences and adaptor sequences, respectively. The exome-enriched libraries were amplified by LM-PCR, purified with the AMPure XP beads and quantified by a KAPA library quantification Kit (KAPA Biosystems). Ten pM of libraries were used in the cluster generation on HiSeq PE Flow Cell v3 (Illumina) using an Illumina cBot. Paired-end sequencing for 2 × 101 cycles was performed using TruSeq SBS Kit v3 and HiSeq2500 (Illumina). CASAVA 1.8.2 (Illumina) was used to generate FASTQ files from the raw sequencing data.

The sequenced reads in the FASTQ files were mapped to the reference human genome (hg19) by BWA 0.6.2, and identical reads (i.e. PCR duplicates) were removed by Picard 1.68. All samples were generated within 92-120 million reads and had mapping rates over 92% (Table S3). Local re-alignment at the detected indel site was performed by the Genome Analysis Toolkit (GATK) v1.6. Base quality scores were recalibrated with GATK TableRecalibration script. SNVs and indels were called with

GATK UnifiedGenotyper according to the Broad Institute's best-practice v3 guidelines.

The number of nucleotides with a count greater than 30× coverage in the founder cells and iPS clones were subjected to SNV calling using SAMtool mpileup v0.1.19. We discarded variants with lower coverage (< 20 reads in autosomal chromosomes or < 10 reads in sex chromosomes). We calculated the nucleotide composition ratio of each SNV to discriminate homozygous (if one base consisted of more than 77.7%) and heterozygous (if the base compositions of two respective bases were in the range of 22.2-66.6%) SNVs. For indel calling, we considered whether > 40% of sequence reads supported the same indels. We also excluded the common SNVs/indels seen in all samples and the original SNVs/indels derived from the original DMD fibroblasts or DMD-iPSCs. We used ANNOVAR (Wang et al., 2010) to annotate the detected SNVs using the RefSeq, Cosmic ver.65 (Forbes et al., 2011), HGMD (Stenson et al., 2003) and dbSNP ver.137 (Sherry et al., 2001) databases.

Immunocytochemistry

Cell samples seeded on cover slips were fixed with chilled methanol for 15 min and blocked by Blocking One (Nacalai) for 30 min at 4 °C. Cells were then stained with the appropriate primary antibodies for 16 hours at 4 °C and then with secondary antibodies for 1 hour at room temperature. Coverslips were then washed and observed in 1x PBS by confocal microscopy (Zeiss LSM 710). The primary antibodies used were: NCL-DYS1 (1:20; clone Dy4/6D3, Leica), MHC (1:400; R&D systems), MYH (1:200; clone H-300, Santa Cruz Biotechnology) and α-skeletal muscle actin (1:200; Acris Antibodies). The secondary antibodies used were Alexa fluor 488 goat-anti-mouse (1:500; Invitrogen) for dystrophin protein and Alexa fluor 546 goat-anti-rabbit (1:500; Invitrogen) for MHC or MYH. DAPI (4,6-diamidino-2-phenylindole; Invitrogen) was used for nuclear counterstaining.

Western blot analysis

Cell samples were lysed with RIPA buffer (Thermo) supplemented with a protease inhibitor cocktail (Calbiotech). The total amount of protein was quantified by the Pierce BCA Protein Assay Kit (Thermo). Samples were then mixed with loading buffer and heated at 95 °C for 10 min. Approximately 40 µg of protein samples per lane were separated on NuPAGE 3-8% Tris-Acetate Gel (Invitrogen) with NuPAGE®

Tris-Acetate SDS running buffer (Invitrogen) and transferred to nitrocellulose membranes using iBlot (Invitrogen) for 13 min. Then, membranes were blocked by Blocking One for 1 hour at room temperature. The primary antibodies used were anti-dystrophin (1:200, Abcam) and anti-MHC (1:400, R&D systems). The secondary antibodies used were anti-rabbit IgG HRP-linked antibody and anti-mouse IgG HRP-linked antibody (1:1000, Cell Signaling). The protein expression was detected using the ImageQuant LAS 4000 system (GE).

Supplemental References

Forbes, S., Bindal, N., Bamford, S., Cole, C., Kok, C., Beare, D., Jia, M., Shepherd, R., Leung, K., Menzies, A., *et al.* (2011). COSMIC: mining complete cancer genomes in the Catalogue of Somatic Mutations in Cancer. Nucleic Acids Res *39*, D945-950.

Reyon, D., Tsai, S., Khayter, C., Foden, J., Sander, J., and Joung, J. (2012). FLASH assembly of TALENs for high-throughput genome editing. Nat Biotechnol *30*, 460-465.

Sakuma, T., Hosoi, S., Woltjen, K., Suzuki, K., Kashiwagi, K., Wada, H., Ochiai, H., Miyamoto, T., Kawai, N., Sasakura, Y., *et al.* (2013a). Efficient TALEN construction and evaluation methods for human cell and animal applications. Genes Cells *18*, 315-326.

Sakuma, T., Ochiai, H., Kaneko, T., Mashimo, T., Tokumasu, D., Sakane, Y., Suzuki, K., Miyamoto, T., Sakamoto, N., Matsuura, S., *et al.* (2013b). Repeating pattern of non-RVD variations in DNA-binding modules enhances TALEN activity. Sci Rep *3*, 3379.

Sherry, S., Ward, M., Kholodov, M., Baker, J., Phan, L., Smigielski, E., and Sirotkin, K. (2001). dbSNP: the NCBI database of genetic variation. Nucleic Acids Res *29*, 308-311.

Stenson, P., Ball, E., Mort, M., Phillips, A., Shiel, J., Thomas, N., Abeysinghe, S., Krawczak, M., and Cooper, D. (2003). Human Gene Mutation Database (HGMD): 2003 update. Hum Mutat *21*, 577-581.

Tanaka, A., Woltjen, K., Miyake, K., Hotta, A., Ikeya, M., Yamamoto, T., Nishino, T., Shoji, E., Sehara-Fujisawa, A., Manabe, Y., *et al.* (2013). Efficient and reproducible myogenic differentiation from human iPS cells: prospects for modeling Miyoshi Myopathy in vitro. PLoS One *8*, e61540.

Wang, K., Li, M., Hadley, D., Liu, R., Glessner, J., Grant, S., Hakonarson, H., and Bucan, M. (2007). PennCNV: an integrated hidden Markov model designed for high-resolution copy number variation detection in whole-genome SNP genotyping data. Genome Res *17*, 1665-1674.

Wang, K., Li, M., and Hakonarson, H. (2010). ANNOVAR: functional annotation of genetic variants from high-throughput sequencing data. Nucleic Acids Res *38*, e164.
