# Supplementary material for: Precise Correction of the Dystrophin Gene in Duchenne Muscular Dystrophy Patient Induced Pluripotent Stem Cells by TALEN and CRISPR-Cas9
Source: Stem Cell Reports. 2014 Nov 26;4(1):143–54. doi: 10.1016/j.stemcr.2014.10.013 (PMC4297888; doi:10.1016/j.stemcr.2014.10.013)
Supplement: Document S2. Article plus Supplemental Information [file mmc2.pdf]

Stem Cell Reports
Resource
OPEN ACCESS

Precise Correction of the Dystrophin Gene in Duchenne Muscular Dystrophy Patient Induced Pluripotent Stem Cells by TALEN and CRISPR-Cas9

Hongmei Lisa Li,[1] Naoko Fujimoto,[1,2] Noriko Sasakawa,[1] Saya Shirai,[1] Tokiko Ohkame,[1] Tetsushi Sakuma,[4] Michihiro Tanaka,[1] Naoki Amano,[1] Akira Watanabe,[1] Hidetoshi Sakurai,[1] Takashi Yamamoto,[4] Shinya Yamanaka,[1,2,5] and Akitsu Hotta[1,2,3,*]
[1]Center for iPS Cell Research and Application, Kyoto University, Kyoto 606-8507, Japan
[2]iCeMS, Kyoto University, Kyoto 606-8501, Japan
[3]PRESTO, Japan Science and Technology Agency, Kawaguchi 332-0012, Japan
[4]Department of Mathematical and Life Sciences, Graduate School of Science, Hiroshima University, Higashi-Hiroshima 739-8526, Japan
[5]Gladstone Institute of Cardiovascular Disease, San Francisco, CA 94158, USA
*Correspondence: akitsu.hotta@cira.kyoto-u.ac.jp
http://dx.doi.org/10.1016/j.stemcr.2014.10.013
This is an open access article under the CC BY license (http://creativecommons.org/licenses/by/3.0/).

SUMMARY

Duchenne muscular dystrophy (DMD) is a severe muscle-degenerative disease caused by a mutation in the dystrophin gene. Genetic correction of patient-derived induced pluripotent stem cells (iPSCs) by TALENs or CRISPR-Cas9 holds promise for DMD gene therapy; however, the safety of such nuclease treatment must be determined. Using a unique *k*-mer database, we systematically identified a unique target region that reduces off-target sites. To restore the dystrophin protein, we performed three correction methods (exon skipping, frameshifting, and exon knockin) in DMD-patient-derived iPSCs, and found that exon knockin was the most effective approach. We further investigated the genomic integrity by karyotyping, copy number variation array, and exome sequencing to identify clones with a minimal mutation load. Finally, we differentiated the corrected iPSCs toward skeletal muscle cells and successfully detected the expression of full-length dystrophin protein. These results provide an important framework for developing iPSC-based gene therapy for genetic disorders using programmable nucleases.

INTRODUCTION

Duchenne muscular dystrophy (DMD) is a severe muscular degenerative disease caused by loss-of-function mutations in the dystrophin gene located on the X chromosome. The dystrophin gene consists of 79 exons, and disruption of the protein reading frame by small deletions, exon duplications, or loss of exons leads to DMD (Pichavant et al., 2011). The large size of the dystrophin gene hampers the delivery of therapeutic cDNA for gene augmentation. Therefore, the delivery of truncated microdystrophin or microutrophin by an adeno-associated viral (AAV) vector (Okada and Takeda, 2013), lentiviral vector (Pichavant et al., 2011), or *Sleeping Beauty* transposon (Filareto et al., 2013) has been investigated for DMD gene therapy. However, restoration of the full-length dystrophin protein remains challenging. An exon-skipping approach that modulates mRNA splicing patterns using antisense oligonucleotides (Aartsma-Rus et al., 2009) has shown promising results in preclinical studies, but the effects are only transient. Genomic correction using programmable nucleases is an ideal approach that can correct the mutated dystrophin gene.

The development of programmable nucleases has provided a powerful tool for modifying target genome sequences. In particular, the transcription activator-like effector nuclease (TALEN) (Hockemeyer et al., 2011) and the clustered regularly interspaced short palindromic repeat (CRISPR) and CRISPR associated 9 (Cas9) endonuclease systems (Cong et al., 2013; Mali et al., 2013) provide greater flexibility than meganucleases or zinc-finger nucleases (ZFNs) with regard to selecting the target regions of interest (Li et al., 2014). Several studies have demonstrated the effectiveness of TALENs (Hockemeyer et al., 2011; Ding et al., 2013a) and CRISPR (Ding et al., 2013b; Mali et al., 2013) in human induced pluripotent stem cells (iPSCs) for reporter knockin, gene knockout, and gene correction. In fact, corrections of disease mutation by nucleases in iPSCs have been reported for several diseases, including α1-antitrypsin deficiency (Choi et al., 2013), epidermolysis bullosa (Osborn et al., 2013), β-thalassemia (Ma et al., 2013), AIDS (Ye et al., 2014), and Niemann-Pick Type C (Maetzel et al., 2014).

Before the TALEN and CRISPR systems can reach clinical application, however, target specificity must be improved, as high off-target mutagenesis rates in human cells have been reported (Fu et al., 2013; Hsu et al., 2013; Lin et al., 2014), although some reports have shown otherwise (Smith et al., 2014; Suzuki et al., 2014; Veres et al., 2014). Since target specificity depends on the design of the target site, the properties of the DNA-binding domain, and the epigenetic status of the targeting site, the risk of off-target mutagenesis should be examined with respect to each targeting nuclease in a therapeutic setting.

CrossMark

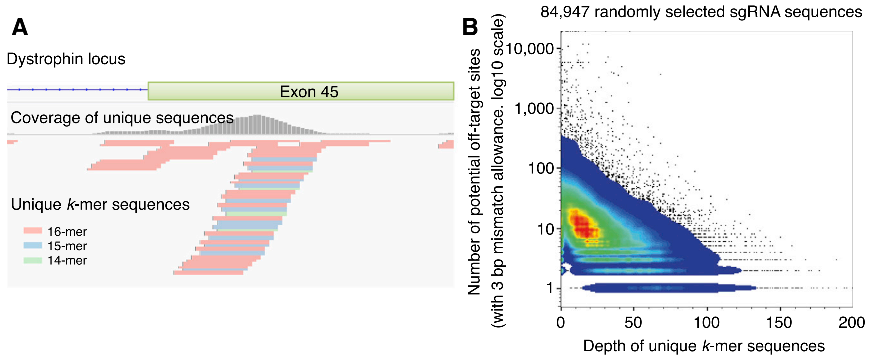

Figure 1. Visualization of the Unique Sequences in the Human Genome
(A) Example of the mapped unique *k*-mer sequences at the exon 45 region of the dystrophin gene. The mapped *k*-mers are indicated in the bottom panel and the coverage of the *k*-mers in each base position is indicated by the gray histogram. Within this region, unique sequences of 14- to 16-mer were identified.
(B) Pseudocolor dot plot for the depth of unique *k*-mers and the number of potential off-target sites. CRISPR-sgRNA targeting sequences (23 bp with "NGG" PAM, n = 84,947) were randomly selected from the human genome and the depth of the unique *k*-mers and number of potential off-target sites (with up to 3 bp mismatches allowance) for each sgRNA sequence were calculated. Note that higher depth correlated with fewer potential off-target sites. See also Table S1.

Immortalized myoblasts have been used for restoration of the dystrophin protein mediated by meganucleases (Rousseau et al., 2011; Popplewell et al., 2013), ZFNs (Rousseau et al., 2011), or TALENs (Ousterout et al., 2013). However, although primary myoblasts can be derived from patients, their clonal expansion requires transformation by oncogenes such as hTERT. In contrast, iPSCs (Takahashi et al., 2007) can be isolated from patients directly and still maintain pluripotency and an unlimited self-renewal capacity. Accordingly, when conjugated with made-to-order genetic correction technologies, human iPSCs derived from a patient with a genetic disorder (Park et al., 2008; Hotta et al., 2009) might be applicable to autologous transplantation as ex vivo gene therapy (Sebastiano et al., 2011; Soldner et al., 2011; Zou et al., 2011a, 2011b).

In this study, as a proof-of-concept of such gene therapy for DMD, we performed and demonstrated genetic correction of the dystrophin gene in patient-derived iPSCs by using three different methods: (1) disruption of the splicing acceptor to skip exon 45, (2) introduction of small indels to modulate the protein reading frame, and (3) knockin of the missing exon 44 to restore the full protein coding region. We then performed comprehensive genome-wide mutation analyses to assess the risk of off-target mutagenesis in 14 iPSC clones treated according to the TALEN or CRISPR approach. Our results demonstrate that genetic correction by these approaches in patient-derived iPSCs considerably lowers the risk of off-target mutagenesis and thus holds promise for DMD gene therapy.

RESULTS

Targeting a Unique Region in the Human Genome

The risk of off-target mutagenesis by programmable nucleases is associated with the specificity of the target sequence. For example, in the 23 bp of the single-guide RNA (sgRNA) targeting region, up to 5 bp mismatches may be tolerated, which may lead to off-target mutagenesis (Fu et al., 2013; Hsu et al., 2013). To avoid this, the target sequence must be uniquely defined in the genome. Moreover, the uniqueness should be preserved when considering fragments of the sequence (i.e., 15 bp in length). Therefore, to systematically identify short unique sequences in the genome, we computationally generated all possible combinations of short *k*-mer sequences ($k \leq 16$) and searched the human genome to determine how many identical sequences are found for each *k*-mer sequence. We then extracted the unique *k*-mer sequences only when they matched a single location (i.e., with no match to other regions; see Table S1 available online). We stacked the mapped *k*-mer sequences as a histogram to visualize their uniqueness in the sequence depth of coverage (Koehler et al., 2011; Figure 1A). We confirmed that the higher the depth of the unique *k*-mer, the lower was the number of off-target sites, with up to 3 bp mismatches allowed (Figure 1B). Therefore, the genome regions with a higher depth of unique *k*-mers were considered good candidates for targeting by programmable nucleases, and regions with no peak were not considered. The benefit of this method is that it allows one to visually identify the targetable site quickly. Based on the histogram of the unique *k*-mers, we identified the 5′ region of exon 45 in the dystrophin gene as a target site for designing TALENs and CRISPR-sgRNAs (Figures 2B and S2A).

Generation of Integration-free DMD iPSCs

A DMD patient was diagnosed with a deletion of dystrophin exon 44 by a multiplex ligation-dependent probe amplification method. We performed a primer walking method to sequence the deleted region and identified that the deleted size was 75,484 bp (chrX: 32,215,020-32,290,503 [hg19]), including exon 44 (Figure S1A). We generated iPSC lines from fibroblasts obtained from this DMD patient using

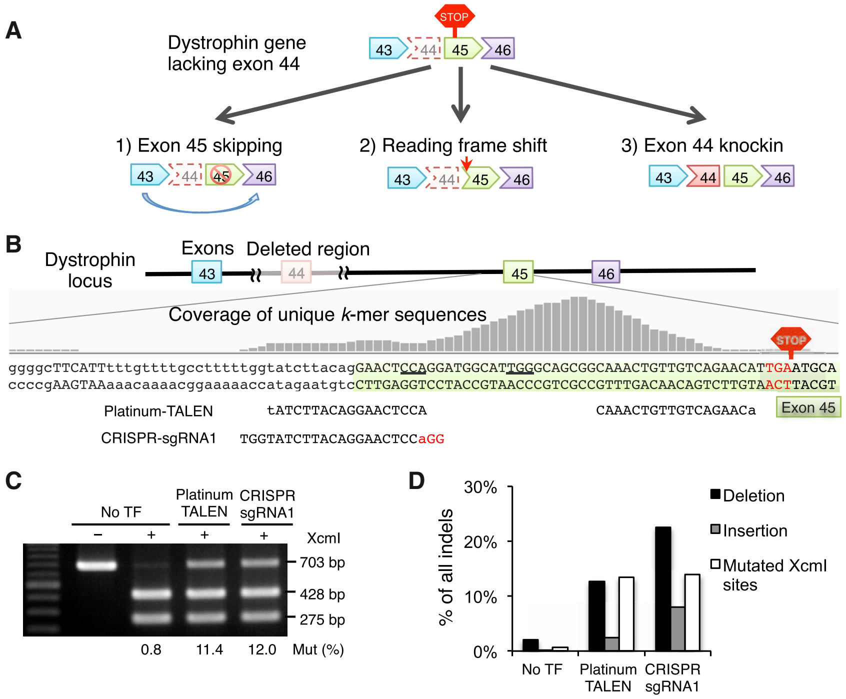

Figure 2. TALEN and CRISPR-sgRNA Are Equally Active for Targeting the Dystrophin Gene in iPSCs

(A) The three strategies used to restore the reading frame of the dystrophin protein.

(B) We designed the Platinum-TALEN pair and CRISPR-sgRNA1 within the peak of the unique *k*-mer sequences, as indicated by the gray histogram, and in front of the de novo premature stop codon, as indicated by the red hexagon.

(C) The activities of Platinum-TALEN and CRISPR-sgRNA1 were analyzed by a restriction enzyme (XcmI) sensitivity assay. The XcmI digestion site (CCANNNNNNNNNTGG) was located in the spacer of the TALEN and next to the PAM sequence of the CRISPR-sgRNA1. The intensity percentage of the undigested band (703 bp) was used to calculate the mutation efficiency.

(D) The frequency of deletions (black bar) and insertions (gray bar) was analyzed by deep sequencing for the region flanking the target site. The percentages of sequence reads corresponding to deletions, insertions, and mutated XcmI sites (white bar) are plotted. No TF, nontransfected control.

See also Figure S2.

the integration-free episomal vector method (Okita et al., 2011). We chose iPSC lines that maintained a normal karyotype (Figure S1B) and expressed pluripotency markers, including TRA-1-60, SSEA5, *OCT3/4*, and *NANOG* (Figures S1C and S1D). Pluripotency was also confirmed by the in vivo teratoma formation assay (Figure S1E).

Strategies for Dystrophin Correction

To restore the dystrophin protein reading frame in iPSCs from the DMD patient who lacked exon 44, we devised three approaches: the first was to disrupt the splicing acceptor of exon 45, as the connection of exons 43 and 46 would restore the reading frame; the second was to induce a frameshift by introducing small indels (insertions or deletions); and the third was to insert exon 44 in front of exon 45 (Figure 2A). We tested 15 pairs (five left and three right) of TALENs using the Golden-Gate assembly method (Golden-TALENs) (Sakuma et al., 2013a) and one pair of Platinum-Gate-based TALENs (Platinum-TALENs) (Sakuma et al., 2013b), which had nonrepeat-variable diresidue variations on the TAL domain to enhance the activity. We found that the E/a pair of Golden-TALENs and Platinum-TALENs showed the highest recombination activity, as assessed by the single-strand-annealing (SSA) assay, in human embryonic kidney 293T (HEK293T) cells (Figures S2B and S2C).

We also constructed five CRISPR-sgRNAs adjacent to the TALEN cleavage sites and tested the cleavage activities using the SSA assay (Figure S2C). There were no significant differences in the cleavage activities among the constructed CRISPR-sgRNAs. Therefore, we used sgRNA1 for later experiments because it was located within exon 45 and closer to the splicing acceptor than the other sequences. We then compared the activity of Platinum-TALEN and CRISPR-sgRNA1 by performing a restriction enzyme sensitivity assay, and found that both had similar activity in DMD iPSCs (Figure 2C).

When we examined the mutation patterns by deep amplicon sequencing of the target region, we found that both TALEN and CRISPR gave similar percentages of "mutated XcmI sites," which was consistent with the XcmI-digested sensitivity assay (Figures 2C and 2D). Interestingly, CRISPR induced a slightly higher indel rate compared with TALEN (Figure 2C), which suggested that the restriction enzyme sensitivity assay is sensitive to the relative position of the nuclease-targeting site and to restriction enzyme sites that differ by only a few basepairs. We also noted that TALEN produced fewer insertions compared with CRISPR-Cas9 (Figure 2D).

Correction of the Reading Frame Mutation Solely by Nuclease Treatment

We next utilized the programmable nucleases to restore the reading frame via disruption of the splicing acceptor (exon skipping) or induction of a (3*n* + 1) bp frameshift (where

A

Experiment	Programmable nucleases	iPS cell line	Analyzed clones	Clones with indels	In-frame clones	Exon skipping clones
1	Golden-TALEN (with puro selection)	DMD-c1 (Retroviral iPSCs)	88	28 (31.8%)	7	0
2	Platinum-TALEN	DMD-iPSC #1	96	14 (14.6%)	3	4
3	CRISPR-sgRNA1	DMD-iPSC #1	45	8 (17.7%)	2	2
Total			229	40 (17.5%)	12	6

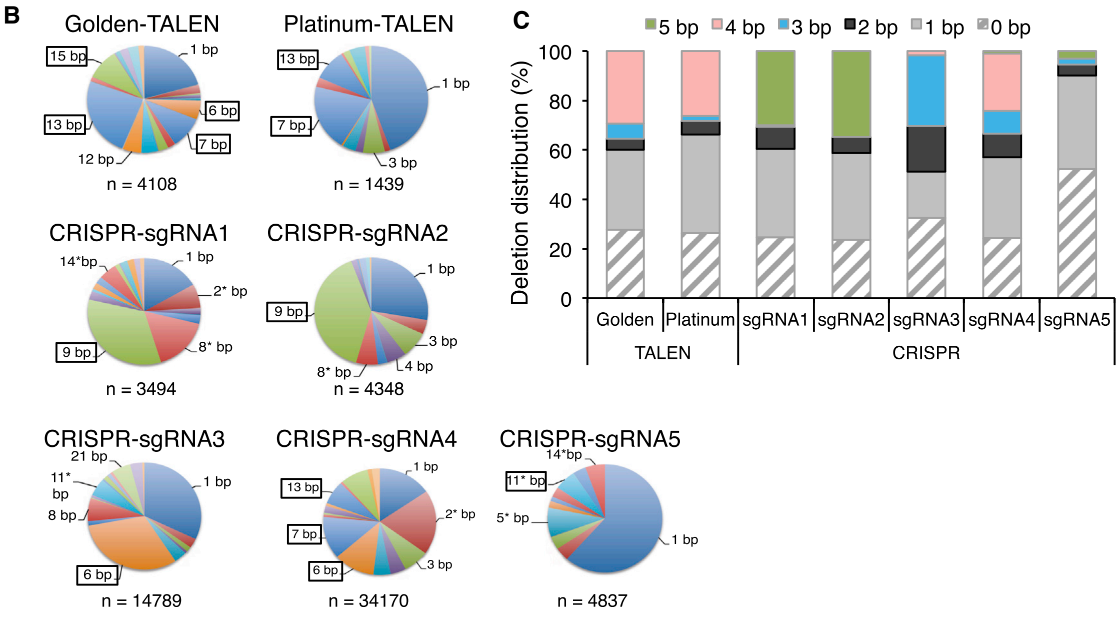

Figure 3. Correction of the Dystrophin Gene by Exon Skipping and Frameshifting Solely by Nuclease Treatment

(A) Summary of the exon-skipping (ES) and in-frame (IF) correction approaches using the indicated nucleases without the donor template. The DMD-c1 iPSC clone was derived by a retroviral method and the DMD-iPSC #1 (CiRA00111) clone was derived by an integration-free episomal vector method. Sanger sequence analyses were performed for all picked clones to identify subclones that had indels. The number of ES and IF clones is indicated.

(B) The deletion patterns induced by TALENs and CRISPR-sgRNAs were analyzed by deep sequencing of the target site. The numbers around the pie charts indicate the deletion sizes found in more than 5% of sequence reads, and the numbers with an asterisk indicate deletions that could restore the reading frame of the dystrophin gene. The boxed numbers indicate the deletion patterns flanked with local microhomologies.

(C) For each of the sequence reads obtained by deep sequencing, microhomology motifs on both sides of the deletion were retrieved and the distribution of the microhomology sizes was plotted.

See also Figure S3 and Table S2.

n is a nonnegative integer) without a donor template (frameshifting). Platinum-TALEN or CRISPR/Cas9-sgRNA1 was introduced into DMD iPSCs and the subclones were screened by Sanger sequencing of the target genomic DNA (Figures S3A and S3B). As summarized in Figure 3A, a total of 229 iPSC clones were analyzed, and 40 clones showed indels at the target site. Among the 40 clones with indels, six were identified as exon-skipping (ES) clones and 12 were identified as frameshift-induced in-frame (IF) clones. We chose two clones (ES^{H19} and ES^{H29}) with an 18 bp deletion spanning the splicing acceptor site of exon 45, and three clones (IF^{H13}, IF^{H30}, and IF^{D28}) with a 1 bp insertion as IF clones with minimal alterations to the amino acid sequence (Figure S3B). We confirmed the pluripotency of the genetically corrected clones by examining their expression of *OCT3/4*, *NANOG*, TRA-1-60, and SSEA5 (Figures S1D and S3C). Functional pluripotency was also confirmed by a teratoma formation assay (Figure S3D).

Deletion Patterns Induced by TALENs and CRISPRs

In our indel pattern analysis, we observed that several clones harbored identical indel patterns mediated by flanking microhomology motifs. To further assess the indel

A

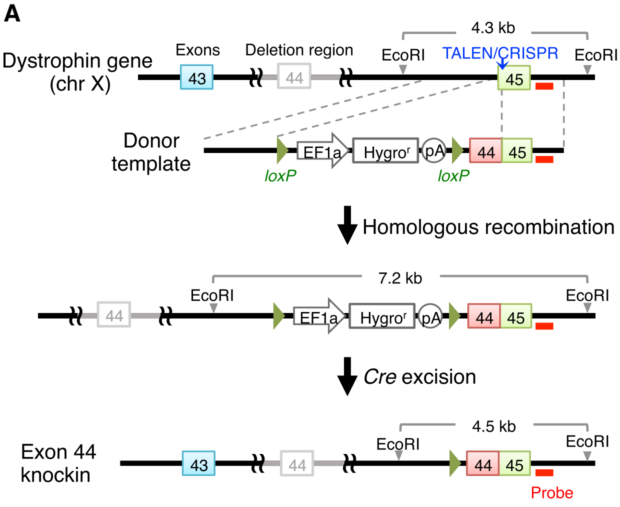

B

Knockin experiments		Positive ratio by PCR	Single copy ratio by Southern blot	*loxP* excison by *Cre* expression
TALEN-mediated	Golden	95.8% (46/48)	87.5% (7/8)	60.0% (18/30)
	Platinum	89.6% (43/48)	44.4% (4/9)	47.8% (11/23)
CRISPR-mediated	sgRNA1	75% (9/12)	50% (4/8)	65% (23/35)

C

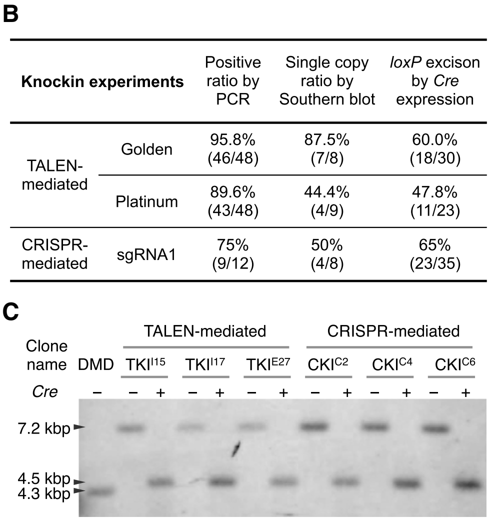

Figure 4. Correction of the Full-Length Dystrophin Protein by Exon 44 Knockin

(A) Schematic overview depicting the exon 44 knockin strategy. Top line: structure of the dystrophin gene with the exon 44 deletion. The vertical blue arrow indicates the TALEN/CRISPR cut site and the red bar indicates the intron 45 probe used for Southern blot analysis. Note that we introduced silent mutations at the nuclease-targeting site within exon 45 of the donor template.

(B) Summary of the exon 44 knockin experiments. The picked clones were first screened by genomic PCR for targeted knockin of the donor template and then by Southern blotting with EcoRI digestion and the intron 45 probe to confirm no additional integration. Successfully targeted clones were further treated with *Cre* to remove the drug selection cassette flanked by the *loxP* elements.

(C) Southern blot of the knockin clones (clone names: TKI^{I15}, TKI^{I17}, and TKI^{E27} for TALEN-mediated knockin clones; CKI^{C2}, CKI^{C4}, and CKI^{C6} for CRISPR-mediated knockin clones) showing successful targeting at the designated site. Subsequent *Cre* treatment excised the *loxP*-flanked drug selection cassette. The probe in intron 45 was used to detect EcoRI-digested genomic DNA fragments.

See also Figures S3B and S3C.

patterns, we isolated the genomic DNA 2–3 days after nuclease transfection (to avoid the effects of cellular expansion) and analyzed them by deep sequencing of amplicons. We observed several variations of indel patterns, but some sizes of deletions were enriched in both TALEN- and CRISPR-treated samples (Figure 3B). Computational analysis of the nuclease target site (In silico Genome Editing and Analysis Tools, https://apps.cira.kyoto-u.ac.jp/igeats/) led us to observe that microhomology motifs existed on both sides of the deletion site (Table S2), consistent with a recent report (Bae et al., 2014a). In our case, we found that the 3–5 bp microhomology-mediated deletions led to more than 30% of the deletion events analyzed (Figure 3C). This observation suggests that a cleavage site flanked by microhomology sequences can generate preferential deletion patterns, potentially via microhomology-mediated repair.

Correction of the Full-Length Dystrophin Protein with a Donor Template

The deletion of exon 44 is the third or fourth most common deletion in DMD patients (Aartsma-Rus et al., 2009; Tuffery-Giraud et al., 2009); however, the breakpoints of each deletion vary among patients. In order to restore the full amino acid sequence of the dystrophin protein for DMD patients who lack exon 44, we attempted to knock in the deleted exon 44 in front of exon 45. We constructed a donor template vector to conjugate exon 45 with exon 44 to share the same splicing acceptor site together with a hygromycin-selection cassette flanked by two *loxP* sites (Figure 4A). We utilized Golden-TALEN (E/a), Platinum-TALEN, and CRISPR-sgRNA1 for the knockin experiments.

The targeting donor was coelectroporated with TALENs or Cas9/sgRNA expression vectors. After hygromycin selection and limiting dilution, we isolated several clones and screened for knockin clones by PCR analysis. Regardless of which nuclease we used, up to 90% of the analyzed clones (46/48 for Golden-TALEN (E/a), 43/48 for Platinum-TALEN, and 9/12 for CRISPR-sgRNA1) showed targeting of the donor template at the target locus. We further confirmed single-copy knockin clones by Southern blot analysis and found that approximately 44%–87% of the clones had a single copy at the targeted site (Figure 4B). We chose two clones (TKI^{I15} and TKI^{I17}) mediated by Golden-TALEN (E/a), one clone (TKI^{E27}) mediated by Platinum-TALEN, and three clones (CKI^{C2}, CKI^{C4} and CKI^{C6}) mediated by CRISPR-sgRNA1 for the subsequent experiments.

To remove the selection cassette from the knockin clones, we transiently treated the obtained clones with the *Cre* expression vector to excise the hygromycin-selection cassette. Successful excision was confirmed by genomic PCR and Southern blotting (Figure 4C). We also sequenced the chimeric exon 44-45 in the knockin clones by Sanger sequencing, and detected no extra mutations except for the five silent mutations (underscored in Figure S3B) that were designed to prevent the sequence from being recut by our programmable nucleases. In addition, we checked the expression of the pluripotency markers *OCT3/4*, *NANOG*, TRA-1-60, and SSEA5 in the knockin clones (Figures S1D and S3C).

Analyses of Off-Target Mutagenesis Induced by TALENs or CRISPRs

The risk of undesired off-target mutagenesis is one of the most important concerns for the application of genome-editing technologies, and especially for gene therapy. We designed our experiments so that all genetically corrected clones originated from a single DMD-iPSC clone, which allowed us to distinguish preexisting genetic variations or culture artifacts in iPSCs (Laurent et al., 2011; Sugiura et al., 2014) from nuclease-mediated mutagenesis. The off-target effects were classified into two categories: target-sequence-dependent and -independent mutations. To assess the former, we searched for potential off-target sites with several mismatches in the human genome using the Bowtie program. For the Platinum-TALEN and Golden-TALEN E/a pair target sequences, we found thousands of potential off-target sites containing up to three mismatches for one side of TALEN. However, no pairs were located within 100 bp to form the FokI dimer. For the CRISPR-sgRNA1, we found five predicted off-target sites containing up to three mismatches (Figure S4A).

To investigate whether these potential off-target sites were actually mutated, we amplified the predicted off-target regions and analyzed them using the T7 endonuclease I (T7EI) assay. In contrast to the on-target cleavage, four predicted off-target sites showed no detectable mutagenesis, whereas one locus had a homozygous insertion of the AluYb9 element with a poly A tail (39-mer) (Figure S4B). The poly A stretch generated a heteroduplex when the PCR products were reannealed during the T7EI assay, resulting in digestion by T7EI even in the nontransfected control. We confirmed that the insertion of AluYb9 occurred in the original DMD fibroblasts and DMD iPSCs, but not in Caucasian-derived control iPSCs (Takahashi et al., 2007; Figure S4C).

To investigate the presence of minor mutations that were not detectable by the T7EI assay, we performed deep sequencing of the amplicons at the off-target sites. No significant indel reads were observed above the background level of the nontransfected control (Figure S4D).

Next, we examined whether nuclease treatment affected the chromosomal structure at the single-cell level. We first checked the karyotype of the genetically modified clones by performing a chromosome count with conventional Giemsa staining and high-resolution G-banding. Several suspected clones identified by G-banding were further analyzed by multicolor fluorescence in situ hybridization or multicolor banding. The majority of the modified cells (144/153, 94.1%) maintained a normal karyotype (Figures 5A and S5), but a few (9/153, 5.9%) showed a suspected or abnormal karyotype in three clones (out of seven modified clones analyzed; Figures S5D and S5E). Interestingly, it was previously reported that an amplification of chr20q11.21 was associated with a growth advantage for human embryonic stem cells (Amps et al., 2011). With the TKI^{I17} clone, we observed an inverted insertion at chr20q11.2 (ins(20)p11.2q13.3q12) in two of the 30 metaphases we analyzed. This insertion may have been acquired during the subcloning process.

It was previously reported that TALENs or CRISPR-Cas9 may induce up to 1 Mbp of deletions if two separated sites are targeted simultaneously (Canver et al., 2014). To elucidate whether the off-target cleavage of TALENs or CRISPR-Cas9 induced such large deletions or duplications, we investigated DNA copy number variations (CNVs) in the corrected clones by TALEN or CRISPR treatment with a high-density SNP array. We detected several CNVs compared with the reference data (human population mean), including the 75 kbp deletion of dystrophin exon 44, validating our analysis (Figure S6).

We focused on the de novo CNVs that were not present in the original DMD iPSCs, and found no major differences in the number of CNVs among the three corrected groups (Figure S6B). We employed three control clones that were subjected to similar electroporation and limiting dilution processes as the dystrophin-corrected clones, but were unmodified at the target site. To determine whether there was any correlation between the potential off-target sites and the observed CNVs, we measured the distance between the edges of the CNVs and the potential nuclease cleavage sites. The distributions for each nuclease were comparable to the corresponding numbers of randomly selected genomic loci (Figure 5B).

Furthermore, we sequenced the whole protein-coding regions of the original DMD-iPSC clone and their subclones by exome sequencing (Table S3). To assess the single nucleotide mutations associated with the treatment of programmable nucleases, we used the parental DMD iPSC as a reference and extracted the de novo mutations observed only in the offspring clones. From this analysis,

A Karyotype analyses for DMD-iPSC and the corrected clones

Samples	Cell type	Chromosome number					G-banding		
		44	45	46	47	48	Normal	Abnormal	Examined
DMD-Fibroblast	Original fibroblast		3	46	1		20		20
DMD-iPSC #1	Original iPSC			50			20		20
IF^H13	TALEN in-frame			50			18	5	23
IF^H30				50			18	2	20
IF^D28				50			20		20
TKI^I15	TALEN knockin			50			20		20
TKI^I17				50			28	2	30
CKI^C2	CRISPR knockin	1	2	46		1	20		20
CKI^C4			2	48			20		20
Subtotal		1	4	344	0	1	144	9	153

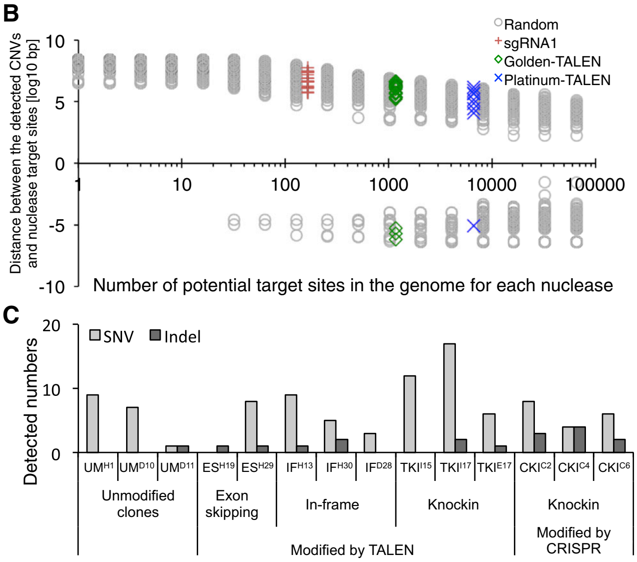

Figure 5. Multiple Whole-Genome Analyses Revealed No Severe Off-Target Mutagenesis

(A) Summary of the karyotyping analyses of the corrected clones. Conventional Giemsa staining was used for chromosome counting (50 metaphases were counted), and high-resolution G-banding was applied to detect chromosomal rearrangements (at least 20 metaphases were analyzed).

(B) SNP array analysis identified de novo CNVs in the corrected clones. To determine associations between detected CNVs and potential off-target sites, the distances between the edges of the detected CNVs and potential nuclease target sites are plotted for each nuclease (red cross for sgRNA1, green diamond for Golden-TALEN, and blue × mark for Platinum-TALEN). Since the likelihood of the distance distribution depends on how many genomic sites are selected, we also selected random genomic positions and calculated the distance for each CNV (gray circles). A zero value in the y axis indicates the edge of the CNVs and a negative value indicates the inside of the CNVs. If any given nuclease off-target site is associated with the detected CNVs, the target site should approximate zero distance.

(C) The number of single nucleotide variants (SNVs) and small indels detected by exome sequencing. Only de novo SNVs and indels that did not exist in the original DMD fibroblasts or DMD iPSCs are plotted. Unmodified control clones underwent the same electroporation and subcloning process as the other corrected clones and were sequenced at similar passage numbers.

See also Figures S4–S6 and Tables S3–S5.

we identified a few dozen point mutations (both synonymous and nonsynonymous) and several indels from the exome sequencing data (Figure 5C; Tables S4 and S5). Importantly, we successfully detected the 1 bp insertion and 18 bp deletion at the dystrophin exon 45 in the IF and ES clones, respectively (Table S5, labeled in blue). We observed slightly higher indel events ($p < 0.05$, one-way ANOVA) in the CRISPR knockin clones (average 3.0) compared with the unmodified controls (average 0.33). However, most of the detected indels were in the triple-repeat or GC-rich regions and were not associated with potential off-target sites, which is consistent with recent reports (Smith et al., 2014; Veres et al., 2014). In addition, there were no significant differences in the number of SNVs among the three correction approaches and the control group ($p > 0.05$, one-way ANOVA), which is also in agreement with recent publications (Smith et al., 2014; Veres et al., 2014). Taken together, these results show that no severe off-target mutagenesis was associated with potential nuclease-targeting sites in the dystrophin-corrected iPSC clones.

The Dystrophin Protein Was Restored in Differentiated Skeletal Muscle Cells

To confirm that genetic correction by TALEN and CRISPR resulted in restoration of the dystrophin gene products, we differentiated the original and corrected iPSC clones into skeletal muscle cells using our recently published method (Tanaka et al., 2013). Differentiated skeletal muscle cells were collected on day 9 of differentiation to isolate mRNA. Amplification using PCR primers spanning exons 43 and 46 revealed that the exon 44 knockin clone (CKI^{C2}) had restored the full-length dystrophin mRNA to the same length as that of a healthy individual control ("Healthy"; Figure 6A). Sanger sequence analysis of the cDNA also showed the corresponding correction, where the 1 bp insertion of "A" in the IF clone (IF^{H30}), the 18 bp deletion that induced the skipping of exon 45 (ES^{H29}), and the knockin clone (TKI^{I15} and CKI^{C2}) all led to successful expression of the inserted exon 44 together with exon 45 (Figure 6B).

Finally, to detect the restored dystrophin protein, we performed immunofluorescence staining with an anti-dystrophin antibody (Dys1), which recognizes the rod domain (amino acids 1,181–1,388) of the dystrophin protein (Figure 6C). The restored dystrophin protein was localized at the submembrane region, as expected, in all corrected clones examined. Furthermore, we performed a western blot analysis with an anti-dystrophin antibody, which recognizes amino acids 3,661–3,677, and detected bands at the following predicted sizes: 420 kDa for the reading-frame corrected clone, 414 kDa for the ES clone, and 427 kDa for the knockin clone (Figure 6D). As expected,

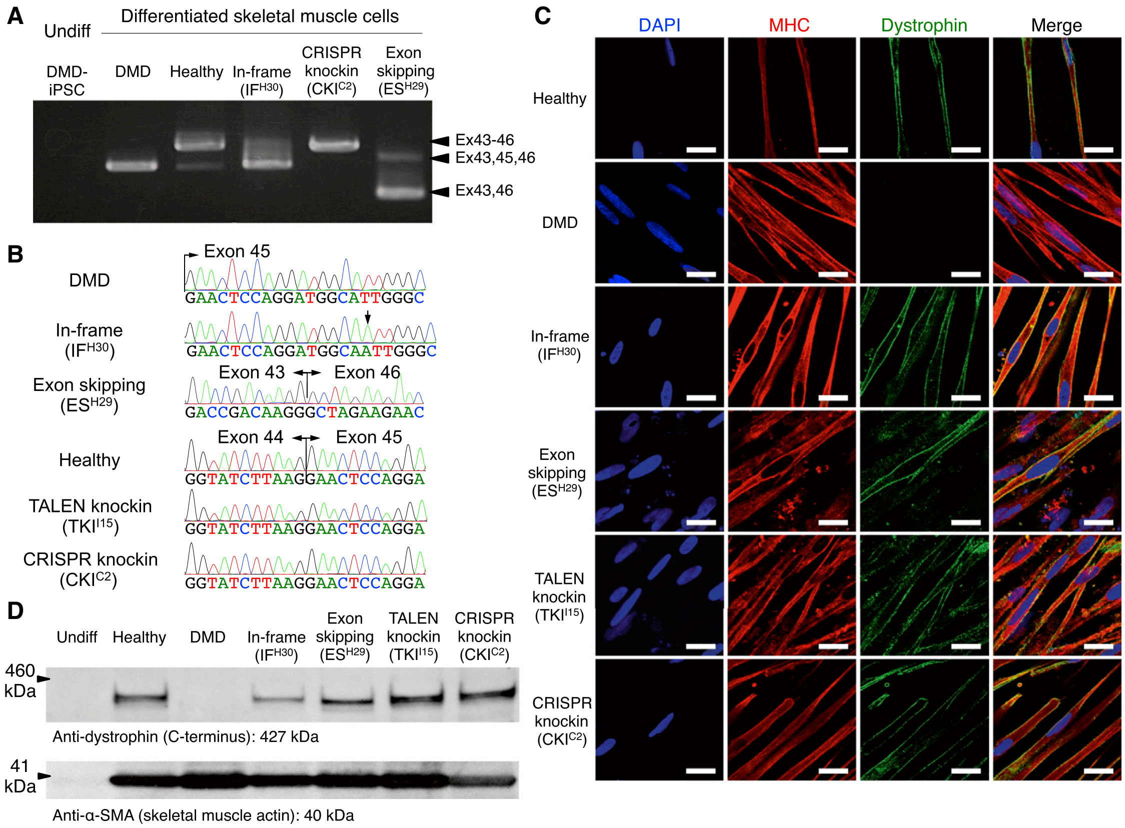

Figure 6. Restoration of the Dystrophin Protein in Differentiated Myogenic Cells

(A) RT-PCR analysis for dystrophin cDNA from iPSCs and cells differentiated from the corrected clones toward skeletal muscle lineage. The original DMD patient and an IF clone (IF^{H30}) with 1 bp insertion corresponded to the 452 and 453 bp PCR bands, respectively. The healthy control and knockin clones (TALEN-mediated TKI^{I15} and CRISPR-mediated CKI^{C2}) corresponded to the 600 bp bands; and the ES clone (ES^{H29}) corresponded to the 276 bp band.

(B) Sanger sequence analysis of dystrophin cDNAs from differentiated skeletal muscle cells. The IF clone (IF^{H30}) exhibited a 1 bp insertion (A, black arrow), the ES clone (ES^{H29}) exhibited the conjugation of exons 43 and 46 due to the skipping of exon 45, and knockin clones (TKI^{I15} and CKI^{C2}) exhibited the complete restoration of exon 44 in front of exon 45 as the healthy control.

(C) Immunofluorescence staining of skeletal muscle cells differentiated from the corrected clones. A z axis section of the confocal microscopy image shows submembrane localization of the dystrophin protein in the healthy control and all corrected clones, but not in the uncorrected original DMD iPSCs. The cells were stained by DAPI, a marker of skeletal differentiation (myosin heavy chain [MHC]), in red and an antibody that detects the rod domain of dystrophin (DYS1) in green. Scale bar, 50 μm.

(D) Western blot analysis to estimate the molecular weight of the dystrophin protein in the corrected clones. Expected molecular weight: 420 kDa for the reading-frame-corrected clone, 414 kDa for the exon-skipping clone, and 427 kDa for the exon 44 knockin clones and healthy control. An anti-dystrophin C terminus (amino acids 3661–3677) antibody was used to detect dystrophin protein, and an anti-α-SMA antibody was used as the sample loading control.

we did not detect a band in the original DMD clone. Together, our data indicate that genetically corrected iPSCs can express the dystrophin protein once they differentiate into myogenic cells.

DISCUSSION

Here, we have demonstrated that three distinct methods can correct the dystrophin gene: exon skipping, frameshifting, and exon knockin. All three approaches restored dystrophin protein expression in differentiated skeletal muscle cells. However, only the exon knockin approach restored the full-length dystrophin protein. We took advantage of the ability to expand iPSCs limitlessly and achieved a high percentage of knockin events by incorporating a drug selection system (up to 84% in the present study). Based on its precision and efficacy, we conclude that the knockin approach is preferable for correcting the dystrophin gene in iPSCs.

Regarding the nuclease specificity, both TALENs and CRISPR-sgRNA can bind to DNA despite a few base mismatches (Hsu et al., 2013). Therefore, it is critical to target a unique region in the genome with a minimal number of off-targets, as otherwise multiple targets may be cleaved. Several web-based programs can be used to search for off-target sites with a given target sequence region (e.g., CRISPR Design Tool [Hsu et al., 2013], Cas-OFFinder [Bae et al., 2014b], and E-CRISP [Heigwer et al., 2014]). However, these programs provide the predicted number of off-target sites only within a small region (typically ~500 bp) at any given time. Our unique *k*-mer approach allows the visualization of targetable regions in the entire genome, so users can select the targetable region(s) before checking the number of off-targets with other programs.

The risk of off-target mutagenesis is one of the most important obstacles to the therapeutic use of programmable nucleases. We performed a T7EI assay and amplicon deep sequencing to detect rare mutations at the target site, but did not detect an increased mutation rate from our results. To further assess the risk of target-sequence-independent off-target mutations, we employed combinations of rigorous genome-wide mutation analyses, such as the G-band for karyotyping, SNP array for detecting CNVs, and exome sequencing for searching SNVs and small indels. Since none of these methods alone sufficiently covers the large spectrum of mutations (from the single-nucleotide level to the chromosome level), it is important to combine several methods before applying gene therapy.

To achieve a therapeutic effect with genetically corrected iPSCs for an autologous ex vivo gene therapy approach, we must still overcome several hurdles, such as the successful transplantation of iPSC-derived myogenic cells. Since MYOD1-induced muscle cells from iPSCs have the ability to fuse (Goudenege et al., 2012; Tanaka et al., 2013), a corrected copy of the dystrophin gene may be able to contribute to an entire myofiber. Moreover, for long-term repopulation, the differentiation of iPSCs toward muscle progenitor cells (i.e., satellite cells) could be ideal for restoring damaged muscle in DMD patients (Darabi et al., 2012). In addition, an immunogenic response to the newly corrected gene product is possible (Mendell et al., 2010), although the response may be hindered by transient immunosuppression.

In summary, we have demonstrated the restoration of the dystrophin protein in patient-derived iPSCs by three different approaches. TALEN and CRISPR were equally effective and had minimal effects on off-target mutagenesis when they were targeted to a unique sequence region. Our efficient and precise correction method using TALEN and CRISPR technologies should provide a framework for future ex vivo gene therapy using patient-specific human iPSCs.

EXPERIMENTAL PROCEDURES

Generation of Integration-Free DMD iPSCs

DMD fibroblasts were derived from a DMD patient lacking exon 44 of the dystrophin gene after the subject provided written informed consent. The use of patient-derived samples and the genomic analysis were approved by the Ethics Committee of Kyoto University (no. 824 and no. G259, respectively). DMD fibroblasts were cultured in Dulbecco's modified Eagle's medium supplemented with 5% fetal bovine serum. To generate integration-free DMD iPSCs, we transfected 6×10^5 DMD fibroblasts with three episomal vectors (pCXLE-hOCT3/4-shp53-F, pCXLE-hSK, and pCXLE-hUL) by Neon electroporation (1650 V, 10 ms, 3 pulses) as described previously (Okita et al., 2011). The iPSC colonies that emerged were picked up and plated onto 24-well plates with feeders on day 31 and then expanded.

To screen for iPSC clones that were negative for residual episomal vectors, iPSC pellets were lysed with 500 μl of lysis solution (200 μg ml^{-1} proteinase K) at 55°C for 3–16 hr. Genomic DNA was purified by phenol-chloroform extraction and ethanol precipitation, and then used for quantitative PCR analyses using the primers listed in Table S6. An episomal plasmid was used to determine the standard curve, and DMD-iPSC clones with fewer than 0.01 copies were deemed integration-free iPSC clones. The original DMD-iPSC #1 (clone ID: CiRA00111) and the corrected clones, including the IF clone IF^{D28} (clone ID: CiRA00111-IF-D28), ES clone ES^{H19} (clone ID: CiRA00111-ES-H19), TALEN-mediated knockin clone TKI^{I15} (clone ID: CiRA00111-TKI-I15), and CRISPR-mediated knockin clone CKI^{C2} (clone ID: CiRA00111-CKI-C2), will be available from the RIKEN BRC Cell Bank (cell no. HPS0383-HPS0387).

Unique *k*-mer Sequence Database

To identify unique sequence regions and avoid repeated sequences in the human genome, we generated all possible combinations of small *k*-mer sequences (≤16 bp) by a custom Perl script. We then mapped the *k*-mer sequences onto the human genome (hg19) using Bowtie (Langmead et al., 2009), with no mismatch allowed. Only uniquely mapped *k*-mer sequences were pooled as the data set. To visually show the stack of unique *k*-mer sequences, the mapping data were converted to the BEDGRAPH format by genomeCoverageBed and then converted to TDF format by igvtools or to the bigWig format by bedGraphToBigWig. The unique k-mer sequence (Unik) database will be available on our website (https://apps.cira.kyoto-u.ac.jp/igeats).

Transfection of TALEN and CRISPR into Human iPSCs

Target iPSCs were pretreated with a ROCK inhibitor (Y-27632; Sigma) at 10 μM for at least 1 hr before electroporation. The cells were washed with PBS and treated with CTK solution for 1–3 min at 37°C to remove feeders and then were washed with PBS twice. Next, the iPSCs were further dissociated into single cells by a 0.25% Trypsin solution for 5–8 min at 37°C and were neutralized with culture medium containing 10% fetal bovine serum. We electroporated 10 μg of nuclease-expressing plasmids (TALENs: 5 μg left and 5 μg right; CRISPR: 5 μg Cas9 and 5 μg sgRNA) and 5 μg donor plasmid (if applicable) into 1×10^6 cells using a NEPA 21 electroporator (poring pulse: pulse voltage, 125 V; pulse

width, 5 ms; pulse number, 2; Negagene). Cells were plated onto one well of a six-well plate with feeders in the presence of 10 μM Y-27632 for 1–2 days.

Analysis of Indel Patterns by Deep Sequencing

The dystrophin gene target region was PCR amplified with barcoding primers (DMD-MiSeq-Rd1-fwd1 and DMD-MiSeq-Rd2-rev1) and then adaptor primers (Multiplex P5 fwd and Multiplex P7 rev) using a high-fidelity PCR enzyme. The resultant PCR products were gel purified and quantified by a Qubit 2.0 Fluorometer (Life Technologies) and the KAPA Library Quantification Kit for Illumina (KAPA Biosystems). Each DNA sample was adjusted to 2 nM and denatured by 0.2 N NaOH solution for 5 min. The samples were further diluted to 12 pM, mixed with 4 pM of PhiX spike-in DNA, and run on MiSeq using the MiSeq Reagent Kit v2 for 2 × 150 bp sequencing. The generated FASTQ sequence files were filtered by the fastq_quality_filter program from the FASTX-Toolkit to remove low-quality sequencing reads. After removal of the PhiX sequences, the remaining sequencing reads were split based on the barcode indices by the fastx_barcode_splitter program. The resultant reads were mapped to the target sequences by BWA, and the mutation patterns were extracted from the CIGAR code and MD tag.

Frameshift Screening without a Template Donor

Genomic DNAs from the transfected iPSCs were analyzed by the T7EI assay and restriction enzyme (XcmI) sensitivity assay to monitor the efficiency of the nuclease-mediated mutagenesis. Then the cells were dissociated into single cells and diluted to 200–500 cells per 10 cm dish with feeders. The subclonal colonies that emerged were picked on days 11–13 after reseeding. From the genomic PCR sequencing, the indels at dystrophin exon 45 with the ($3n + 2$) bp deletion or ($3n + 1$) bp insertion (where n is a nonnegative integer) were further expanded for later experiments.

TALEN- or CRISPR-Mediated Exon 44 Knockin

For the knockin experiment, 5 μg of donor vector was cotransfected with TALEN expression vectors (5 μg for left TALEN and 5 μg for right TALEN) or Cas9 and sgRNA expression vectors (5 μg for Cas9 and 5 μg for sgRNA) using NEPA 21 as described above. Hygromycin B (25 μg ml^{-1}; Invitrogen) selection was applied after iPSC colonies were recovered (4–5 days after transfection). The resulting hygromycin-resistant colonies were dissociated into single cells and plated at 200–500 cells per 10 cm dish with feeders. Each subclone was screened by genomic PCR (with P1-P2 primer pairs, amplifying a fragment from upstream of the 3′ arm to the EF1α-promoter, and P3-P4 primer pairs, amplifying a fragment from exon 44 to downstream of the 5′ arm). Homologous recombinants were further verified by Southern blot analysis using EcoRI and probe intron 45. After establishing the single-copy knockin clones, we electroporated the cells with 10 μg of the *Cre* expression vector pCXW-Cre-puro using NEPA 21. Clone isolation was carried out as described above, and excision of the hygromycin-selection cassette was confirmed by PCR screening with primers P1 and P4 and Southern blot analysis with EcoRI digestion and probe intron 45.

Skeletal Muscle Differentiation by Dox-Inducible MYOD1

The induction of skeletal muscle differentiation from iPSCs was described previously (Tanaka et al., 2013). Briefly, a Dox-inducible MYOD1-expressing *piggyBac* vector, PB-TetO-MyoD, was coelectroporated with the *piggyBac* transposase vector PBaseII (Matsui et al., 2014) using NEPA 21 (125 V, 5 ms). G418 (Calbiochem) selection (100 μg ml^{-1}) was applied to select stable PB-TetO-MyoD clones. Among the several G418-resistant clones, we screened for clones with a high mCherry induction rate upon addition of 1 μg ml^{-1} doxycycline (Funakoshi). Successful differentiation was confirmed by a spindle-shape-like morphology and immunocytochemical staining with myosin heavy chain (MHC) and α-skeletal muscle actin (α-SMA) antibodies on day 9 postdifferentiation.

ACCESSION NUMBERS

The plasmid DNAs used in this study are available from Addgene (https://www.addgene.org/) under accession numbers 60599–60605. DMD-patient-derived iPSCs and genetically corrected subclones are available from the RIKEN BRC Cell Bank (http://www.brc.riken.jp/lab/cell/english/) under accession numbers HPS0383–HPS0387.

SUPPLEMENTAL INFORMATION

Supplemental Information includes Supplemental Experimental Procedures, six figures, and six tables and can be found with this article online at http://dx.doi.org/10.1016/j.stemcr.2014.10.013.

AUTHOR CONTRIBUTIONS

H.L.L., S.Y., and A.H. conceived and designed the project. H.L.L., N.F., N.S., S.S., T.O., N.A, A.W., and A.H. performed the experiments. T.S. and T.Y. provided the TALEN construction platforms. M.T. constructed the website. H.S. provided the skeletal muscle differentiation protocol. H.L.L. and A.H. interpreted the data and wrote the manuscript.

ACKNOWLEDGMENTS

We thank the anonymous DMD patient and his family for kindly providing the biopsy. We also thank Dr. Megumu K. Saito and Takayuki Tanaka for preparing the cell samples; Dr. Kazutoshi Takahashi, Dr. Yusuke Echigoya, and Emi Shoji for their technical advice; Dr. Peter Karagiannis for a critical reading of the manuscript; Dr. Knut Woltjen and Dr. Keisuke Okita for providing vectors; Yumie Tokunaga for the SNP array analysis; and Osamu Ohta for computational advice. This research was supported in part by JSPS KAKENHI, JST PRESTO, JST Yamanaka iPS Cell Special Project, and the JST Research Center Network for Realization of Regenerative Medicine. H.L.L. is a recipient of a JSPS DC1 fellowship. S.Y. is a scientific advisor of iPS Academia Japan without salary.

Received: September 4, 2014
Revised: October 24, 2014
Accepted: October 24, 2014
Published: November 26, 2014

REFERENCES

Aartsma-Rus, A., Fokkema, I., Verschuuren, J., Ginjaar, I., van Deutekom, J., van Ommen, G.J., and den Dunnen, J.T. (2009). Theoretic applicability of antisense-mediated exon skipping for Duchenne muscular dystrophy mutations. Hum. Mutat. *30*, 293–299.

Amps, K., Andrews, P.W., Anyfantis, G., Armstrong, L., Avery, S., Baharvand, H., Baker, J., Baker, D., Munoz, M.B., Beil, S., et al.; International Stem Cell Initiative (2011). Screening ethnically diverse human embryonic stem cells identifies a chromosome 20 minimal amplicon conferring growth advantage. Nat. Biotechnol. *29*, 1132–1144.

Bae, S., Kweon, J., Kim, H.S., and Kim, J.S. (2014a). Microhomology-based choice of Cas9 nuclease target sites. Nat. Methods *11*, 705–706.

Bae, S., Park, J., and Kim, J.S. (2014b). Cas-OFFinder: a fast and versatile algorithm that searches for potential off-target sites of Cas9 RNA-guided endonucleases. Bioinformatics *30*, 1473–1475.

Canver, M., Bauer, D., Dass, A., Yien, Y., Chung, J., Masuda, T., Maeda, T., Paw, B., and Orkin, S. (2014). Characterization of genomic deletion efficiency mediated by clustered regularly interspaced palindromic repeats (CRISPR)/Cas9 nuclease system in mammalian cells. J. Biol. Chem. *289*, 21312–21324.

Choi, S.M., Kim, Y., Shim, J.S., Park, J.T., Wang, R.H., Leach, S.D., Liu, J.O., Deng, C., Ye, Z., and Jang, Y.Y. (2013). Efficient drug screening and gene correction for treating liver disease using patient-specific stem cells. Hepatology *57*, 2458–2468.

Cong, L., Ran, F.A., Cox, D., Lin, S., Barretto, R., Habib, N., Hsu, P.D., Wu, X., Jiang, W., Marraffini, L.A., and Zhang, F. (2013). Multiplex genome engineering using CRISPR/Cas systems. Science *339*, 819–823.

Darabi, R., Arpke, R.W., Irion, S., Dimos, J.T., Grskovic, M., Kyba, M., and Perlingeiro, R.C. (2012). Human ES- and iPS-derived myogenic progenitors restore DYSTROPHIN and improve contractility upon transplantation in dystrophic mice. Cell Stem Cell *10*, 610–619.

Ding, Q., Lee, Y.K., Schaefer, E.A., Peters, D.T., Veres, A., Kim, K., Kuperwasser, N., Motola, D.L., Meissner, T.B., Hendriks, W.T., et al. (2013a). A TALEN genome-editing system for generating human stem cell-based disease models. Cell Stem Cell *12*, 238–251.

Ding, Q., Regan, S.N., Xia, Y., Oostrom, L.A., Cowan, C.A., and Musunuru, K. (2013b). Enhanced efficiency of human pluripotent stem cell genome editing through replacing TALENs with CRISPRs. Cell Stem Cell *12*, 393–394.

Filareto, A., Parker, S., Darabi, R., Borges, L., Iacovino, M., Schaaf, T., Mayerhofer, T., Chamberlain, J.S., Ervasti, J.M., McIvor, R.S., et al. (2013). An ex vivo gene therapy approach to treat muscular dystrophy using inducible pluripotent stem cells. Nat. Commun. *4*, 1549.

Fu, Y., Foden, J.A., Khayter, C., Maeder, M.L., Reyon, D., Joung, J.K., and Sander, J.D. (2013). High-frequency off-target mutagenesis induced by CRISPR-Cas nucleases in human cells. Nat. Biotechnol. *31*, 822–826.

Goudenege, S., Lebel, C., Huot, N.B., Dufour, C., Fujii, I., Gekas, J., Rousseau, J., and Tremblay, J.P. (2012). Myoblasts derived from normal hESCs and dystrophic hiPSCs efficiently fuse with existing muscle fibers following transplantation. Mol. Ther. *20*, 2153–2167.

Heigwer, F., Kerr, G., and Boutros, M. (2014). E-CRISP: fast CRISPR target site identification. Nat. Methods *11*, 122–123.

Hockemeyer, D., Wang, H., Kiani, S., Lai, C.S., Gao, Q., Cassady, J.P., Cost, G.J., Zhang, L., Santiago, Y., Miller, J.C., et al. (2011). Genetic engineering of human pluripotent cells using TALE nucleases. Nat. Biotechnol. *29*, 731–734.

Hotta, A., Cheung, A.Y., Farra, N., Vijayaragavan, K., Séguin, C.A., Draper, J.S., Pasceri, P., Maksakova, I.A., Mager, D.L., Rossant, J., et al. (2009). Isolation of human iPS cells using EOS lentiviral vectors to select for pluripotency. Nat. Methods *6*, 370–376.

Hsu, P.D., Scott, D.A., Weinstein, J.A., Ran, F.A., Konermann, S., Agarwala, V., Li, Y., Fine, E.J., Wu, X., Shalem, O., et al. (2013). DNA targeting specificity of RNA-guided Cas9 nucleases. Nat. Biotechnol. *31*, 827–832.

Koehler, R., Issac, H., Cloonan, N., and Grimmond, S.M. (2011). The uniqueome: a mappability resource for short-tag sequencing. Bioinformatics *27*, 272–274.

Langmead, B., Schatz, M.C., Lin, J., Pop, M., and Salzberg, S.L. (2009). Searching for SNPs with cloud computing. Genome Biol. *10*, R134.

Laurent, L.C., Ulitsky, I., Slavin, I., Tran, H., Schork, A., Morey, R., Lynch, C., Harness, J.V., Lee, S., Barrero, M.J., et al. (2011). Dynamic changes in the copy number of pluripotency and cell proliferation genes in human ESCs and iPSCs during reprogramming and time in culture. Cell Stem Cell *8*, 106–118.

Li, H.L., Nakano, T., and Hotta, A. (2014). Genetic correction using engineered nucleases for gene therapy applications. Dev. Growth Differ. *56*, 63–77.

Lin, Y., Cradick, T.J., Brown, M.T., Deshmukh, H., Ranjan, P., Sarode, N., Wile, B.M., Vertino, P.M., Stewart, F.J., and Bao, G. (2014). CRISPR/Cas9 systems have off-target activity with insertions or deletions between target DNA and guide RNA sequences. Nucleic Acids Res. *42*, 7473–7485.

Ma, N., Liao, B., Zhang, H., Wang, L., Shan, Y., Xue, Y., Huang, K., Chen, S., Zhou, X., Chen, Y., et al. (2013). Transcription activator-like effector nuclease (TALEN)-mediated gene correction in integration-free β-thalassemia induced pluripotent stem cells. J. Biol. Chem. *288*, 34671–34679.

Maetzel, D., Sarkar, S., Wang, H., Abi-Mosleh, L., Xu, P., Cheng, A.W., Gao, Q., Mitalipova, M., and Jaenisch, R. (2014). Genetic and chemical correction of cholesterol accumulation and impaired autophagy in hepatic and neural cells derived from Niemann-Pick Type C patient-specific iPS cells. Stem Cell Rep. *2*, 866–880.

Mali, P., Yang, L., Esvelt, K.M., Aach, J., Guell, M., DiCarlo, J.E., Norville, J.E., and Church, G.M. (2013). RNA-guided human genome engineering via Cas9. Science *339*, 823–826.

Matsui, H., Fujimoto, N., Sasakawa, N., Ohinata, Y., Shima, M., Yamanaka, S., Sugimoto, M., and Hotta, A. (2014). Delivery of full-length factor VIII using a piggyBac transposon vector to correct a mouse model of hemophilia A. PLoS ONE *9*, e104957.

Mendell, J.R., Campbell, K., Rodino-Klapac, L., Sahenk, Z., Shilling, C., Lewis, S., Bowles, D., Gray, S., Li, C., Galloway, G., et al. (2010).

Dystrophin immunity in Duchenne's muscular dystrophy. N. Engl. J. Med. *363*, 1429–1437.

Okada, T., and Takeda, S. (2013). Current challenges and future directions in recombinant AAV-mediated gene therapy of Duchenne muscular dystrophy. Pharmaceuticals (Basel) *6*, 813–836.

Okita, K., Matsumura, Y., Sato, Y., Okada, A., Morizane, A., Okamoto, S., Hong, H., Nakagawa, M., Tanabe, K., Tezuka, K., et al. (2011). A more efficient method to generate integration-free human iPS cells. Nat. Methods *8*, 409–412.

Osborn, M.J., Starker, C.G., McElroy, A.N., Webber, B.R., Riddle, M.J., Xia, L., DeFeo, A.P., Gabriel, R., Schmidt, M., von Kalle, C., et al. (2013). TALEN-based gene correction for epidermolysis bullosa. Mol. Ther. *21*, 1151–1159.

Ousterout, D.G., Perez-Pinera, P., Thakore, P.I., Kabadi, A.M., Brown, M.T., Qin, X., Fedrigo, O., Mouly, V., Tremblay, J.P., and Gersbach, C.A. (2013). Reading frame correction by targeted genome editing restores dystrophin expression in cells from Duchenne muscular dystrophy patients. Mol. Ther. *21*, 1718–1726.

Park, I.H., Arora, N., Huo, H., Maherali, N., Ahfeldt, T., Shimamura, A., Lensch, M.W., Cowan, C., Hochedlinger, K., and Daley, G.Q. (2008). Disease-specific induced pluripotent stem cells. Cell *134*, 877–886.

Pichavant, C., Aartsma-Rus, A., Clemens, P.R., Davies, K.E., Dickson, G., Takeda, S., Wilton, S.D., Wolff, J.A., Wooddell, C.I., Xiao, X., and Tremblay, J.P. (2011). Current status of pharmaceutical and genetic therapeutic approaches to treat DMD. Mol. Ther. *19*, 830–840.

Popplewell, L., Koo, T., Leclerc, X., Duclert, A., Mamchaoui, K., Gouble, A., Mouly, V., Voit, T., Pâques, F., Cédrone, F., et al. (2013). Gene correction of a duchenne muscular dystrophy mutation by meganuclease-enhanced exon knock-in. Hum. Gene Ther. *24*, 692–701.

Rousseau, J., Chapdelaine, P., Boisvert, S., Almeida, L.P., Corbeil, J., Montpetit, A., and Tremblay, J.P. (2011). Endonucleases: tools to correct the dystrophin gene. J. Gene Med. *13*, 522–537.

Sakuma, T., Hosoi, S., Woltjen, K., Suzuki, K., Kashiwagi, K., Wada, H., Ochiai, H., Miyamoto, T., Kawai, N., Sasakura, Y., et al. (2013a). Efficient TALEN construction and evaluation methods for human cell and animal applications. Genes Cells *18*, 315–326.

Sakuma, T., Ochiai, H., Kaneko, T., Mashimo, T., Tokumasu, D., Sakane, Y., Suzuki, K., Miyamoto, T., Sakamoto, N., Matsuura, S., and Yamamoto, T. (2013b). Repeating pattern of non-RVD variations in DNA-binding modules enhances TALEN activity. Sci. Rep. *3*, 3379.

Sebastiano, V., Maeder, M.L., Angstman, J.F., Haddad, B., Khayter, C., Yeo, D.T., Goodwin, M.J., Hawkins, J.S., Ramirez, C.L., Batista, L.F., et al. (2011). In situ genetic correction of the sickle cell anemia mutation in human induced pluripotent stem cells using engineered zinc finger nucleases. Stem Cells *29*, 1717–1726.

Smith, C., Gore, A., Yan, W., Abalde-Atristain, L., Li, Z., He, C., Wang, Y., Brodsky, R.A., Zhang, K., Cheng, L., and Ye, Z. (2014). Whole-genome sequencing analysis reveals high specificity of CRISPR/Cas9 and TALEN-based genome editing in human iPSCs. Cell Stem Cell *15*, 12–13.

Soldner, F., Laganière, J., Cheng, A.W., Hockemeyer, D., Gao, Q., Alagappan, R., Khurana, V., Golbe, L.I., Myers, R.H., Lindquist, S., et al. (2011). Generation of isogenic pluripotent stem cells differing exclusively at two early onset Parkinson point mutations. Cell *146*, 318–331.

Sugiura, M., Kasama, Y., Araki, R., Hoki, Y., Sunayama, M., Uda, M., Nakamura, M., Ando, S., and Abe, M. (2014). Induced pluripotent stem cell generation-associated point mutations arise during the initial stages of the conversion of these cells. Stem Cell Rep. *2*, 52–63.

Suzuki, K., Yu, C., Qu, J., Li, M., Yao, X., Yuan, T., Goebl, A., Tang, S., Ren, R., Aizawa, E., et al. (2014). Targeted gene correction minimally impacts whole-genome mutational load in human-disease-specific induced pluripotent stem cell clones. Cell Stem Cell *15*, 31–36.

Takahashi, K., Tanabe, K., Ohnuki, M., Narita, M., Ichisaka, T., Tomoda, K., and Yamanaka, S. (2007). Induction of pluripotent stem cells from adult human fibroblasts by defined factors. Cell *131*, 861–872.

Tanaka, A., Woltjen, K., Miyake, K., Hotta, A., Ikeya, M., Yamamoto, T., Nishino, T., Shoji, E., Sehara-Fujisawa, A., Manabe, Y., et al. (2013). Efficient and reproducible myogenic differentiation from human iPS cells: prospects for modeling Miyoshi Myopathy in vitro. PLoS ONE *8*, e61540.

Tuffery-Giraud, S., Béroud, C., Leturcq, F., Yaou, R.B., Hamroun, D., Michel-Calemard, L., Moizard, M.P., Bernard, R., Cossée, M., Boisseau, P., et al. (2009). Genotype-phenotype analysis in 2,405 patients with a dystrophinopathy using the UMD-DMD database: a model of nationwide knowledgebase. Hum. Mutat. *30*, 934–945.

Veres, A., Gosis, B.S., Ding, Q., Collins, R., Ragavendran, A., Brand, H., Erdin, S., Talkowski, M.E., and Musunuru, K. (2014). Low incidence of off-target mutations in individual CRISPR-Cas9 and TALEN targeted human stem cell clones detected by whole-genome sequencing. Cell Stem Cell *15*, 27–30.

Ye, L., Wang, J., Beyer, A.I., Teque, F., Cradick, T.J., Qi, Z., Chang, J.C., Bao, G., Muench, M.O., Yu, J., et al. (2014). Seamless modification of wild-type induced pluripotent stem cells to the natural CCR5Δ32 mutation confers resistance to HIV infection. Proc. Natl. Acad. Sci. USA *111*, 9591–9596.

Zou, J., Mali, P., Huang, X., Dowey, S.N., and Cheng, L. (2011a). Site-specific gene correction of a point mutation in human iPS cells derived from an adult patient with sickle cell disease. Blood *118*, 4599–4608.

Zou, J., Sweeney, C.L., Chou, B.K., Choi, U., Pan, J., Wang, H., Dowey, S.N., Cheng, L., and Malech, H.L. (2011b). Oxidase-deficient neutrophils from X-linked chronic granulomatous disease iPS cells: functional correction by zinc finger nuclease-mediated safe harbor targeting. Blood *117*, 5561–5572.

Stem Cell Reports, Volume *4*

Supplemental Information

Precise Correction of the Dystrophin Gene in Duchenne Muscular Dystrophy Patient Induced Pluripotent Stem Cells by TALEN and CRISPR-Cas9

Hongmei Lisa Li, Naoko Fujimoto, Noriko Sasakawa, Saya Shirai, Tokiko Ohkame, Tetsushi Sakuma, Michihiro Tanaka, Naoki Amano, Akira Watanabe, Hidetoshi Sakurai, Takashi Yamamoto, Shinya Yamanaka, and Akitsu Hotta

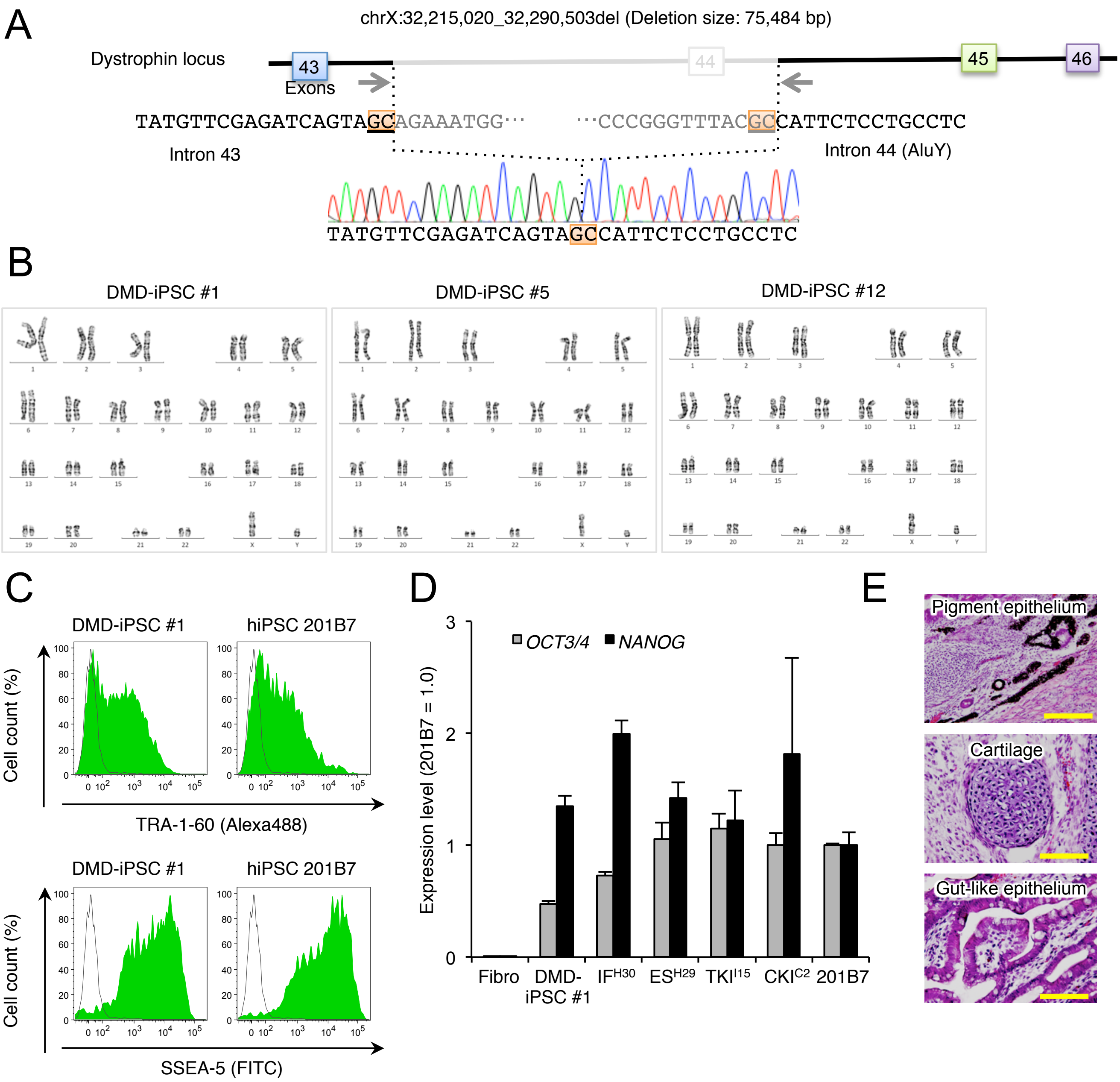

Figure S1. Establishment of DMD-iPSCs, Related to Figures 2-6.

(A) Breakpoint analysis of the copy number loss of exon 44 in the dystrophin gene in the genome of a DMD patient who lacked exon 44. Multiple PCRs and Sanger sequences identified the loss of a 75 kbp region including exon 44. The orange box in the lower panel indicates the micro-homology sequence "GC" found at the breakpoint. (B) iPS cell lines generated by episomal vectors maintained normal karyotypes. (C) Flow cytometry analysis showed that DMD-iPSC clone #1 was positive for pluripotency markers TRA-1-60 and SSEA-5, equivalent to the control hiPSC 201B7 clone. (D) qRT-PCR analysis for *OCT3/4* and *NANOG* in the DMD-iPSC #1 clone and the derivative clones. The data were normalized to those of the control hiPSC line, 201B7. The values represent mean ± SD among technical replicates (n = 3). (E) A histological examination of a teratoma from DMD-iPSC #1 showed the formation of tissue structures that correspond to the three germ layers: pigmented epithelium (ectoderm), cartilage (mesoderm) and gut-like epithelium (endoderm). Scale bar, 100 μm.

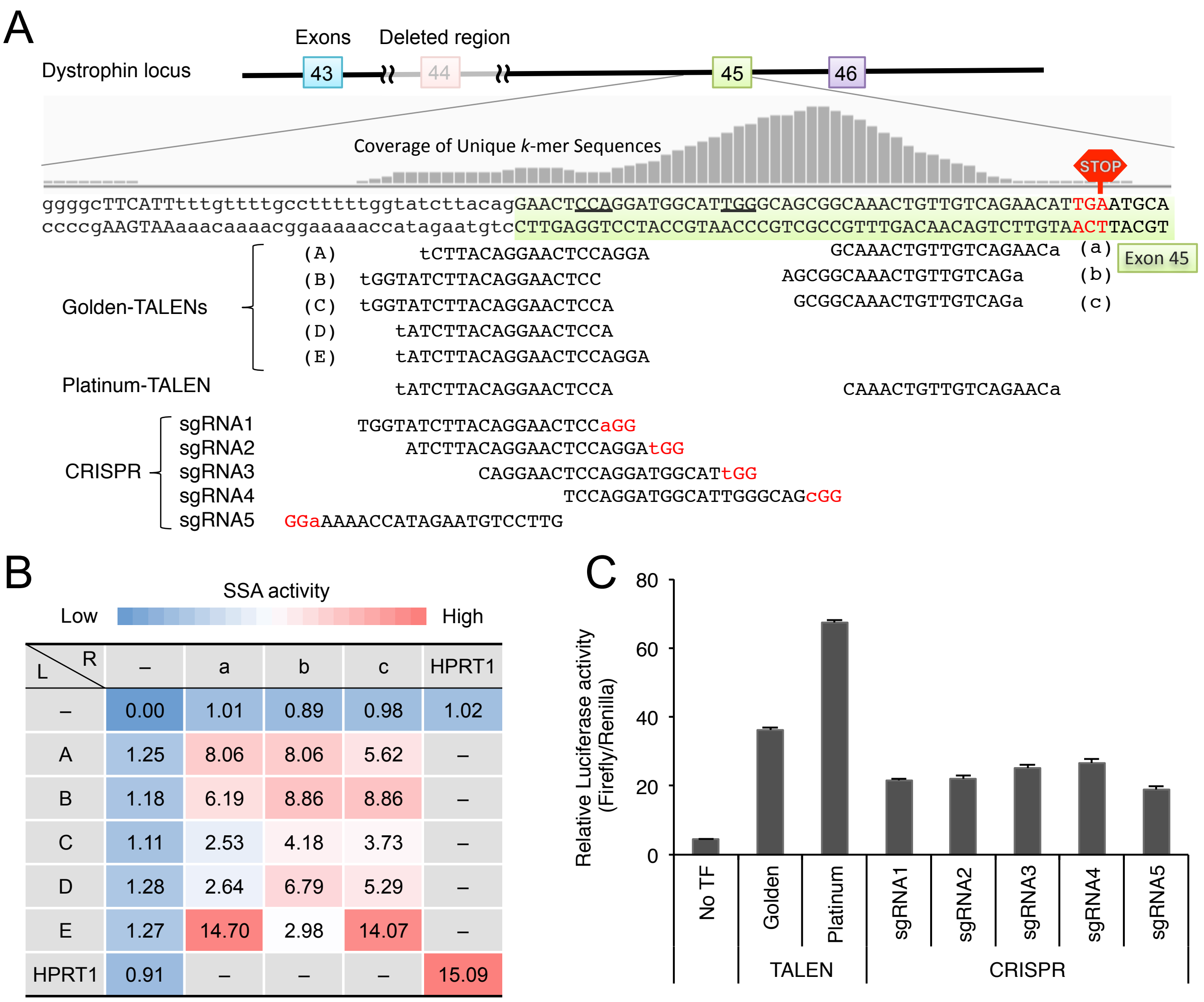

Figure S2. Screening for the most active TALENs and CRIPSR-sgRNAs, Related to Figure 2.

(A) The target sites of the nucleases used in this study, including TALEN pairs and CRISPR-sgRNAs, at exon 45 of the dystrophin gene. Golden-TALENs were constructed with the Golden-Gate assembly method, and Platinum-TALEN was constructed with the Platinum-Gate assembly method with some non-RVD variation. CRISPR-sgRNA1-4 target the sense strand, whereas sgRNA5 targets the antisense strand. (B) The activities of Golden-TALENs were analyzed by a SSA (single-strand annealing) assay in HEK293T cells (n = 3). Left TALENs (A-E) and right TALENs (a-c) were co-transfected with a luciferase-expressing vector, where the luciferase gene was separated from the exon 45 target site. Among the pairs of combinations tested, the E/a and E/c pairs showed highest SSA activity, a result similar to that of the positive control TALEN pair targeting the HPRT1 gene. (C) The SSA activities of CRISPR-sgRNAs in HEK293T cells. Golden-TALEN (E/a pair) and the Platinum-TALEN pair were used as positive controls. Each of the five CRISPR-sgRNAs was transfected with human-codon optimized wild-type Cas9. The values represent mean ± SD among technical replicates (n = 3).

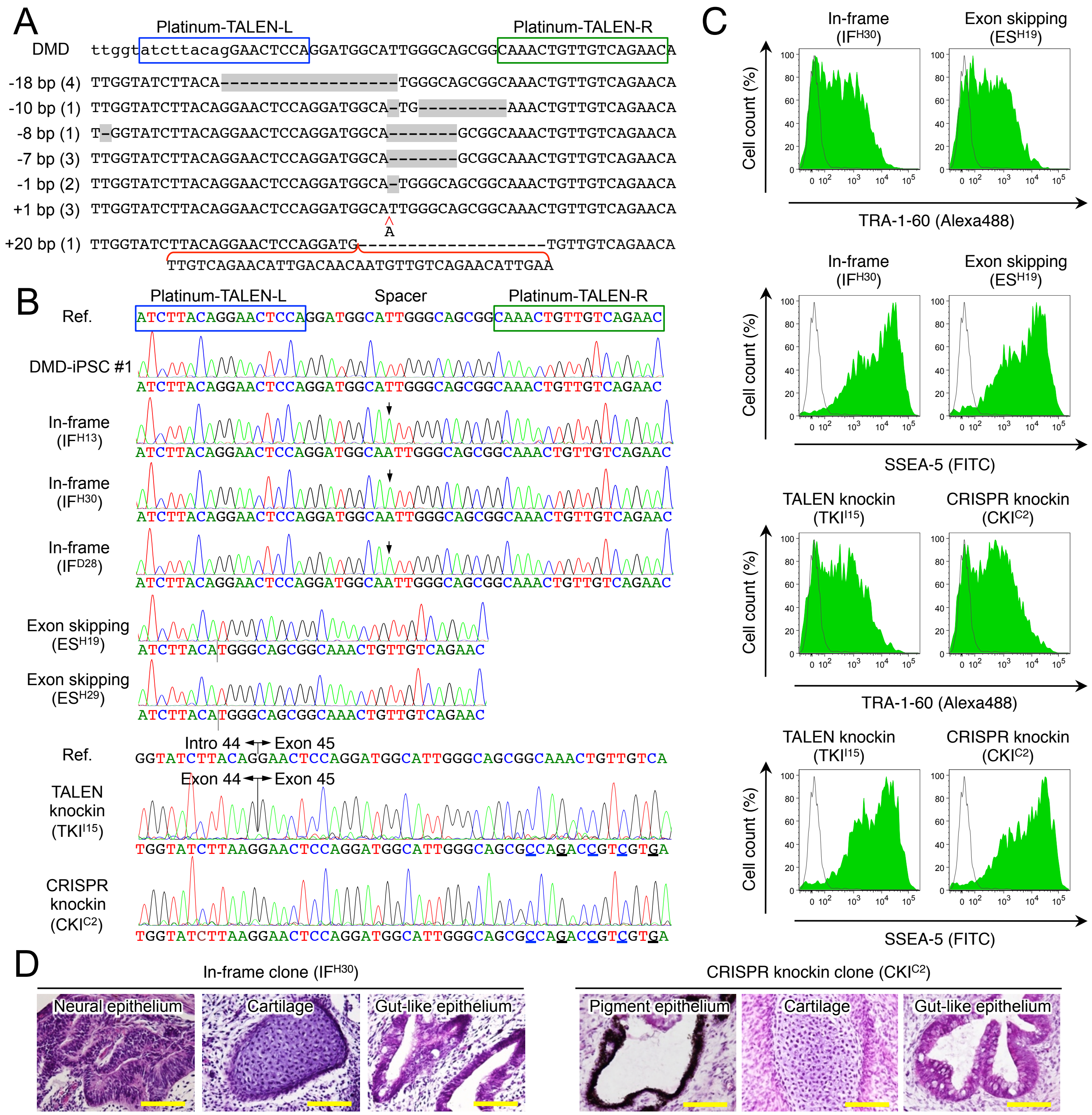

Figure S3. Confirmation of the corrected DMD-iPS cell clones, Related to Figures 3, 4.

(A) Indel patterns induced by Platinum-TALEN. The sizes of the indels are shown on the left, and the number of clones for each indel are indicated in parentheses. Clones with a 1 bp insertion are in-frame clones, and those with the 18 bp deletion are exon 45 skipping clones. Other indels did not restore the reading frame. (B) Sanger sequence electrograms confirmed corresponding clones with the 1 bp insertion (IF^{H13}, IF^{H30} and IF^{D28}), exon skipping clones with the 18 bp deletion (ES^{H19} and ES^{H29}) and knockin clones with the genomic exon 44-45 junction (TKI^{I15} and CKI^{C2}). The original DMD-iPSC sequence is shown at the top and middle for reference. Black arrow indicates the 1 bp insertion. Underlined sequence in knockin clones indicates 5 silent mutations to prevent the re-cutting. (C) The expression of pluripotency markers, TRA-1-60 and SSEA-5, in the in-frame, exon skipping and exon 44 knockin clones as assessed by flow cytometry. (D) Teratoma assay of the corrected DMD-iPS clones (IF^{H30} and CKI^{C2}) showed the formation of tissue structures that correspond to the three germ layers. Scale bar, 100 μm.

A

		Position	Sequence
On-target	Platinum-TALEN-Left	chrX:31986626-31986642	tATCTTACAGGAACTCCA
	sgRNA1	chrX:31986622-31986644	TGGTATCTTACAGGAACTCCAGG
Off-target	OT1	chr14:52643730-52643776	TGGTATCTTAaAGaAACTCCAGG
	OT2	chr9:33384854-33384900	TGGgtTCTTACAGGAgCTCCAGG
	OT3	chr12:91808790-91808836	TGGTcTCTTACAGGAACTCaAGa
	OT4	chr4:100047228-100047274	aGGTATCTTAgAGGAACTCCAtG
	OT5	chr2:66263917-66263963	TGGgATCTaACAGtAACTCCAGG

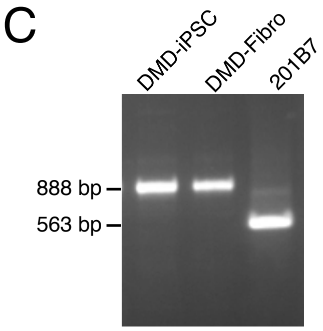

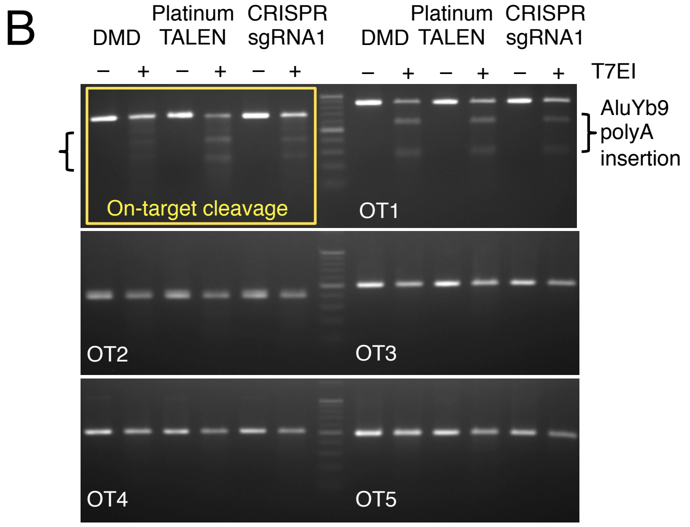

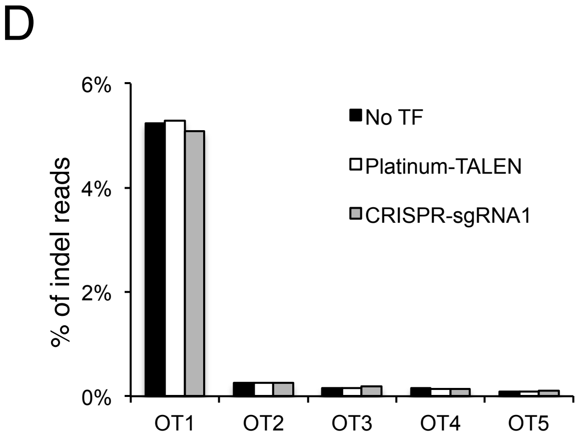

Figure S4. No severe mutagenesis observed at the predicted off-target sites, Related to Figure 5.

(A) The predicted off-target (OT) sites allowed up to three mismatches for CRISPR-sgRNA1. Red characters indicate mismatches compared to on-target sgRNA1. (B) The predicted off-target loci were amplified by PCR and analyzed by a T7EI assay. The yellow box shows successful detection of the on-target mutagenesis by our T7EI assay. OT2-5 sites showed no cleavage at the off-target sites except for OT1. Because the PCR amplicon size for OT1 was larger than expected and because we observed cleaved bands even in the control (No TF), we sequenced the OT1 PCR fragment and found the homozygous insertion of AluYb9 in the middle of the OT1 site. (C) Genomic PCR analysis confirmed the insertion of the AluYb9 at OT1 in original fibroblasts derived from the DMD patient, but not in a control human iPSC clone (201B7). (D) Indel mutations at the off-target sites were assessed by amplicon deep sequencing. The OT1 site suffered from a high indel ratio due to the poly A region of the AluYb9 element. No TF: no transfection DMD-iPSCs.

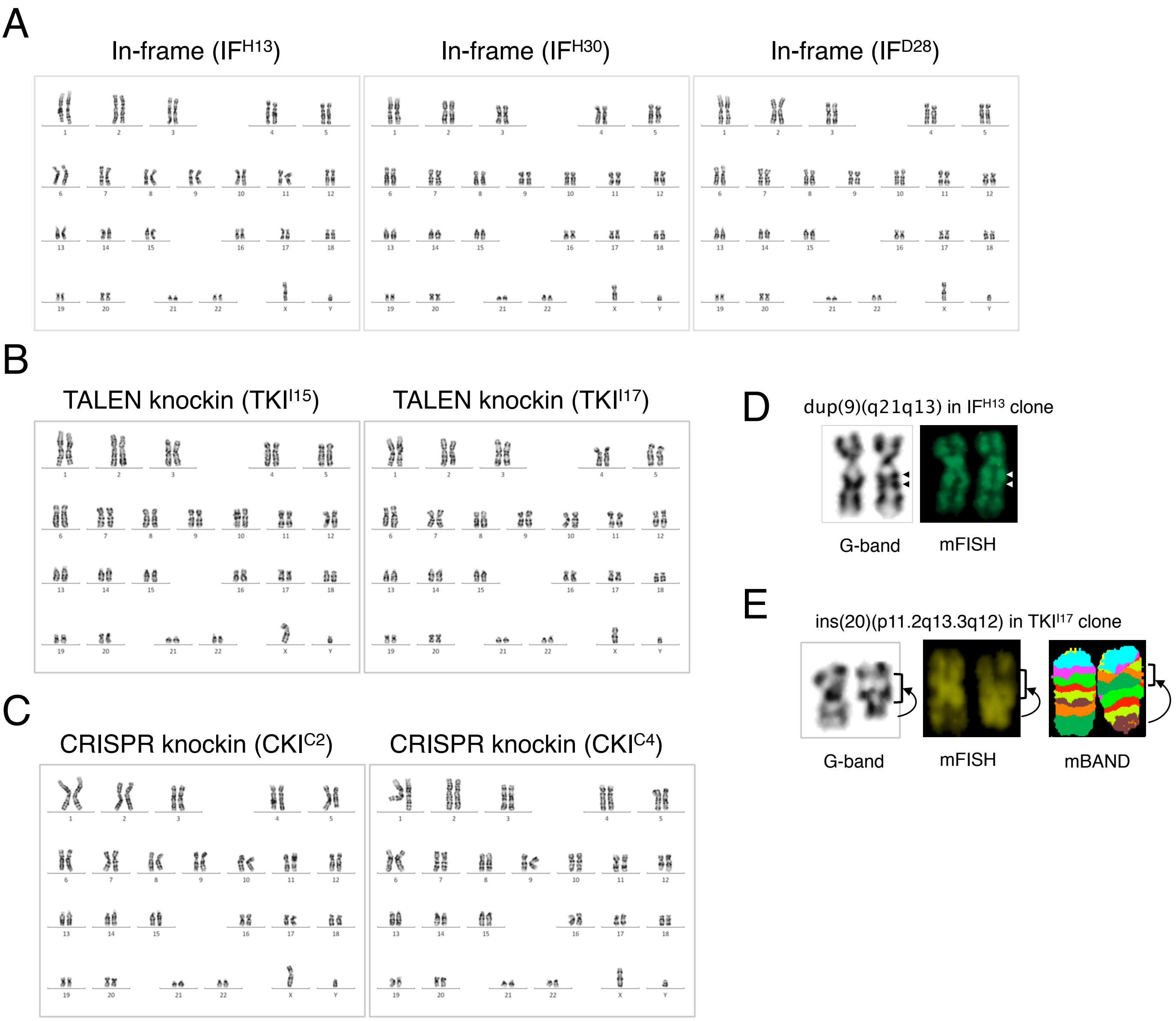

Figure S5. Karyotyping analysis of the corrected clones, Related to Figure 5A.

(A) (B) and (C) High-resolution G-banding analysis revealed that most cells showed a normal karyotype with corrected clones, in-frame clones (IF^{H13}, IF^{H30} and IF^{D28}) (A), TALEN-mediated knockin clones (TKI^{I15} and TKI^{I17}) (B) and CRISPR-mediated knockin clones (CKI^{C2} and CKI^{C4}) (C). (D) G-banding and mFISH images showed suspected duplication (two arrowheads) at chromosome 9 in the IF^{H13} clone (5 metaphases per 23 examined). (E) mFISH and mBAND showed an inverted insertion of chromosome 20 (ins(20)(p11.2q13.3q12)) in TALEN-mediated knockin clone TKI^{I17}.

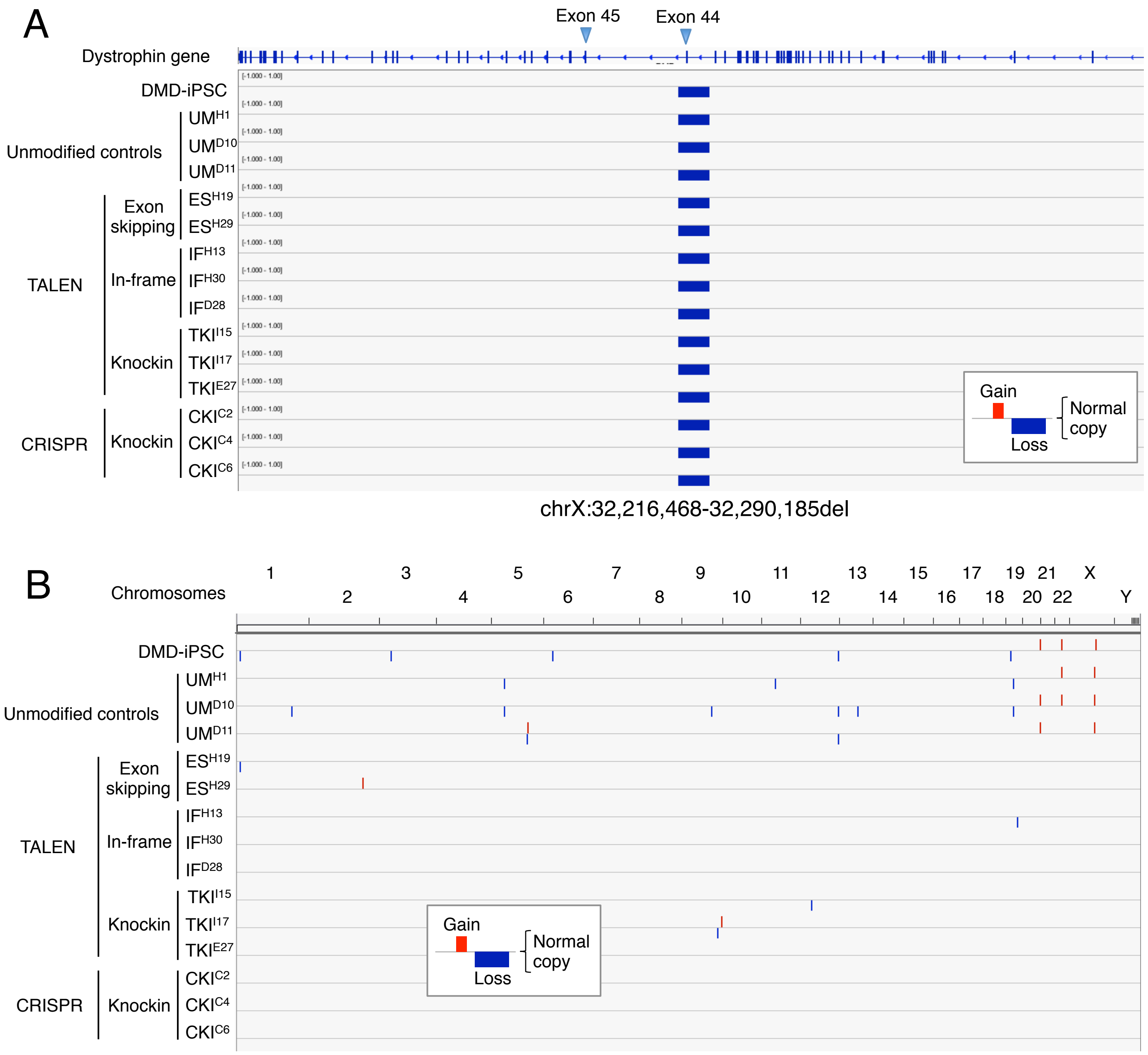

Figure S6. Detection of CNVs in the dystrophin gene and in the corrected clones, Related to Figure 5B.

(A) The copy numbers detected by the SNP array and PennCNV are plotted. The grey horizontal axis indicates normal copies (i.e. two copies for autosomal chromosomes and one copy for X and Y chromosomes). The blue bar under the grey axis indicates the copy number loss at the exon 44 region in the dystrophin gene. We successfully identified the deletion of chrX:32,216,468-32,290,185 in all DMD samples, which is in close agreement with break point analysis (chrX: 32,215,020-32,290,503del), as shown in Supplementary Fig. 1a. (B) De novo CNVs found in genetically modified clones. Red bars above the grey axis indicate copy number gain, and blue bars below the grey axis indicate copy number loss.

Table S1. Summary of unique *k*-mers to identify unique regions in the genome, Related to Figure 1.

Length	Total No. of *k*-mers		No. of unique *k*-mers	Coverage of the human genome
16-mer	4^{16}	4,294,967,296	980,117,801	62.60%
15-mer	4^{15}	1,073,741,824	263,051,237	27.60%
14-mer	4^{14}	268,435,456	55,041,124	7.29%
13-mer	4^{13}	67,108,864	5,842,203	1.13%
12-mer	4^{12}	16,777,216	324,003	0.08%
11-mer	4^{11}	4,194,304	3,097	9.61×10^{-4} %
10-mer	4^{10}	1,048,576	1	2.82×10^{-7} %

Table S2. Examples of the microhomology-mediated deletion patterns, Related to Figure 3.

Nucleases	Target sequences	Microhomology motif	Deletion size (bp)
Golden-TALEN	TTTTGCCTTTTTGGTATCTTACAGGAACTCCAGGATGGCATTGGGCAGCGGCAAACTGTTGTCAGAAC	GGCA	Δ 6
	TTTTGCCTTTTTGGTATCTTACAGGAACTCCAGGATGGCATTGGGCAGCGGCAAACTGTTGTCAGAAC	GGCA	Δ 7
	TTTTGCCTTTTTGGTATCTTACAGGAACTCCAGGATGGCATTGGGCAGCGGCAAACTGTTGTCAGAAC	GGCA	Δ 13
	TTTTGCCTTTTTGGTATCTTACAGGAACTCCAGGATGGCATTGGGCAGCGGCAAACTGTTGTCAGAAC	TGG	Δ 6
	TTTTGCCTTTTTGGTATCTTACAGGAACTCCAGGATGGCATTGGGCAGCGGCAAACTGTTGTCAGAAC	CAG	Δ 15
	TTTTGCCTTTTTGGTATCTTACAGGAACTCCAGGATGGCATTGGGCAGCGGCAAACTGTTGTCAGAAC	TTG	Δ 18
Platinum-TALEN	TTTTGCCTTTTTGGTATCTTACAGGAACTCCAGGATGGCATTGGGCAGCGGCAAACTGTTGTCAGAAC	GGCA	Δ 6
	TTTTGCCTTTTTGGTATCTTACAGGAACTCCAGGATGGCATTGGGCAGCGGCAAACTGTTGTCAGAAC	GGCA	Δ 7
	TTTTGCCTTTTTGGTATCTTACAGGAACTCCAGGATGGCATTGGGCAGCGGCAAACTGTTGTCAGAAC	GGCA	Δ 13
	TTTTGCCTTTTTGGTATCTTACAGGAACTCCAGGATGGCATTGGGCAGCGGCAAACTGTTGTCAGAAC	TGG	Δ 6
	TTTTGCCTTTTTGGTATCTTACAGGAACTCCAGGATGGCATTGGGCAGCGGCAAACTGTTGTCAGAAC	CAG	Δ 15
	TTTTGCCTTTTTGGTATCTTACAGGAACTCCAGGATGGCATTGGGCAGCGGCAAACTGTTGTCAGAAC	TTG	Δ 18
CRISPR-sgRNA1	TTTTGCCTTTTTGGTATCTTACAGGAACTCCAGGATGGCATTGGGCAGCGGCAAACTGTTGTCAGAAC	CAGGA	Δ 9
CRISPR-sgRNA2	TTTTGCCTTTTTGGTATCTTACAGGAACTCCAGGATGGCATTGGGCAGCGGCAAACTGTTGTCAGAAC	CAGGA	Δ 9
CRISPR-sgRNA3	TTTTGCCTTTTTGGTATCTTACAGGAACTCCAGGATGGCATTGGGCAGCGGCAAACTGTTGTCAGAAC	GGCA	Δ 6
	TTTTGCCTTTTTGGTATCTTACAGGAACTCCAGGATGGCATTGGGCAGCGGCAAACTGTTGTCAGAAC	TGG	Δ 6
	TTTTGCCTTTTTGGTATCTTACAGGAACTCCAGGATGGCATTGGGCAGCGGCAAACTGTTGTCAGAAC	CAG	Δ 15
CRISPR-sgRNA4	TTTTGCCTTTTTGGTATCTTACAGGAACTCCAGGATGGCATTGGGCAGCGGCAAACTGTTGTCAGAAC	GGCA	Δ 6
	TTTTGCCTTTTTGGTATCTTACAGGAACTCCAGGATGGCATTGGGCAGCGGCAAACTGTTGTCAGAAC	GGCA	Δ 7
	TTTTGCCTTTTTGGTATCTTACAGGAACTCCAGGATGGCATTGGGCAGCGGCAAACTGTTGTCAGAAC	GGCA	Δ 13
CRISPR-sgRNA5	TTTTGCCTTTTTGGTATCTTACAGGAACTCCAGGATGGCATTGGGCAGCGGCAAACTGTTGTCAGAAC	TTTTG	Δ 8
	TTTTGCCTTTTTGGTATCTTACAGGAACTCCAGGATGGCATTGGGCAGCGGCAAACTGTTGTCAGAAC	CTT	Δ 11

Red characters: microhomology sequences with more than 3 bp that flank the cleavage site of TALENs or CRISPRs.
Underline: the recognition site for TALEN pairs or CRISPR-sgRNAs
v: the cleavage sites of CRISPR-Cas9.

Table S3. Summary of capture statistics for the exome sequencing, Related to Figure 5C.

Category		Sample ID	Total Reads	Remove identical reads*	Unique %	Mapped reads†	Mapping %
Original fibroblast		DMD_fibroblast	129,536,708	88,652,535	68%	86,671,306	97.8%
Original iPSC clone		DMD-iPSC	130,243,436	106,064,367	81%	104,115,285	98.2%
Unmodified controls		UM^{D10}	134,171,856	90,013,937	67%	87,747,006	97.5%
		UM^{D11}	138,880,388	114,021,013	82%	112,671,565	98.8%
		UM^{H1}	126,398,760	109,834,280	87%	107,919,254	98.3%
TALEN	Exon skipping	ES^{H19}	136,945,384	104,830,953	77%	102,317,225	97.6%
		ES^{H29}	122,965,492	107,018,775	87%	104,802,925	97.9%
	In-frame	IF^{H13}	119,170,318	94,297,902	79%	92,796,375	98.4%
		IF^{H30}	138,612,424	109,896,988	79%	108,131,267	98.4%
		IF^{D28}	131,120,892	108,044,479	82%	106,164,805	98.3%
	Knockin	TKI^{I15}	95,030,774	80,900,233	85%	79,835,405	98.7%
		TKI^{I17}	97,704,786	82,501,149	84%	81,278,499	98.5%
		TKI^{E27}	92,361,384	78,223,262	85%	77,118,236	98.6%
CRISPR	Knockin	CKI^{C2}	119,453,700	107,111,909	90%	105,433,227	98.4%
		CKI^{C4}	130,291,792	113,680,521	87%	112,100,032	98.6%
		CKI^{C6}	125,818,120	111,141,505	88%	109,767,667	98.8%

* Duplicated identical reads were deemed as PCR artifact during the sample preparation of the sequencing.
† Mapped to human genome (hg19)

Table S4. SNVs detected by the exome sequencing, Related to Figure 5C.

UM^{H1}	UM^{D10}	UM^{D11}	ES^{H19}	ES^{H29}	IF^{H13}	IF^{H30}	IF^{D28}	TKI^{I15}	TKI^{I17}	TKI^{E27}	CKI^{C2}	CKI^{C4}	CKI^{C6}	chr	Position	Gene	ExonicFunc	ref	alt
									SNV					chr1	10725468	*CASZ1*	synonymous SNV	C	T
SNV									SNV					chr1	26671594	*AIM1L*	nonsynonymous SNV	T	C
SNV									SNV					chr1	26671595	*AIM1L*	synonymous SNV	G	A
SNV							SNV							chr1	47685584	*TAL1*	synonymous SNV	T	C
		SNV												chr1	47685593	*TAL1*	synonymous SNV	A	C
								SNV						chr1	152084216	*TCHH*	nonsynonymous SNV	C	G
					SNV									chr1	152084437	*TCHH*	nonsynonymous SNV	A	T
					SNV									chr1	152084438	*TCHH*	nonsynonymous SNV	G	C
						SNV			SNV					chr1	152681689	*LCE4A*	synonymous SNV	G	C
											SNV			chr1	228335240	*GUK1*	nonsynonymous SNV	G	A
					SNV	SNV								chr2	31457663	*EHD3*	nonsynonymous SNV	C	T
								SNV						chr2	204306002	*RAPH1*	synonymous SNV	A	G
								SNV						chr2	204306008	*RAPH1*	synonymous SNV	A	G
								SNV	SNV					chr3	45801382	*SLC6A20*	synonymous SNV	C	T
	SNV													chr3	75786748	*ZNF717*	nonsynonymous SNV	C	T
	SNV													chr3	167217960	*WDR49*	synonymous SNV	C	T
	SNV													chr3	167217964	*WDR49*	nonsynonymous SNV	A	G
				SNV										chr3	195506245	*MUC4*	nonsynonymous SNV	C	A
				SNV										chr3	195506645	*MUC4*	nonsynonymous SNV	G	A
											SNV			chr3	195515290	*MUC4*	nonsynonymous SNV	C	G
SNV														chr3	197500320	*FYTTD1*	nonsynonymous SNV	C	A
											SNV			chr4	88537180	*DSPP*	synonymous SNV	T	C
										SNV				chr5	140531592	*PCDHB6*	nonsynonymous SNV	C	T
									SNV					chr5	141974916	*FGF1*	nonsynonymous SNV	G	T
									SNV					chr6	16327915	*ATXN1*	nonsynonymous SNV	A	C
											SNV	SNV		chr6	25850031	*SLC17A3*	NA	A	G
SNV				SNV	SNV				SNV				SNV	chr6	57393112	*PRIM2*	NA	T	C
	SNV													chr6	57398264	*PRIM2*	synonymous SNV	A	G
													SNV	chr7	5413826	*TNRC18*	nonsynonymous SNV	T	G
										SNV				chr7	56087399	*PSPH*	nonsynonymous SNV	G	A
	SNV													chr7	89937168	*C7orf63*	synonymous SNV	G	A
										SNV				chr7	100639153	*MUC12*	nonsynonymous SNV	C	T
									SNV					chr8	101718965	*PABPC1*	nonsynonymous SNV	G	A
									SNV					chr8	101718968	*PABPC1*	nonsynonymous SNV	C	T
											SNV			chr8	101719004	*PABPC1*	nonsynonymous SNV	G	A
					SNV									chr8	133051259	*OC90*	nonsynonymous SNV	C	A
							SNV							chr8	144995598	*PLEC*	synonymous SNV	G	A
								SNV						chr9	35906583	*HRCT1*	nonsynonymous SNV	T	A
									SNV					chr9	124914613	*NDUFA8*	synonymous SNV	C	T
									SNV					chr9	125143792	*PTGS1*	synonymous SNV	C	A
					SNV	SNV								chr9	129455586	*LMX1B*	nonsynonymous SNV	C	T
								SNV		SNV				chr11	1090928	*MUC2*	nonsynonymous SNV	A	C
											SNV	SNV	SNV	chr11	62652670	*SLC3A2*	synonymous SNV	C	T
SNV														chr12	4737745	*AKAP3*	nonsynonymous SNV	C	A
					SNV	SNV								chr12	120117794	*PRKAB1*	synonymous SNV	G	A
SNV														chr12	122255756	*SETD1B*	nonsynonymous SNV	G	C
SNV														chr12	130921739	*RIMBP2*	nonsynonymous SNV	G	A
SNV														chr13	20797129	*GJB6*	nonsynonymous SNV	G	A
								SNV						chr14	70924501	*ADAM21*	nonsynonymous SNV	C	G
									SNV					chr14	104145852	*KLC1*	synonymous SNV	G	A
								SNV	SNV					chr15	43700161	*TP53BP1*	nonsynonymous SNV	T	C
				SNV										chr15	65678318	*IGDCC4*	nonsynonymous SNV	T	G
				SNV				SNV						chr16	1307050	*TPSD1*	synonymous SNV	C	T
				SNV				SNV						chr16	1307060	*TPSD1*	nonsynonymous SNV	A	G
										SNV				chr16	3026805	*PKMYT1*	nonsynonymous SNV	G	A
					SNV									chr16	66788886	*CCDC79*	nonsynonymous SNV	G	T
								SNV	SNV					chr17	3638150	*ITGAE*	nonsynonymous SNV	C	A
					SNV	SNV								chr17	9503461	*WDR16*	synonymous SNV	G	T
								SNV						chr17	21318698	*KCNJ12,KCNJ18*	nonsynonymous SNV	C	T
											SNV	SNV	SNV	chr17	66036857	*KPNA2*	nonsynonymous SNV	C	A
							SNV							chr19	1430261	*DAZAP1*	synonymous SNV	G	C
				SNV										chr19	5789565	*DUS3L*	nonsynonymous SNV	T	C
										SNV				chr19	7935879	*FLJ22184*	synonymous SNV	G	A
													SNV	chr19	35843086	*FFAR1*	nonsynonymous SNV	G	A
												SNV		chr19	40392347	*FCGBP*	nonsynonymous SNV	C	G
											SNV		SNV	chr19	40392360	*FCGBP*	nonsynonymous SNV	G	A
				SNV										chr19	27251	*KIR2DL2*	nonsynonymous SNV	C	T
	SNV													chrX	114425196	*RBMXL3*	nonsynonymous SNV	G	A
	SNV													chrX	114425210	*RBMXL3*	synonymous SNV	G	A
									SNV					chrX	118603830	*SLC25A5*	synonymous SNV	A	G
									SNV					chrX	118603844	*SLC25A5*	nonsynonymous SNV	T	G
9	7	1	0	8	9	5	3	12	17	6	8	4	6						

Total No. of detected SNVs per each clone.

Table S5. Indels detected by the exome sequencing, Related to Figure 5C.

UM^{H1}	UM^{D10}	UM^{D11}	ES^{H19}	ES^{H29}	IF^{H13}	IF^{H30}	IF^{D28}	TKI^{I15}	TKI^{I17}	TKI^{E27}	CKI^{C2}	CKI^{C4}	CKI^{C6}	chr	start	ref	alt	Gene	Comment
				Indel					Indel			Indel		chr12	7045891	–	CAGCAGCAG	*ATN1*	Triple repeat
						Indel								chr12	7045891	–	CAGCAGCAGCAG	*ATN1*	Triple repeat
													Indel	chr19	36002421	–	CTGCTGCCA	*DMKN*	Triple repeat
												Indel	Indel	chr19	36002421	–	CTGCTGCTG	*DMKN*	Triple repeat
											Indel	Indel		chr20	60891763	C	–	*LAMA5*	
		Indel	Indel		Indel	Indel			Indel	Indel	Indel			chr22	29885581	24 bp	–	*NEFH*	
											Indel	Indel		chr22	41573513	AG	–	*EP300*	
					Indel	Indel	Indel							chrX	31986615	–	T	*DMD*	On-target
			Indel	Indel										chrX	31986615	18 bp	–	*DMD*	On-target
0	0	1	1	1	1	2	0	0	2	1	3	4	2						

Total No. of detected Indels per clone.

24 bp: AGGCCAAGTCCCCAGAGAAGGAAG
18 bp: ATGCCATCCTGGAGTTCC

Table S6. List of primers used, Related to the Experimental Procedures.

Targets	Primers	Sequence
qRT-PCR for pluripotency markers	hACTB-Fwd	CCAACCGCGAGAAGATGA
	hACTB-Rev	CCAGAGGCGTACAGGGATAG
	hOCT3/4-Fw	GACAGGGGGAGGGGAGGAGCTAGG
	hOCT3/4-Rv	CTTCCCTCCAACCAGTTGCCCCAAAC
	hNANOG-Fw	TGAACCTCAGCTACAAACAG
	hNANOG-Rv	TGGTGGTAGGAAGAGTAAAG
Construction of sgRNAs	DMD-sgRNA1-fwd	GAGACCACTTGGATCCGggtatcttacaggaactccGTTTTAGAGCTAGAAATAGCA
	DMD-sgRNA2-fwd	GAGACCACTTGGATCCGtcttacaggaactccaggaGTTTTAGAGCTAGAAATAGCA
	DMD-sgRNA3-fwd	GAGACCACTTGGATCCGaggaactccaggatggcatGTTTTAGAGCTAGAAATAGCA
	DMD-sgRNA4-fwd	GAGACCACTTGGATCCGCCAGGATGGCATTGGGCAGGTTTTAGAGCTAGAAATAGCA
	DMD-sgRNA5-fwd	GAGACCACTTGGATCCGTTCCTGTAAGATACCAAAAGTTTTAGAGCTAGAAATAGCA
	sgRNA-Universal-rev	GCCCGGGTTTGAATTCAAAAAAAGCACCGACTCGGTGCCACTTTTTCAAGTTGATAA CGGACTAGCCTTATTTTAACTTGCTATTTCTAGCTCTAA
Construction of Donor template	DMD-Donor-F1_FW	ACCGCGGTGGCGGCCGCTTGCTATTGTGTCAAGG
	DMD-Donor- F1_RV_2	GCTCGCCGGCAAAACAAAAATGAAGCC
	DMD-Donor-F2_FW_2	GTTTTGCCGGCGAGCTCAGACGATAA
	DMD-Donor-F2_RV_2	GTAAAACATCGCGCAACGCAATTAGT
	DMD-Donor-F3_FW_2	TGCGCGATGTTTTACATAATCCATCTATTTT
	DMD-Donor-F3_RV	GGAGTTCCTTAAGATACCATTTGTATTTAG
	DMD-Donor-F4_FW_2	GCCAGACCGTCGTGAGAACATTGAATGCAACTGG
	DMD-Donor-F4_RV	CGGTACCCGGGGATCCAAGAGCTTGGCAAAAGAAC
Sanger sequencing for dystrophin exon 45	DMD-screening-fwd	CACCTCTCGTATCCACGATCACTAAG
	DMD-screening-rev	TAGTGCCTTTCACCCTGCTTAT
Deep sequencing for dystrophin exon 45	DMD-MiSeq-Rd1-fwd-X	CTCTTTCCCTACACGACGCTCTTCCGATCTXXXXAATAAAAAGACATGGGGCTTCA
	DMD-MiSeq-Rd2-rev-X	CTGGAGTTCAGACGTGTGCTCTTCCGATCTXXXXCTGGCATCTGTTTTTGAGGA
	Multiplex P5 fwd	AATGATACGGCGACCACCGAGATCTACACTCTTTCCCTACACGACGCTC
	Multiplex P7 rev	CAAGCAGAAGACGGCATACGAGATGTGACTGGAGTTCAGACGTGTGCTC
qPCR for residual episomal vectors	hFbx15-2F	GCCAGGAGGTCTTCGCTGTA
	hFbx15-2R	AATGCACGGCTAGGGTCAAA
	EBNA-183F	ATCAGGGCCAAGACATAGAGATG
	EBNA-243R	GCCAATGCAACTTGGACGTT
Screening knockin clones	P1	AGCTTTACCTCAGTCTCAGAAAACA
	P2	ACCCGTTGCGAAAAAGAACGTT
	P3	AGAATTGGGAACATGCTAAATACAA
	P4	TTGGATTAGATTGAGCCTAGTTCAG
	DMD-In45-F	CCCATGATTGCTTAAAGGTGA
	DMD-In45-R	AGAGCTTGGCAAAAGAACGA
Off-target analysis	gRNA-DMD1-OT1Fwd	TATATGGAAGCGTGGTCTGTGATAATT
	gRNA-DMD1-OT1Rev	TCCCTTTTATCAGCTAGGGCTCTTA
	gRNA-DMD1-OT2Fwd	GTCCAAGAACCGGCCAGAACTCAG
	gRNA-DMD1-OT2Rev	CCATCCATTCCCCAATAAAGCAAGGC
	gRNA-DMD1-OT3Fwd	AAAATGTTGAGAATTGCCTATTTGA
	gRNA-DMD1-OT3Rev	GATTCTTGCCAATTCTCTTTACACA
	gRNA-DMD1-OT4Fwd	CCTCCTCTCTTTTGTTGTATCTTCTCT
	gRNA-DMD1-OT4Rev	TTTCCATTTCACAAACCATTAACTC
	gRNA-DMD1-OT5Fwd	AGGGCTGATTATATTAGGAGAGTGG
	gRNA-DMD1-OT5Rev	AAAAATTTCCCATCTAGGAAGTGAG
cDNA analysis for dystrophin expression	exon 43	ACAAAGCTCAGGTCGGATTG
	exon 46	AGTTGCTGCTCTTTTCCAGGT

Supplemental Experimental Procedures

DNA construction

The expression plasmids of Golden-TALENs shown in Figure S2A were constructed using the Golden-Gate cloning method as described previously (Sakuma et al., 2013a). Platinum-TALEN expression plasmids were constructed using the Platinum-Gate system with non-RVD (repeat variable di-residue) variations (Sakuma et al., 2013b).

The Cas9 cDNA sequence (SphcCas9) from *Streptococcus pyogenes* was codon-optimized for human codon usage bias, and possible *cis*-acting motifs (i.e. splicing sites, poly A sites and destabilizing motifs) were removed by DNA synthesis (GenScript). The synthesized SphcCas9 cDNA was cloned into pHL-EF1α-GW-iP-A (Addgene 60599) by the Gateway LR reaction to construct the pHL-EF1α-SphcCas9-iP vector, which contained an EF1α promoter for high expression in human iPS cells. To construct custom sgRNA expression vectors, two oligos containing the sgRNA target site and a universal reverse primer (Table S6) were PCR amplified and cloned into the BamHI-EcoRI site of the pHL-H1-ccdB-mEF1α-RiH vector (Addgene 60601), which used the H1 promoter to drive sgRNA. The resultant sgRNA expression vectors pHL-H1-sgRNA[DMD1~3]-mEF1α-RiH are available from Addgene (ID 60602-60604).

The target region that contained all TALEN pairs and sgRNAs was cloned into the SSA vector, in which the target region was flanked by the truncated luciferase gene with a homologous region (Sakuma et al., 2013a). A donor template vector for exon 44 knockin of the dystrophin gene (pENTR-DMD-Donor, Addgene 60605) was constructed by conjugating the following four fragments: two homology arms (1 kbp for 3' and 0.9 kbp for 5' arm), an EF1α-hygromycin cassette, and exon 44 fragments, all of which were combined using the In-Fusion HD Cloning Kit (Clontech).

The oligos and primers used are listed in Table S6. For electroporation experiments, plasmid DNAs were purified by a NucleoBond Xtra Maxi Endotoxin-free plasmid DNA purification Kit (TaKaRa) and were prepared as high concentration stock solution of at least 4 μg μl^{-1}.

Cell culture

HEK293T (human embryonic kidney 293T) cells were cultured in Dulbecco's modified Eagle's medium (DMEM) supplemented with 5% FBS (fetal bovine serum), 1× non-essential amino acids (NEAA), 100 units ml^{-1} penicillin and 100 μg ml^{-1} streptomycin. Human iPS cells were cultured on mitomycin-C-treated SNL feeder layers in Knockout SR Media {DMEM/F12 media supplemented with 20% Knockout SR, L-glutamine, NEAA, 2-mercaptoethanol, penicillin and streptomycin (Life Technologies), and 8 ng ml^{-1} human basic FGF (Wako)). SNLs were maintained in DMEM containing 6% FBS, 100 units ml^{-1} penicillin, 100 μg ml^{-1} streptomycin and 2 mM GlutaMax. SNL-PH feeder cells (resistant to neomycin, puromycin, and hygromycin) were used for drug selection experiments.

Flow Cytometry

iPS cells were first treated with CTK solution to remove feeder cells, and then single cells were isolated by treatment with 0.25% trypsin-EDTA. Cells were stained with the following antibodies in FACS buffer (5% FBS in PBS) for 20 min at room temperature. The antibodies used were Alexa Fluor 488-conjugated TRA-1-60 (1:20, BD Pharmingen, Cat No. 560174) and FITC-conjugated SSEA-5 (1:20, BioLegend, Cat No. 355207). Unstained cells were used as a negative control.

Quantitative RT-PCR

mRNA was extracted from cells using TRIzol (Invitrogen) reagent and purified by chloroform extraction and isopropanol precipitation. After DNase treatment, the purified RNA was eluted in RNase-Free water and reverse-transcribed using ReverTra Ace (TOYOBO) with random primers and oligo-dT. To quantify the expression of *OCT3/4* and *NANOG*, the level of synthesized cDNAs was measured in isolated samples using SYBA Green (Invitrogen) with the appropriate primers shown in Table S6.

Teratoma formation assay

All mouse experiments were carried out according to protocols approved by the Animal Research Committee of Kyoto University (No. KEI47). For teratoma formation,

sub-confluent human iPS cells were harvested and re-suspended in a 1:2 mixture of culture medium and Matrigel (BD) with 10 μM Y-27632. Around 2 x 10^6 human iPS cells were injected into the tibialis anterior muscles of NOD/SCID/DMD-null mice (NOD/SCID mice crossed with DMD-null mice (Tanaka et al., 2013)). The resulting tumors were harvested from mice 8-9 weeks after transplantation. Samples were fixed in 4% formaldehyde and embedded in paraffin. The sections were stained with hematoxylin and eosin.

Southern blot analysis

Genomic DNA was isolated by phenol-chloroform extraction and ethanol precipitation. Genomic DNAs (15 μg each) were digested with EcoRI for 16 hours, applied on 0.8% agarose gels, and then transferred onto nylon membranes with 20x SSC. The DNA probe (intron 45, 519 bp) was DIG-labeled with the DIG Easy Hyb Kit (Roche) using the pENTR-DMD-Donor as a template with DMD-In45-F and DMD-In45-R primers. DNA hybridization and signal detection were carried out using DIG Easy Hyb and the DIG Chemiluminescent Detection System (ImageQuant LAS 4000 system (GE)).

T7 endonuclease I assay

The target region of TALEN and CRISPR was amplified by a high-fidelity PCR reaction from genomic DNA and purified by Wizard SV Gel and the PCR Clean up System (Promega). The primer sequences for PCR are shown in Table S6. Purified PCR products (400 ng) were denatured (95 ºC for 5 min) and re-annealed (gradually cooled from 95 ºC to 85 ºC at –2 ºC sec^{-1} and 85 ºC to 25 ºC at –0.1 ºC sec^{-1}) in NEBuffer 2 (NEB) using a thermocycler. The re-annealed PCR product was digested by 10 units of T7 endonuclease I (T7EI, NEB) for 15 min at 37 °C. The reaction was stopped by the addition of 0.25 M EDTA solution, and the sample was placed on ice. The PCR products were analyzed on 2% agarose gel to semi-quantify the intensity of the digested and undigested bands by ImageJ software.

Restriction enzyme sensitivity assay

Purified PCR products (generated the same way as in the T7EI assay) were digested by restriction enzyme XcmI (NEB) with NEBuffer 2. The amounts of digested and

undigested PCR products were analyzed by 2% agarose gel electrophoresis. The rate of the area of nuclease-specific cleavage peaks in the sum of the area (expressed as a fraction cleaved) was used to estimate the gene editing levels using the following equation (Reyon et al., 2012).

$$\% \text{ mutation} = 100 \times (1-(1-\text{fraction cleaved})^{1/2})$$

Karyotype analysis

Exponentially growing iPS cell samples (at passage 7 for the original DMD-iPSC clone and at passage 15-23 for the corrected clones) were arrested in metaphase by adding a final concentration of 0.04 μg ml^{-1} KaryoMAX Colcemid Solution (Life technologies) into the culture medium for 3 h at 37 °C. Then, the cells were dissociated with 0.25% trypsin-EDTA solution and pelleted by centrifugation. The pelleted cells were subjected to hypotonic treatment with 0.075 M KCl solution for 30 min at 37 °C. After fixing the cells with fresh methanol/acetic acid (3:1) mixture, metaphase spreads were prepared on pre-cleaned glass slides by dropping a few drops of fixed cells from 1 cm height. Samples were then air dried. Conventional Giemsa staining was used for chromosomal counts and classifications, and the GTG (G-bands after trypsin and Giemsa) technique was used for G-banding. Slides were observed by AxioImagerZ2 microscopy (CarlZeiss) equipped with a CoolCube1m CCD camera (MetaSystems) and Ikaros software (MetaSystems). A total of 50 metaphases were counted for the chromosome counting, and 20 metaphases were observed for the G-band analysis per sample. The cells were described as abnormal if at least two nuclei were found to have the same chromosome aberration. Any samples suspected to be abnormal were further analyzed by multicolor fluorescent *in situ* hybridization (mFISH) analysis using the 24XCyte mFISH Probe Kit (MetaSystems) or by multicolor chromosome banding (mBAND) analysis using an appropriate XCyte 20 mBAND Probe Kit (MetaSystems) for chr20. Fluorescent images were acquired with Isis software (MetaSystems).

SNP array for the DNA copy number analysis

Genomic DNA was isolated from iPS cell samples at passage 20-27 after feeder depletion by Matrigel culture and purified by the DNeasy Blood and Tissue Kit (QIAGEN). SNP genotyping was performed on Illumina HumanOmniExpress 12v1

following the manufacturer's protocol. PennCNV (Wang et al., 2007) software was used to detect CNVs using the following criteria: confidence score > 10, number of SNPs > 10 and minimum length of structural variant > 1 kb. Original CNVs (those called in the original DMD-fibroblasts and uncorrected DMD-iPSCs) and common CNVs (those detected in more than 7 subclones out of the 15 clones analyzed) were discarded. The fragmented CNVs were merged by the clean_cnv.pl script using PennCNV software. We manually checked the values of Log R ratio and B allele frequency at the detected CNVs by IGV software. BEDTools was used to calculate the distance from the potential nuclease target sites to the edge of the detected CNVs and to select random positions in the genome.

Exome sequence analysis

The same genomic DNA samples prepared above were used for exome capture with SeqCap EZ v3 (Roche) following the manufacturer's instructions with minor modification. Briefly, 1 μg of genomic DNA was randomly fragmented by the Covaris E210 system. Adaptor-ligated genomic DNA libraries were prepared with the TruSeq DNA Sample Preparation Kit (Illumina) according to the manufacturer's instructions. The libraries were amplified by the ligation-mediated polymerase chain reaction (LM-PCR) and purified with AMPure XP beads (Agencourt). Human COT-1 DNA and Illumina adaptor-specific blocking oligonucleotides were used during hybridization to block repetitive genomic sequences and adaptor sequences, respectively. The exome-enriched libraries were amplified by LM-PCR, purified with the AMPure XP beads and quantified by a KAPA library quantification Kit (KAPA Biosystems). Ten pM of libraries were used in the cluster generation on HiSeq PE Flow Cell v3 (Illumina) using an Illumina cBot. Paired-end sequencing for 2 × 101 cycles was performed using TruSeq SBS Kit v3 and HiSeq2500 (Illumina). CASAVA 1.8.2 (Illumina) was used to generate FASTQ files from the raw sequencing data.

The sequenced reads in the FASTQ files were mapped to the reference human genome (hg19) by BWA 0.6.2, and identical reads (i.e. PCR duplicates) were removed by Picard 1.68. All samples were generated within 92-120 million reads and had mapping rates over 92% (Table S3). Local re-alignment at the detected indel site was performed by the Genome Analysis Toolkit (GATK) v1.6. Base quality scores were recalibrated with GATK TableRecalibration script. SNVs and indels were called with

GATK UnifiedGenotyper according to the Broad Institute's best-practice v3 guidelines.

The number of nucleotides with a count greater than 30× coverage in the founder cells and iPS clones were subjected to SNV calling using SAMtool mpileup v0.1.19. We discarded variants with lower coverage (< 20 reads in autosomal chromosomes or < 10 reads in sex chromosomes). We calculated the nucleotide composition ratio of each SNV to discriminate homozygous (if one base consisted of more than 77.7%) and heterozygous (if the base compositions of two respective bases were in the range of 22.2-66.6%) SNVs. For indel calling, we considered whether > 40% of sequence reads supported the same indels. We also excluded the common SNVs/indels seen in all samples and the original SNVs/indels derived from the original DMD fibroblasts or DMD-iPSCs. We used ANNOVAR (Wang et al., 2010) to annotate the detected SNVs using the RefSeq, Cosmic ver.65 (Forbes et al., 2011), HGMD (Stenson et al., 2003) and dbSNP ver.137 (Sherry et al., 2001) databases.

Immunocytochemistry

Cell samples seeded on cover slips were fixed with chilled methanol for 15 min and blocked by Blocking One (Nacalai) for 30 min at 4 °C. Cells were then stained with the appropriate primary antibodies for 16 hours at 4 °C and then with secondary antibodies for 1 hour at room temperature. Coverslips were then washed and observed in 1x PBS by confocal microscopy (Zeiss LSM 710). The primary antibodies used were: NCL-DYS1 (1:20; clone Dy4/6D3, Leica), MHC (1:400; R&D systems), MYH (1:200; clone H-300, Santa Cruz Biotechnology) and α-skeletal muscle actin (1:200; Acris Antibodies). The secondary antibodies used were Alexa fluor 488 goat-anti-mouse (1:500; Invitrogen) for dystrophin protein and Alexa fluor 546 goat-anti-rabbit (1:500; Invitrogen) for MHC or MYH. DAPI (4,6-diamidino-2-phenylindole; Invitrogen) was used for nuclear counterstaining.

Western blot analysis

Cell samples were lysed with RIPA buffer (Thermo) supplemented with a protease inhibitor cocktail (Calbiotech). The total amount of protein was quantified by the Pierce BCA Protein Assay Kit (Thermo). Samples were then mixed with loading buffer and heated at 95 °C for 10 min. Approximately 40 μg of protein samples per lane were separated on NuPAGE 3-8% Tris-Acetate Gel (Invitrogen) with NuPAGE®

Tris-Acetate SDS running buffer (Invitrogen) and transferred to nitrocellulose membranes using iBlot (Invitrogen) for 13 min. Then, membranes were blocked by Blocking One for 1 hour at room temperature. The primary antibodies used were anti-dystrophin (1:200, Abcam) and anti-MHC (1:400, R&D systems). The secondary antibodies used were anti-rabbit IgG HRP-linked antibody and anti-mouse IgG HRP-linked antibody (1:1000, Cell Signaling). The protein expression was detected using the ImageQuant LAS 4000 system (GE).

Supplemental References

Forbes, S., Bindal, N., Bamford, S., Cole, C., Kok, C., Beare, D., Jia, M., Shepherd, R., Leung, K., Menzies, A., *et al.* (2011). COSMIC: mining complete cancer genomes in the Catalogue of Somatic Mutations in Cancer. Nucleic Acids Res *39*, D945-950.

Reyon, D., Tsai, S., Khayter, C., Foden, J., Sander, J., and Joung, J. (2012). FLASH assembly of TALENs for high-throughput genome editing. Nat Biotechnol *30*, 460-465.

Sakuma, T., Hosoi, S., Woltjen, K., Suzuki, K., Kashiwagi, K., Wada, H., Ochiai, H., Miyamoto, T., Kawai, N., Sasakura, Y., *et al.* (2013a). Efficient TALEN construction and evaluation methods for human cell and animal applications. Genes Cells *18*, 315-326.

Sakuma, T., Ochiai, H., Kaneko, T., Mashimo, T., Tokumasu, D., Sakane, Y., Suzuki, K., Miyamoto, T., Sakamoto, N., Matsuura, S., *et al.* (2013b). Repeating pattern of non-RVD variations in DNA-binding modules enhances TALEN activity. Sci Rep *3*, 3379.

Sherry, S., Ward, M., Kholodov, M., Baker, J., Phan, L., Smigielski, E., and Sirotkin, K. (2001). dbSNP: the NCBI database of genetic variation. Nucleic Acids Res *29*, 308-311.

Stenson, P., Ball, E., Mort, M., Phillips, A., Shiel, J., Thomas, N., Abeysinghe, S., Krawczak, M., and Cooper, D. (2003). Human Gene Mutation Database (HGMD): 2003 update. Hum Mutat *21*, 577-581.

Tanaka, A., Woltjen, K., Miyake, K., Hotta, A., Ikeya, M., Yamamoto, T., Nishino, T., Shoji, E., Sehara-Fujisawa, A., Manabe, Y., *et al.* (2013). Efficient and reproducible myogenic differentiation from human iPS cells: prospects for modeling Miyoshi Myopathy in vitro. PLoS One *8*, e61540.

Wang, K., Li, M., Hadley, D., Liu, R., Glessner, J., Grant, S., Hakonarson, H., and Bucan, M. (2007). PennCNV: an integrated hidden Markov model designed for high-resolution copy number variation detection in whole-genome SNP genotyping data. Genome Res *17*, 1665-1674.

Wang, K., Li, M., and Hakonarson, H. (2010). ANNOVAR: functional annotation of genetic variants from high-throughput sequencing data. Nucleic Acids Res *38*, e164.
